# Integrated proteome and phosphoproteome analyses of peripheral blood mononuclear cells in primary Sjögren syndrome patients

**DOI:** 10.18632/aging.202233

**Published:** 2020-12-03

**Authors:** Shaoying Huang, Fengping Zheng, Lixiong Liu, Shuhui Meng, Wanxia Cai, Cantong Zhang, Weier Dai, Dongzhou Liu, Xiaoping Hong, Donge Tang, Yong Dai

**Affiliations:** 1 Department of Clinical Medical Research Center, Guangdong Provincial Engineering Research Center of Autoimmune Disease Precision Medicine, Shenzhen People’s Hospital, The First Affiliated Hospital Southern University of Science and Technology, The Second Clinical Medical College of Jinan University, Shenzhen 518020, Guangdong, China; 2 Department of Rheumatology and Immunology, The Second Clinical Medical College of Jinan University, The First Affiliated Hospital of Southern University of Science and Technology, Shenzhen People’s Hospital, Shenzhen 518020, Guangdong, China; 3 Guangxi Key Laboratory of Metabolic Disease Research, Nephrology Department of Guilin, Guilin 541002, China; 4 College of Natural Science, University of Texas at Austin, Austin, TX 78712, USA

**Keywords:** primary Sjögren syndrome, peripheral blood mononuclear cells, phosphoproteome, biomarker

## Abstract

Primary Sjögren syndrome (pSS) is a common autoimmune disease. Here, we performed the first proteome and phosphoproteome analyses of peripheral blood mononuclear cells in pSS patients to obtain a comprehensive profile and identify the potential crucial proteins and pathways for the screening and evaluation of pSS patients. Peripheral blood mononuclear cells from 8 pSS-confirmed patients (American-European Consensus Group Criteria, 2002) and 10 normal controls were selected. Label-free quantitative proteomics was utilized to obtain quantitative information. In total, 787 proteins were identified as differentially expressed proteins, and 175 phosphosites on 123 proteins were identified as differentially phosphorylated proteins. We performed functional enrichment analyses with these proteins and phosphoproteins based on public database. Furthermore, protein-protein interaction network analyses were performed by using multiple algorithms. Using module and hub protein analyses, we identified 16 modules for the proteins, 2 clusters for the phosphoproteins and selected the top 10 hub proteins. Finally, we identified 22 motifs using motif analysis of the phosphosites and found 17 newly identified motifs, while 6 motifs were experimentally verified for known protein kinases. The findings distinguished pSS patients from normal controls at the peripheral blood mononuclear cells level and revealed potential candidates for use in pSS diagnosis.

## INTRODUCTION

Primary Sjögren syndrome (pSS) is a chronic autoimmune disease with lymphocytic infiltration and epithelial cell destruction but no association with other autoimmune diseases (e.g., scleroderma, systemic lupus erythematosus or rheumatoid arthritis) [1]. Clinically, it often invades the salivary gland and lacrimal gland, where it manifests as xerostomia of the mouth and eyes [1], which might severely decrease patients’ quality of life. More seriously, the function of exocrine glands, such as those in the respiratory system, digestive system, skin and vagina, are also often damaged, and lesions outside the glands can also be seen [1–3]. Patients with visceral damage mostly achieve remission after proper treatment, but they can also relapse after treatment withdrawal [4]. Furthermore, patients with progressive pulmonary fibrosis, renal tubular acidosis, central neuropathy, pulmonary hypertension, lymphoma in visceral damage, and acute pancreatitis have a poor prognosis [4]. With an overall prevalence of 0.1-0.6% in the adult population [5], nearly 0.1% in Europe [6] and 0.33-0.77% in China [7], no disease-modifying drug is currently available for the disease, and the current treatment for pSS focuses only on relieving symptoms, which is unsatisfactory [8].

Early diagnosis ensures timely management of both the clinical presentation and complications. The revised American-European classification criteria (AECG) requires a positive minor gland biopsy sample, autoantibodies, and symptoms with oral and ocular dryness for the diagnosis of SS [9]. Typical symptoms, however, may occur several months to years before the ultimate diagnosis. Therefore, serological markers are desperately needed, such as anti-Ro/SSA [10] and anti-La/SSB [11], for a provisional diagnosis. However, the autoantibody markers currently available cannot satisfy the need for specificity and/or sensitivity for a confirmatory diagnosis [12]. Additionally, labial salivary gland biopsy has become the most widely accepted test to confirm pSS. However, its limitations are obvious; for example, a biopsy is an invasive method, and there is no observable lymphocytic infiltration in some of the confirmed subjects [13]. Consequently, there is an unmet need for noninvasive clinical tests to identify pSS.

The emerging thoughts on precision medicine underscores the importance of quantitative portrayals of molecular features. Notably, peripheral blood mononuclear cells (PBMCs) consist of immune cells, including natural killer (NK) cells from the innate immune system or lymphocytes from the adaptive immune system or both systems (monocytes and dendritic cells). Thus, PBMCs have attracted increasing attention as intermediaries in autoimmune disease and proteomic studies, which may facilitate the investigation of etiopathogenesis factors or predictors of pSS. Compared with biopsy, PBMCs can be conveniently and safely obtained by isolation from peripheral blood. In addition, genomic data provide a considerable amount of information to decipher regarding molecular machinery. However, the expression and functional details of genes are often best reflected by the presence (or absence) of proteins acting as executors of physiological functions. Protein phosphorylation is a reversible posttranslational modification that is also one of the major mechanisms of signal integration and is essential for the regulation and maintenance of most biological processes in eukaryotes [14]. Phosphorylation is employed by cells to transiently alter protein properties such as the activities of enzymes and interactions with other proteins [15]. Thus, we took the initiative to perform the first analyses of the PBMC proteome and phosphoproteome of pSS.

In this study, differentially expressed proteins (DEPs) and differentially phosphorylated proteins (DPPs) were identified by label-free quantitative shotgun proteomic analyses, and phosphopeptide enrichment was determined using immobilized metal affinity chromatography (IMAC) using PBMCs. The elaborate bioinformatics strategies, including Gene Ontology (GO) analysis, Kyoto Encyclopedia of Genes and Genomes (KEGG) signaling pathway analysis, motif analysis, and protein-protein interaction (PPI) network analysis were utilized to analyze the data and reveal pSS-related modulation and pSS signatures both in the proteome and phosphoproteome, thereby contributing to an initial PBMC-based map of pSS. The novel DEPs and DPPs identified in the PBMCs of pSS may shed light on biomarkers useful for disease screening and the underlying molecular mechanisms for use in eventually tackling pSS.

## RESULTS AND DISCUSSION

### Qualitative and quantitative proteomic profiling

**Figure 1:**
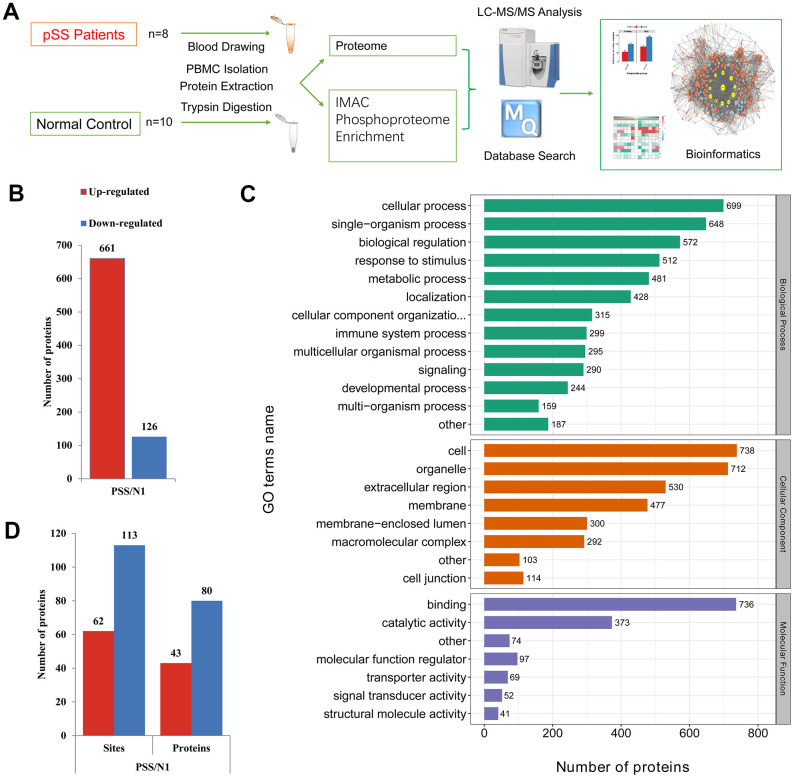
(**A**) The schematic flow to study the proteome and phosphoproteome of peripheral blood mononuclear cells (PBMCs) in primary Sjögren syndrome (pSS) patients. (**B**) Histogram of quantity distribution of differentially expressed proteins (DEPs). (**C**) Gene Ontology (GO) classifications of the DEPs of PBMCs in pSS patients based on biological process, cellular component and molecular function. (**D**) Histogram of quantity distribution of differentially expressed phosphorylated proteins (DPPs). IMAC: immobilized metal affinity chromatography. LC-MS: liquid chromatography–mass spectrometry.

The schematic flow is shown in Figure 1A. Based on a shotgun proteomics approach, 2517 proteins were identified in the study based on 12692 unique peptides and a maximum false positive rate (FDR) < 1%, among which 1111 proteins were at a quantifiable level in the pSS or NC-pSS groups (Supplementary Table 1). The combined results were used for the following analyses. Considering the criterion of ratio change > 2-fold or < 0.5-fold for proteins to be considered significantly differentially expressed, 787 proteins were identified as differentially expressed proteins (DEPs). Among these proteins, 661 were up-regulated, and 126 were down-regulated in the pSS group (Figure 1B). The top 20 proteins up-regulated in pSS are listed in Table 1. These 20 proteins showed the most significant differences in the quantitative comparison of healthy volunteers and pSS patients at the PBMC level and, therefore, appeared to be the potential discriminatory biomarkers for pSS.

**Table 1:** The summary of top 20 upregulated differentially expressed proteins.

**Protein accession**	**Gene name**	**PSS/N1 Ratio**	**Regulated Type**
Q13813	SPTAN1	61.5	Up
O43150	ASAP2	43.444	Up
Q9UBW5	BIN2	23.691	Up
Q9Y2A7	NCKAP1	20.978	Up
O43149	ZZEF1	20.277	Up
P42025	ACTR1B	18.802	Up
P09960	LTA4H	17.519	Up
P48637	GSS	16.094	Up
P48059	LIMS1	14.748	Up
Q9C0I1	MTMR12	14.625	Up
P05556	ITGB1	14.385	Up
Q9Y3P9	RABGAP1	14.152	Up
O75791	GRAP2	13.599	Up
Q05193	DNM1	12.699	Up
Q4KMQ2	ANO6	12.333	Up
Q9C0C9	UBE2O	12.158	Up
Q96KP4	CNDP2	11.821	Up
P80511	S100A12	11.739	Up
O95373	IPO7	11.739	Up
P52788	SMS	11.422	Up

Additionally, there were some preliminary proteins that were not classified as DEPs under the criterion of ratio change > 2-fold, such as HLA-DRA (ratio: 1.751) and HLA-DRB1 (ratio: 1.528), but were considered as previous potential biomarkers for Sjögren syndrome. Studies have shown that the major histocompatibility complex (MHC) gene frequency in Sjögren syndrome patients increased, and the positive rate of HLA-B8, DR3 and DRw52 gene expression was significantly higher than that in the normal subjects [4]. We identified HLA-B (ratio: 10.429, up-regulated) and HLA-A (ratio: 6.38, up-regulated) in the PBMC proteome, which corroborated the high frequency of the MHC genes found in pSS patients. HLA-A is associated with increased susceptibility to multiple autoimmune diseases, such as multiple sclerosis (MS) [16] and Behcet disease (BD) [17]. HLA-A has not been proven to be associated with pSS and is reported in the PBMC proteome level for the first time.

### GO classification of the DEPs

The GO classifications of the proteins in the pSS proteomes are shown in Figure 1C. The proteins presented a similar distribution of DEPs in pSS based on the GO classification. In the cellular component classification, most of the proteins were in the cell, organelle, extracellular region, membrane, and macromolecular complex categories. In the molecular function classification, the majority of the proteins were in the binding, catalytic activity, and molecular function regulator categories. In the biological process classification, most proteins were observed to be involved in cellular processes, single-organism processes, and biological regulation. Notably, the immune system process category contained 299 DEPs (6%). Among them, LTA4H (ratio: 17.519, up-regulated), S100A12 (ratio: 11.739, up-regulated), CD36 (ratio: 6.194, up-regulated), and PRKCB (ratio: 4.682, up-regulated) being involved in inflammatory processes and immune response caught our attention. LTA4H catalyzes the final step in the biosynthesis of the proinflammatory mediator leukotriene B4 [18]. S100A12 plays a prominent role in the regulation of inflammatory processes and immune response. Its proinflammatory activity involves recruitment of leukocytes, promotion of cytokine and chemokine production, and regulation of leukocyte adhesion and migration [19]. CD36 is a multifunctional glycoprotein that acts as a receptor for a broad range of ligands. Ligands can be of proteinaceous nature like TLR4, which promotes inflammation in monocytes/macrophages [20]. PRKCB is a protein kinase involved in regulation of the B-cell receptor (BCR) signalosome, and plays a key role in B-cell activation [21]. These proteins were specifically up-regulated, suggesting a role of immunology and inflammation in the PBMC proteome of pSS.

### GO, KEGG and protein domain functional enrichment analyses and clustering analysis of the DEPs

**Figure 2:**
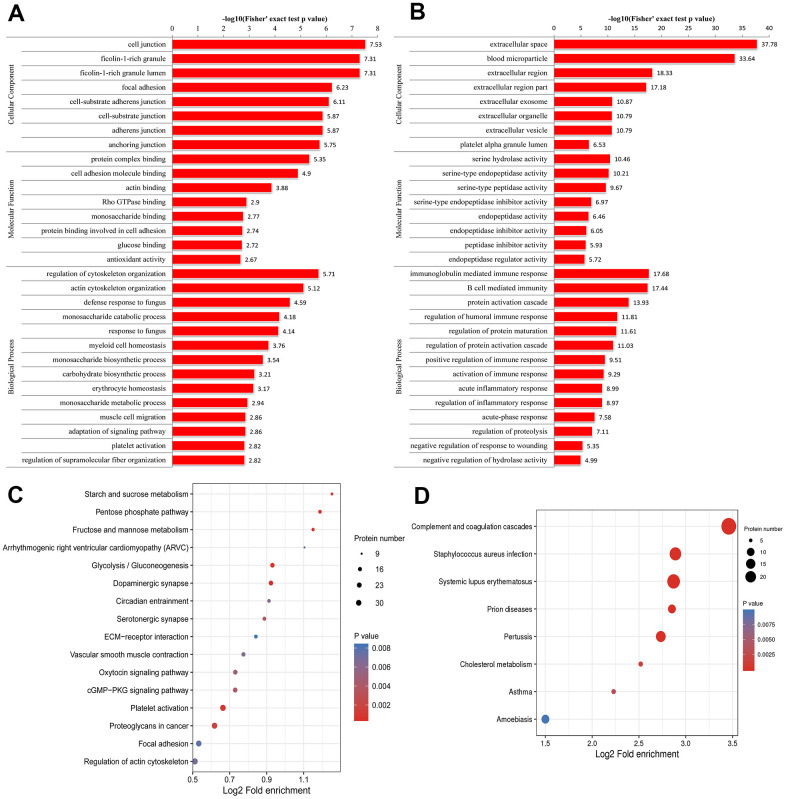
**Gene Ontology (GO) enrichment analysis of peripheral blood mononuclear cells (PBMCs) in primary Sjögren syndrome (pSS) patients based on biological process, cellular component and molecular function.** (**A**) GO enrichment analysis of up-regulated differentially expressed proteins (DEPs). (**B**) GO enrichment analysis of down-regulated DEPs. (**C**) Kyoto Encyclopedia of Genes and Genomes (KEGG) functional enrichment analysis of up-regulated differentially expressed proteins (DEPs). (**D**) KEGG functional enrichment analysis of down-regulated DEPs.

The results of the GO enrichment analysis are shown in Figure 2A and 2B. Regarding the enrichment of proteins based on biological process, negative regulation of hydrolase activity and regulation of cytoskeleton organization were significantly enriched. Regarding the enrichment of the proteins that were up-regulated in the pSS group based on biological processes, the regulation of cytoskeleton organization and actin cytoskeleton organization were enriched. Regarding the enrichment of the proteins that were down-regulated in the pSS group based on biological process, the categories of protein activation cascade, immunoglobulin mediated immune response, B-cell-mediated immunity, regulation of protein maturation, regulation of humoral immune response, regulation of protein activation cascade, acute inflammatory response, regulation of inflammatory response, activation of immune response, and positive regulation of immune response were enriched.

Regarding the enrichment of proteins that were up-regulated in the pSS group based on molecular function, categories such as protein complex binding, actin binding and cell adhesion molecule binding were enriched. In the molecular function classification, the peptidase inhibitor activity, serine-type endopeptidase inhibitor activity, endopeptidase regulator activity, and enzyme inhibitor activity categories had a significant abundance in pSS down-regulated proteins.

In the cellular component classification, the cytoplasmic vesicle lumen, vesicle lumen, and secretory granule lumen categories had a significant abundance of pSS proteins. Regarding the enrichment of the proteins that were up-regulated in the pSS group based on the cellular component classification, categories such as ficolin-1-rich granules were enriched. According to the enrichment of the proteins that were down-regulated in the pSS group based on the cellular component classification, categories such as extracellular space, blood microparticle, and extracellular region were enriched.

For KEGG enrichment of DEPs, we identified 16 pathways from up-regulated DEPs (Figure 2C) and 8 pathways from down-regulated DEPs (Figure 2D). The pathway of complement and coagulation cascades (hsa04610) was substantially down-regulated as an immunological pathway among the identified pathways. All of the significantly enriched pathways, namely, cGMP-PKG signaling pathway (hsa04022), arrhythmogenic right ventricular cardiomyopathy (ARVC) (hsa05412), hypertrophic cardiomyopathy (HCM) (hsa05410), and vascular smooth muscle contraction (hsa04270) pathways, seemed to be related to the cardiovascular system, and the cardiovascular risk increased in pSS, although the underlying mechanisms remain unclear [22]. It is not clear whether these pathways play direct roles in the mechanisms of pSS.

The protein domain refers to specific components that appear repeatedly in different protein molecules, which have similar sequences, structures and functions and are the units conserved during protein evolution. The length of the domain is usually between 25 and 500 amino acids in length. We identified the enrichment of the significant protein domains in up-regulated DEPs, such as thioredoxin domain, thioredoxin-like fold, EF-hand domain, and pleckstrin homology domain.

Finally, we divided all of the identified proteins into four quantiles (Q1-Q4) according to fold change: Q1 (0 < ratio < 0.5), Q2 (0.5 < ratio < 0.67), Q3 (1.5 < ratio < 2.0), and Q4 (ratio > 2.0) and performed functional enrichment clustering analyses (Supplementary Figures 1–5).

### PPI network of the DEPs

Genes causing related diseases tend to cluster with one another in an interaction network of proteins or functions [23]. Therefore, the PPI network identifies and characterizes related protein complexes, which are crucial for understanding the molecular events involved [24]. We determined the PPI relationships using STRING [25]. The interactions in the STRING database were based on coexpression, text mining, databases, experiments, cooccurrence, neighborhoods, and gene fusion [26]. To show the interaction between the proteins clearly, we selected the top 50 proteins with the closest interactions and generated a network with a total number of 395 associated proteins. We selected the top 10 proteins as hub proteins based on degree (Table 2), which often play important roles in network stability because of their high degree of connections/interactions. We found that 8 out of the top 10 are up-regulated and involved in immune system processes.

**Table 2:** Top 10 hub proteins in PPI network based on degree.

**Gene name**	**Degree**	**Betweenness**	**Centroid**	**Closeness**	**Bridging**
UBA52	75	23277.55	-22	0.001153	32.12191
MAPK1	69	14795.83	9	0.001176	28.44867
HSPA8	56	14824.35	-14	0.001145	56.68246
ACTB	55	11072.69	-23	0.001142	40.24189
ACLY	52	14155.31	-159	9.90E-04	35.44649
APP	49	7938.799	-44	0.001119	36.50353
ACTN1	49	5651.568	-20	0.001144	38.92992
ACTN4	47	5555.22	-22	0.00114	43.87646
VWF	46	3615.55	-104	0.001048	35.02035
TGFB1	41	8255.129	-19	0.001151	58.37043

Degree, betweenness, centroid, closeness and bridging are algorithms provided by plugin Centiscape in software Cytoscape.

MAPK1 (ratio: 2.676, up-regulated) had a high degree of 69 of interrelatedness, suggesting its potential for having an important role in pSS. MAPK1 is a member of a superfamily of mitogen-activated protein kinases (MAPKs), which control a variety of functions in eukaryotic cells [27, 28]. Altered MAPK signaling has been associated with autoimmune diseases such as lupus [29]. However, the association between MAPK signaling and pSS has been poorly characterized.

TGFB1 (ratio: 3.819, up-regulated) is a multifunctional protein that regulates the growth and differentiation of various cell types and is involved in various processes, such as normal development and immune function [30]. It was involved in inflammatory bowel disease (IBD) with impaired T-cell response to stimulation and decreased T-cell subsets [31]. Interestingly, peripheral blood T cell reduction is one of the most prominent characteristics of immune abnormalities in pSS patients. However, the relationship between TGFB1 and pSS has not been well elucidated yet.

### Module analyses

**Figure 3:**
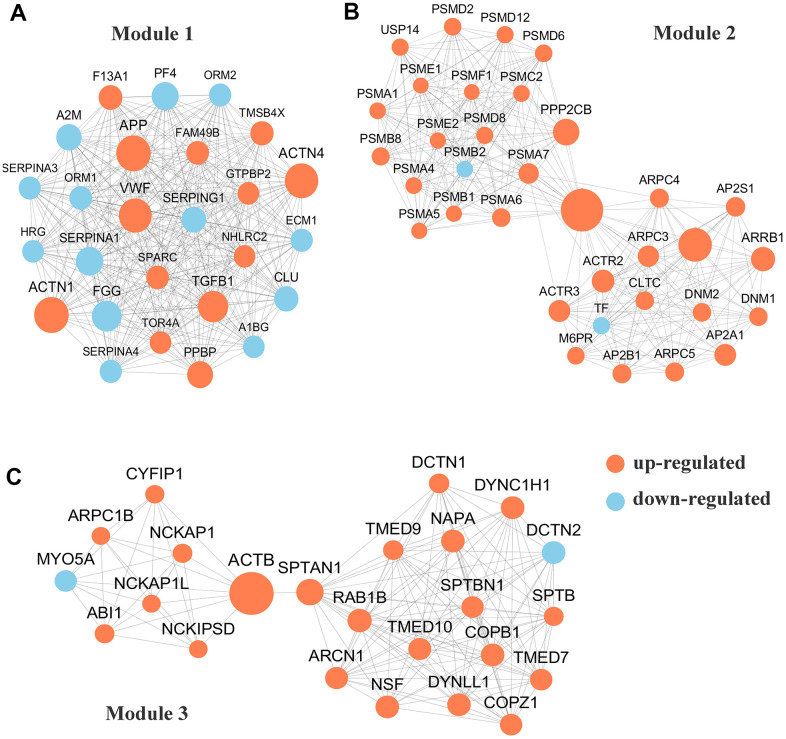
**Protein-protein interaction (PPI) network analyses of differentially expressed proteins (DEPs) were performed and 3 most significant modules were yielded by molecular complex detection (MCODE) algorithm.** Red and light cyan indicate up- and down-regulated differentially expressed proteins (DEPs), respectively. (**A**). Module 1 (score =33.000) was constructed with 26 nodes and 325 edges. (**B**) Module 2 (score =17.758)) was constructed with 34 nodes and 293 edges. (**C**) Module 3 (score =12.261) was constructed with 24 nodes and 141 edges.

To conduct PPI topological analysis, we combined the DEPs that are known biomarkers of pSS and applied them to the molecular complex detection (MCODE) plugin in Cytoscape [31]. MCODE is one of the most commonly used PPI clustering algorithms and can isolate dense regions and predict protein complexes via a PPI subnetwork based on connectivity data [25, 32]. MCODE yielded 16 modules, and the 3 most significant DEP modules were extracted. Module 1 (MCODE score =26.000) was constructed with 26 nodes and 325 edges (Figure 3A); module 2 (MCODE score =17.758) was constructed with 34 nodes and 293 edges (Figure 3B); and module 3 (MCODE score =12.261) was constructed with 24 nodes and 141 edges (Figure 3C).

### Proteome-wide analyses of the phosphorylated PBMCs in the pSS patients

The global phosphoproteomic profiles from the PBMCs of the pSS patients had not been previously reported. In this study, we performed quantitative phosphoproteomic analyses of the PBMCs from the pSS patients.

### Identification of phosphopeptides

In total, the experiment revealed the identification of 4349 peptides. Of all the acquired peptides, 2148 phosphopeptides were obtained. To maintain high localization confidence, the data were filtered with the combined cutoff values for the identification of the localization probability > .75. In total, 2449 unique phosphosites on 1239 proteins were identified, among which the quantitative information of 175 sites on 123 proteins was obtained. For all the identified phosphosites, 84.95% were phosphorylated at a serine residue, 13.58% at a threonine residue and 1.47% at a tyrosine residue. The serine and threonine distribution of phosphosites in our study was consistent with that of the results from human phosphoproteomes determined *in vivo*, while our tyrosine phosphorylation proportion was slightly higher [15].

Differential phosphorylation may be the effect of changes in protein expression or changes of a phosphosite. To discern the cause of the differential findings, we normalized the site ratio with the protein ratio to determine the relative change in phosphorylation site stoichiometry based on the quantified proteomic data obtained, which facilitated the discernment of the changes from the modification itself and those in the pathways that modulate pSS [32]. The results were used for the following analyses. For the quantitative analysis, 62 phosphosites on 43 phosphoproteins were determined to be up-regulated while 113 on 80 phosphoproteins were down-regulated, considering the ratio change greater than 2-fold indicated upregulation while that < 2-fold indicated down-regulation, which suggested molecular diversity in the PBMCs of the pSS group and would be gathered to conduct the following bioinformatics analysis (Figure 1D). The down-regulated phosphoproteins were obviously more common than the up-regulated phosphoproteins, in contrast to the ratio observed in the proteome assessed in the current study. These identified phosphoproteins might be crucial and valuable factors in the mechanism of pSS.

### GO and subcellular localization classification of the phosphoproteins

To investigate the phosphoproteome in pSS, we performed GO classification of the phosphoproteins. The biological process and cellular component categorization of the differentially regulated phosphoproteins revealed a similar enrichment pattern as that for the pSS proteome. The three largest classes of biological processes were cellular processes (12%), single-organism processes (12%) and biological regulation (10%). Cell (21%), organelle (19%), and membrane (17%) were the three major categories in the cellular component classification. Based on the molecular function classification, the three largest categories included binding (49%), catalytic activity (21%) and molecular function regulator (11%). Shown by the analysis of subcellular localization, the 439 quantifiable phosphoproteins were distributed in the cytoplasm (37%), nucleus (27%) and plasma membrane (11%).

### GO and KEGG functional enrichment analyses of the phosphoproteins

A GO enrichment analysis was performed to better understand the functions of the identified phosphoproteins. According to biological process enrichment classification, regulation of ion transmembrane transport, protein complex assembly, secretion, and homotypic cell-cell adhesion were greatly enriched in up-regulated phosphoproteins. In contrast, platelet activation, blood coagulation, and cell morphogenesis involved in differentiation were greatly enriched in down-regulated phosphoproteins. In agreement with this observation, the molecular function enrichment analysis showed significant enrichment of binding categories such as kinase binding, identical protein binding, enzyme binding, integrin binding, and mitogen-activated protein kinase binding for the up-regulated phosphoproteins. For the down-regulated phosphoproteins, calcium channel regulator activity, protein complex binding, macromolecular complex binding, and cell adhesion molecule binding were highly represented. Accordingly, in the analysis of cellular component enrichment classification, the highly enriched categories included the adherens junction, anchoring junction, cell-substrate adherens junction, focal adhesion, cell-substrate junction, secretory vesicle, and transport vesicle categories for the up-regulated phosphoproteins. For the down-regulated phosphoproteins, the cytoskeleton, vesicle, contractile fiber part, myofibril and cell junction categories were highly represented.

Site occupancies of phosphorylation correlate with the cellular signaling state [15]. A KEGG pathway analysis was performed on the DPPs to evaluate the pathways that were significantly represented in pSS. In the KEGG enrichment analysis of the phosphoproteins, there was no pathway which would represent up-regulated phosphoproteins. Compared with the proteome data, the phosphoproteome data showed the down-regulated phosphorylation of the signaling pathways in pSS, including the pathways of gap junctions, focal adhesion, phagosomes, the VEGF signaling pathway, and the inflammatory mediator regulation of TRP channels. Genes encoding interepithelial junctional proteins in adherens and gap junctions suggested perturbations in the permeability between epithelial barriers. Focal adhesion was associated with infiltrating leukocytes and was reported as an initial biomarker of possible autoimmunity, for the first time in 2011 in the lacrimal glands of mice [33].

### Motif analysis of the phosphosites

Protein phosphorylation modifications are regulated by protein kinases (PKs), and different PKs prefer specific substrates with conserved motifs. We took advantage of the large number of phosphorylation sites identified in this study to carry out a bioinformatics analysis to identify novel phosphorylation motifs. We carried out an intensive sequence analysis for overrepresented phosphorylation site motifs surrounding the identified phosphosites (10 amino acids upstream and 10 amino acids downstream of each phosphosite) of the serine and threonine residues using the Motif-X program.

**Figure 4:**
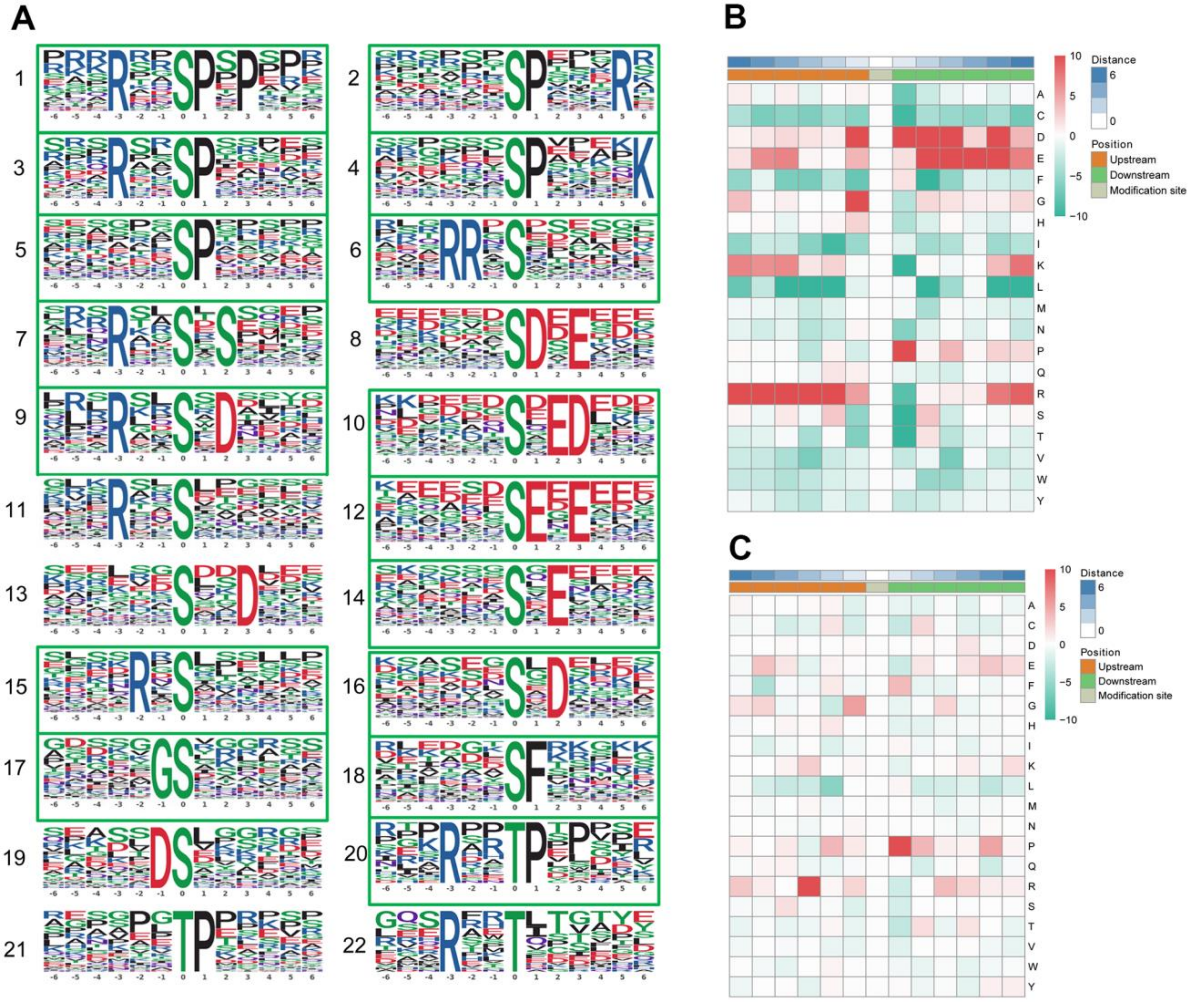
**Motif analysis of the phosphosites.** (**A**) Significantly enriched phosphorylation motifs extracted from the overrepresented phosphopeptide dataset by Motif-X. (1) -(19) Motifs from phosphoserine; (20) -(22) Motifs from phosphothreonine. Among these motifs, 6 are identified as 5 known phosphorylation motifs and 17 are newly identified (enclosed into green boxes). The detailed information and putative associated kinases are shown in Table 3. Motif enrichment heat map of phosphoserine (**B**) and phosphothreonine (**C**) upstream and downstream of all identified phosphorylation modification sites. Red indicates significant enrichment of the amino acid near the modification site, while green indicates significant reduction of the amino acid near the modification site.

We identified 19 conserved motifs based on 1787 phospho-serine (pS) phosphosites, and 4 conserved motifs were identified based on 130 phospho-threonine (pT) phosphosites (Figure 4A). Because of the small number in the tyrosine group, no obvious conserved motif was acquired through our sequence analysis. In particular, [xxxRxx_pS_PxPxxx] (motif 1), [RRx_pS_xxxxxx] (motif 6), [xxxxxx_pS_DxExxx] (motif 8), and [xxxxxx_pS_ExExxx] (motif 12) (motif score > 30.00) were strikingly conserved. Intriguingly, the motif analysis of the corresponding phosphosites found that the proline-directed motif [pS/pT_P] was increased significantly, whereas the most common motif, [xxxRxx_pS_PxPxxx] (motif 1), was more statistically significant and enriched, with a motif score of 41.28 and a fold increase of 33.1. According to the Human Protein Reference Database (HPRD), 6 phosphorylation motifs were experimentally verified and specific for known PKs, whereas 17 had no known corresponding kinases [34]. The detailed information and putative associated kinases are shown in Table 3. Motifs [xxxxxx_pS_DxExxx] (motif 8) and [xxxRxx_pT_xxxxxx] (motif 22) were verified as casein kinase II (CKII) substrate motifs [35]. CKII is a ubiquitous eukaryotic kinase [36] and phosphorylates numerous substrates, including many transcription factors [37, 38]. Phosphorylation by CKII has been associated with direct replication defect [39], which may be a possible explanation for the differentiation found in the PBMC phosphoproteome of pSS. Motif [xxxxxx_pT_Pxxxxx] (motif 21) was verified as a WW domain-binding motif [40], which mediates protein-protein interactions via proline-rich regions [41].

**Table 3:** Phosphorylation motifs enriched by motif-x and putative protein kinases.

**Motif No.**	**Motif**	**Motif Score**	**Fold Increase**	**Putative Protein Kinases**
Serine motif				
1	xxxRxx_S_PxPxxx	41.28	33.1	Novel
2	xxxxxx_S_PxxxRx	29.46	9.7	Novel
3	xxxRxx_S_Pxxxxx	25.83	9.1	Novel
4	xxxxxx_S_PxxxxK	23.79	8.7	Novel
5	xxxxxx_S_Pxxxxx	16.00	3.5	Novel
6	xxxRRx_S_xxxxxx	30.52	11.7	Novel
7	xxxRxx_S_xSxxxx	22.42	7.6	Novel
8	xxxxxx_S_DxExxx	32.00	13.7	Casein kinase II substrate motif
9	xxxRxx_S_xDxxxx	22.01	10.4	Novel
10	xxxxxx_S_xEDxxx	24.68	7.1	Novel
11	xxxRxx_S_xxxxxx	16.00	3.9	Calmodulin-dependent protein kinase II substrate motif
12	xxxxxx_S_ExExxx	32.00	9.5	Novel
13	xxxxxx_S_xxDxxx	14.58	2.4	Pyruvate dehydrogenase kinase substrate motif
14	xxxxxx_S_xExxxx	16.00	2.3	Novel
15	xxxxRx_S_xxxxxx	11.21	2.3	Novel
16	xxxxxx_S_xDxxxx	9.18	2.2	Novel
17	xxxxxG_S_xxxxxx	7.48	2.0	Novel
18	xxxxxx_S_Fxxxxx	6.77	2.3	Novel
19	xxxxxD_S_xxxxxx	6.53	2.2	β-Adrenergic Receptor kinase substrate motif
Threonine motif				
20	xxxRxx_T_Pxxxxx	26.69	24.6	Novel
21	xxxxxx_T_Pxxxxx	16.00	3.8	WW domain binding motif
22	xxxRxx_T_xxxxxx	6.83	3.1	Calmodulin-dependent protein kinase II substrate motif

A heat map (Figure 4B) was generated to show the enrichment or depletion of specific amino acids neighboring the serine phosphosites. The amino acids aspartate (D), glutamate (E), glycine (G), proline (P) and arginine (R) had a tendency to be present in the proximity of serine phosphosites. The amino acids proline (P) and arginine (R) were greatly represented in the areas proximal to threonine phosphosites (Figure 4C). Arginine (R) was greatly represented in the sequence surrounding phosphosites but was greatly depleted in the -1 position. Cysteine (C), phenylalanine (F) and leucine (L) were notably depleted in the sequence surrounding phosphosites. These preferential amino acids near phosphosites reflect the specific recognition of the enzymes that catalyze phosphorylation in the PBMCs of pSS patients. Further studies are needed to investigate whether the different types of enzymes and kinases that regulate phosphorylation are active in the pathophysiology of pSS.

### PPI and module analysis of the phosphoproteins

**Figure 5:**
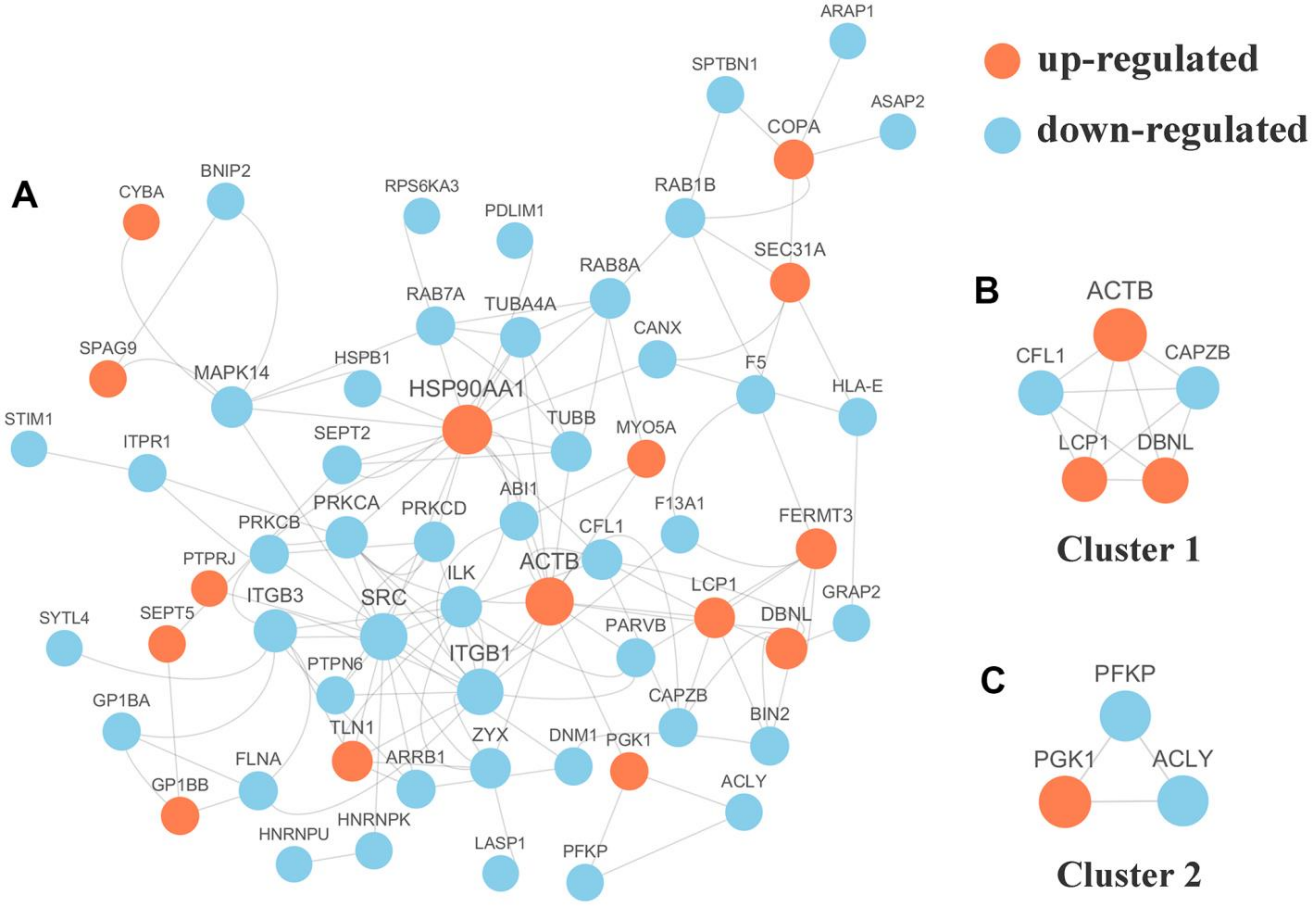
(**A**) Protein-protein interaction (PPI) network analyses of differentially expressed phosphorylated proteins (DPPs) were performed and 2 significant clusters were yielded by molecular complex detection (MCODE) algorithm. Red and light cyan indicate up- and down-regulated DPPs, respectively. Yellow indicates hub phosphorylated proteins based on degree. (**B**) Cluster 1 (MCODE score = 5.000) was constructed with 5 nodes and 10 edges. (**C**) Cluster 2 (MCODE score =3.000) was constructed with 3 nodes and 3 edges.

We selected the top 50 phosphoproteins with the closest interactions and generated a network with a total number of 58 associated phosphoproteins (Figure 5A). HSP90AA1, with 17 nodes, exhibited the most interactions among the phosphoproteins and was considered a hub phosphoprotein based on degree scores, which was valuable for other analyses in this study. Further analysis of the complex by MCODE revealed 2 subnetworks for the network Cluster 1 (MCODE score = 5.000) was constructed with 5 nodes and 10 edges (Figure 5B) and cluster 2 (MCODE score =3.000) was constructed with 3 nodes and 3 edges (Figure 5C).

## CONCLUSION

Based on proteome and phosphoproteome analyses, our results provide new insights into pSS and reveal many essential features of and differences in the PBMCs of pSS through a systematic bioinformatics analysis. In total, 787 proteins were identified as DEPs, and 175 phosphosites on 123 proteins were identified as DPPs. Using systematic bioinformatics analyses, we identified 10 hub proteins: *UBA52, MAPK1, HSPA8, ACTB, ACLY, APP, ACTN1, ACTN4, VWF*, and *TGFB1*. Finally, we identified 22 motifs using motif analysis of the phosphosites and found 17 newly identified motifs, while 6 motifs were experimentally verified for known protein kinases. This allowed us to conclude that a panel of candidate biomarkers rather than a single specific protein is apparently able to best distinguish pSS among individuals, including healthy volunteers, and when they have been further validated, these biomarkers may improve the clinical detection of primary SS.

The analyses of the DEPs and DPPs for which the expression may correlate with the molecular mechanisms of pSS provide novel insight into the roles of phosphorylation and the underlying cause or regulation of this disease. Additionally, the hub proteins, pathways and motifs identified might be therapeutic targets and potential discriminatory biomarkers for pSS diagnosis.

While our results are promising, we are aware of the limitations of our study. Our study was restricted to cell-lined signatures and was not further validated in the clinical setting. The lack of multiple biological replicates requires further experiments to consolidate the conclusions drawn from the proteomics study data. Future studies will test the ability of these biomarker levels, alone and in combination, to diagnose the PBMC component of pSS. Overall, this work provides a unique prospect and resource for future studies focusing on the PBMCs of pSS patients.

## MATERIALS AND METHODS

### Patient assessment and PBMC isolation

In Supplementary Table 2, the general clinical characteristics of the study subjects are summarized. Eight pSS patients fulfilling the revised 2002 American-European Consensus Group criteria were enrolled in the study [9]. All patients had the clinical symptoms of dry eyes and/or mouth and at least one of the qualifying anti-SS-related antigen A (Ro/SSA), anti-SS-related antigen La/SSB, or anti-nuclear antibodies and rheumatoid factor. Patients with HIV-1, hepatitis B and/or hepatitis C infections were excluded from the study. None of the patients had received specific treatment, such as glucocorticoid and/or biological therapy and/or immunosuppressive drug treatment at the time their blood was drawn for this study. In addition, ten normal controls (NC) (between 20 and 65 years old) who were enrolled in the Physical Examination Centre at the Shenzhen People’s Hospital for routine health examinations and without disease were included. The healthy subjects had no complaints related to oral dryness and autoimmune disease and had normal salivary function. Written informed consent was obtained from all patients and healthy volunteers before they were included in the study.

Blood samples from all patients were obtained before breakfast. We then took 4mL of each sample and it was diluted with an equal volume of normal saline. Each of these samples was mixed and added to a 6mL lymphocyte separation solution. The samples were immediately processed by density gradient centrifugation according to the standardized protocol of PBMC isolation to minimize the degradation of proteins. The samples were stored at -80° C until they were used in the proteomic analyses.

### Experimental procedures

### Materials and reagents

The materials and reagents required for the sample preparation are shown in Supplementary Table 3.

### Protein extraction

The PBMCs were removed from -80° C storage, and four volumes of lysis buffer (containing 8 mol/L urea, 1% protease inhibitors, and 1% phosphorylase inhibitor) was added to a high-intensity ultrasonic processor instrument (PTM Bio, Hangzhou, China), after which the residual cell components were removed immediately by centrifugation (12000 ×g at 4° C for 10 min). Then, the supernatant was transferred to a new tube, and the protein concentration was defined by a BCA kit according to the manufacturer’s instructions.

### Trypsin digestion

The protein solution was sequentially diluted (5 mmol/L dithiothreitol for 30 min at 56° C) and alkylated with 11 mmol/L iodoacetamide for 15 min. These procedures were performed in darkness at room temperature. Then, the assembled protein sample was diluted to a urea concentration of less than 2 mol/L. Finally, trypsin was added to initiate overnight digestion (the ratio of trypsin to the protein mass ratio was 1:50) at 37° C and a subsequent 4 h digestion (the ratio of trypsin to protein mass was 1:100).

### Affinity enrichment of the phosphopeptides

Peptide mixtures were first incubated with IMAC microsphere suspensions with vibration in loading buffer (50% acetonitrile/6% trifluoroacetic acid). The IMAC microspheres with enriched phosphopeptides were collected by centrifugation, and the supernatant was removed. To remove nonspecifically adsorbed peptides, the IMAC microspheres were washed sequentially with 50% acetonitrile/6% trifluoroacetic acid and 30% acetonitrile/0.1% trifluoroacetic acid sequentially. To elute the enriched phosphopeptides from the IMAC microspheres, an elution buffer containing 10% NH_4_OH was added, and the enriched phosphopeptides were eluted. Finally, the eluted fractions were combined and vacuum-dried. For LC-MS/MS analyses, the resulting peptides were desalted with C18 ZipTips (Millipore) according to the manufacturer’s instructions.

### LC-MS/MS analysis

The tryptic peptides were dissolved in an aqueous solution containing 0.1% formic acid (solvent A), and a NanoElite ultrahigh-performance liquid system was used for separation after dissolution. The gradient was composed of solvent B (0.1% formic acid in 98% acetonitrile) increased from 2% to 22% over 70 min for the proteome or 40 min for the phosphoproteome and from 22% to 37% (22% to 35% for the phosphoproteome) over 10 min and increasing to 80% over 5 min, then held at 80% for the final 5 min. For all steps, the flow rate was maintained at a constant 300 nL/min for the proteome and 250 nL/min for the phosphoproteome in an EASY-nLC 1000 UPLC system. The peptide was separated by an ultrahigh-performance liquid system, subjected to a capillary ion source for ionization, and then analyzed by timsTOF Pro mass spectrometry. The electrospray voltage was applied at 1.4 kV. TOF mass spectrometry was used to detect and analyze the peptide ions and their secondary fragments. The m/z scan range was 100 to 1700 for the secondary mass spectrometer. The data acquisition mode was based on the parallel cumulative serial fragmentation (PASEF) mode. After the acquisition of the first-level mass spectrometry data, the 10-fold PASEF mode was used to collect the second-level spectrum of the parent ion with a charge number in the range of 0-5. The dynamic exclusion time of tandem mass spectrometer scanning is set to 30 seconds to avoid repeated scanning of the parent ion.

### Database search

The resulting MS/MS data were processed using the MaxQuant search engine (v1.6.6.0). The retrieval parameter settings were as follows: the database was homo_sapiens_9606_SP_20190513 with 20422 sequences; an anti-library was added to calculate the FDR caused by random matches, and a common pollution library was added to the database to eliminate the influence of contaminated proteins in the identification results; the digestion method was set to Trypsin/P; the number of missing cut points was set to 2; the minimum length of the peptide was set to 7 amino acid residues; the maximum number of modifications of the peptide was set to 5; the tolerance values of the mass error for the primary precursor ion in the first search and main search were set to 40 ppm and 40 ppm, respectively, and the error tolerance of the mass of the secondary fragment ion was to 0.04 Da; the cysteine alkylation was specified as a fixed modification, and the oxidation of methionine and the acetylation of the N-terminus of the protein were specified as the variable modifications; and the FDR for the protein identification and peptide-spectrum matches identification was adjusted to 1%. The mass tolerance for the precursor ions was set as 20 ppm for the first search and 5 ppm in the main search, and the mass tolerance for the fragment ions was set as 0.02 Da. Carbamidomethyl on Cys was specified as a fixed modification, and acetylation modification and oxidation on Met were specified as variable modifications. The FDR was adjusted to < 1%, and the minimum score for modified peptides was set to be > 40. Only proteins with a fold change > 2 or < 0.5 and a p value < 0.01 were considered to exhibit a significant difference and were subjected to the subsequent bioinformatics analyses.

### Quality control

Most of the peptides were distributed between 7-20 amino acids in length, which conformed to the general rules based on trypsin enzymatic hydrolysis and met the requirements of quality control (Supplementary Figure 6A). Proteins below 10KD (which are more peptide-oriented) were lost due to multiple acetone precipitation and rinsing; the distribution of proteins above 10KD was relatively uniform, indicating that there was no significant molecular weight bias for proteins above 10KD in the sample preparation process. The protein above 100KD was not lost during the preparation process due to poor solubility (Supplementary Figure 6B). The coverage of most proteins was below 20%. In the mass spectrometry method based on the shotgun (also called bottom-up) strategy, the mass spectrometer preferentially scanned the peptides with higher abundance. Therefore, the coverage of the protein was positively correlated with its abundance in the sample (Supplementary Figure 6C). The molecular weight of the protein had a negative correlation with the coverage. The reason was that a protein with a larger molecular weight could theoretically produce more enzymatically digested peptides. To achieve the same coverage, large proteins required more peptides to be identified (Supplementary Figure 7A). The majority of the phosphorylated proteins carried 1 to 4 Khib sites, and approximately 6% of the proteins carried more than 4 Khib sites (Supplementary Figure 7B).

### Bioinformatics methods

For the annotation analyses, GO annotation of the proteome was based on the UniProt-GOA database (http://www.ebi.ac.uk/GOA/) with the IDs of the proteins found in this study of pSS; these proteins were then classified by GO annotation based on the following three categories: biological process, molecular function and cellular component. The functional protein domain descriptions were annotated based on the InterPro domain database (http://www.ebi.ac.uk/interpro/ and the protein sequence alignment. We also conducted KEGG pathway annotation using the KEGG database (http://www.genome.jp/kegg/), and WoLF PSORT (v.0.2 http://www.genscript.com/psort/wolf_psort.html) was used to predict and characterize the subcellular localization of the identified proteins. For the functional enrichment analyses of the proteins identified by GO, a two-tailed Fisher’s exact test was employed as a significance test to verify the enrichment of the DEPs for each GO annotation category in our study (p value < 0.01). For the functional enrichment analyses of the proteins identified in KEGG pathways, the KEGG database was used to identify the pathways enriched with the proteins and to test the enrichment of the DEPs compared to all the identified proteins. A two-tailed Fisher’s exact test was used, and the pathways with corrected p value < 0.01 were considered significant and were separated into individual categories. For the enrichment analyses of the protein domains, a two-tailed Fisher’s exact test was utilized to examine the enrichment of the DEPs compared to all the identified proteins. For enrichment-based clustering, we first collated all the categories based on the results from both enrichment analyses along with the respective p values for clustering analyses depending on the DEP functional classification (based on the GO, domain and pathway analyses). Then, we picked the categories with at least one enriched cluster and a p value <0.01. For the PPI network, the STRING database (version 10.5) was used, and a confidence score was defined to assess the interactions, with a confidence score >0.7 defined as a high confidence score. Finally, we integrated the databases and networks and used the MCODE algorithm to analyze the characteristics of the networks and find densely connected regions. For phosphoproteome, software MoMo (motif-x algorithm) was used to analyze the model of sequences constituted with amino acids in specific positions of modify-21-mers (10 amino acids upstream and downstream of the site, but phosphorylation with modify-13-mers that 6 amino acids upstream and downstream of the site) in all protein sequences. Only when the minimum number of occurrences was set to 20 and the statistical test P value is less than 0.000001, the characteristic sequence form is considered to be a motif of the modified peptide. Emulate original motif-x was ticked, and other parameters with default.

## Supplementary Material

Supplementary Figures

Supplementary Table 1

Supplementary Tables 2 and 3
