# Supplementary material for: Integrated proteome and phosphoproteome analyses of peripheral blood mononuclear cells in primary Sjögren syndrome patients
Source: Aging (Albany NY). 2020 Dec 3;13(1):1071–95. doi: 10.18632/aging.202233 (PMC7835054; doi:10.18632/aging.202233)
Supplement: Supplementary Figures [file aging-13-202233-s001.pdf]

SUPPLEMENTARY FIGURES

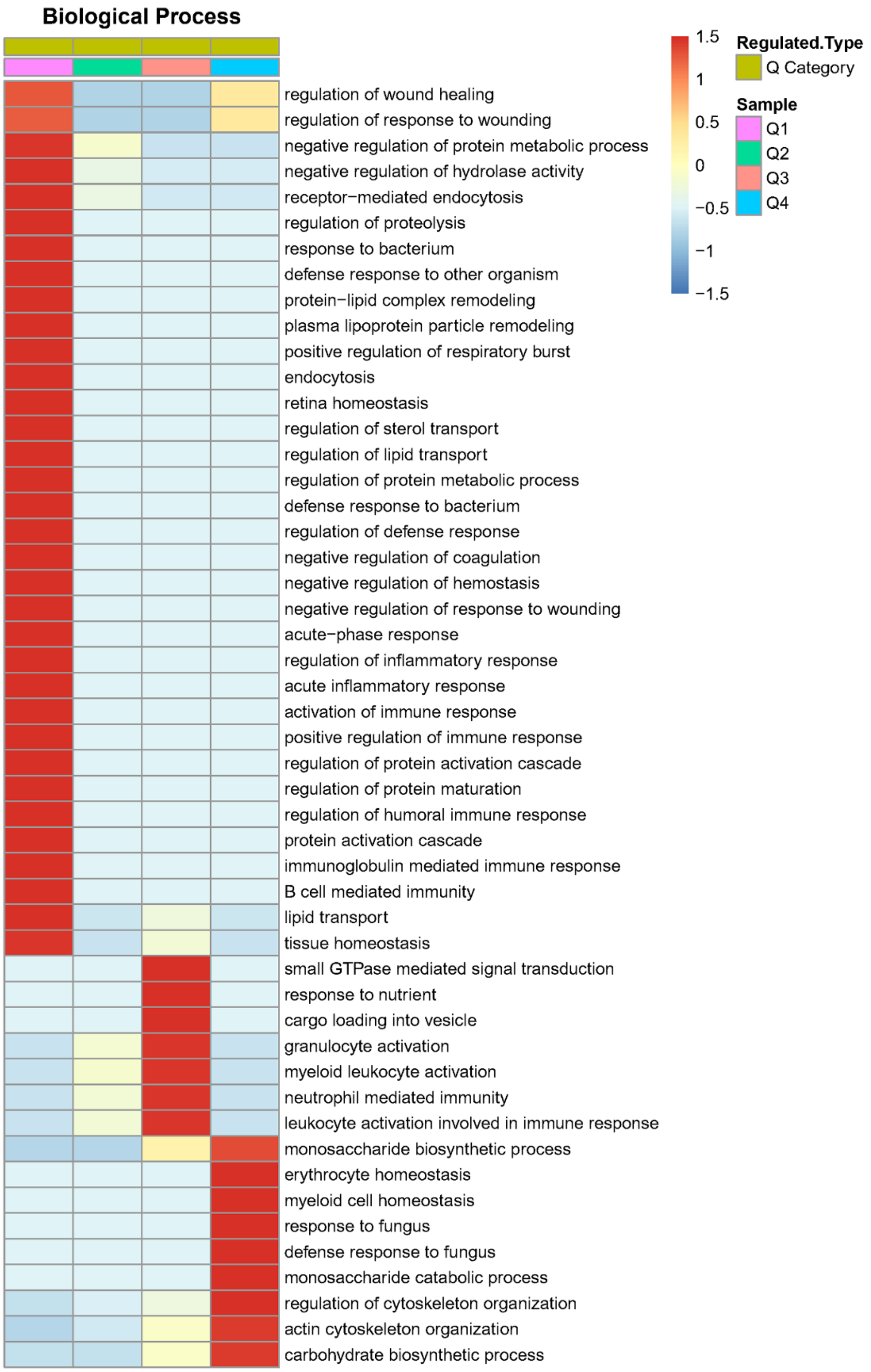

Supplementary Figure 1. Functional enrichment clustering analyses of differentially expressed proteins (DEPs) in four quantiles (Q1-Q4) based on biological process.

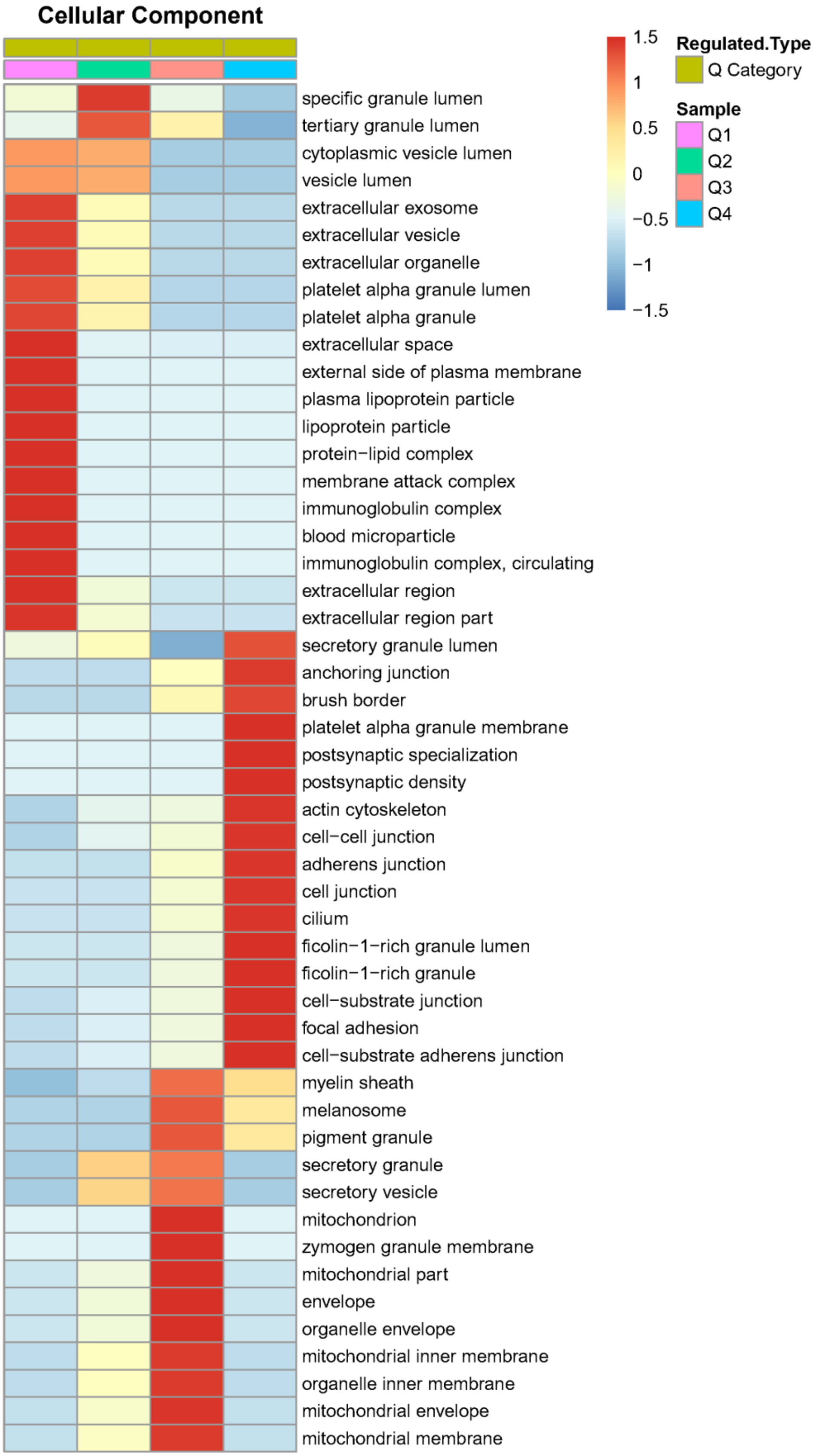

Supplementary Figure 2. Functional enrichment clustering analyses of differentially expressed proteins (DEPs) in four quantiles (Q1-Q4) based on cellular component.

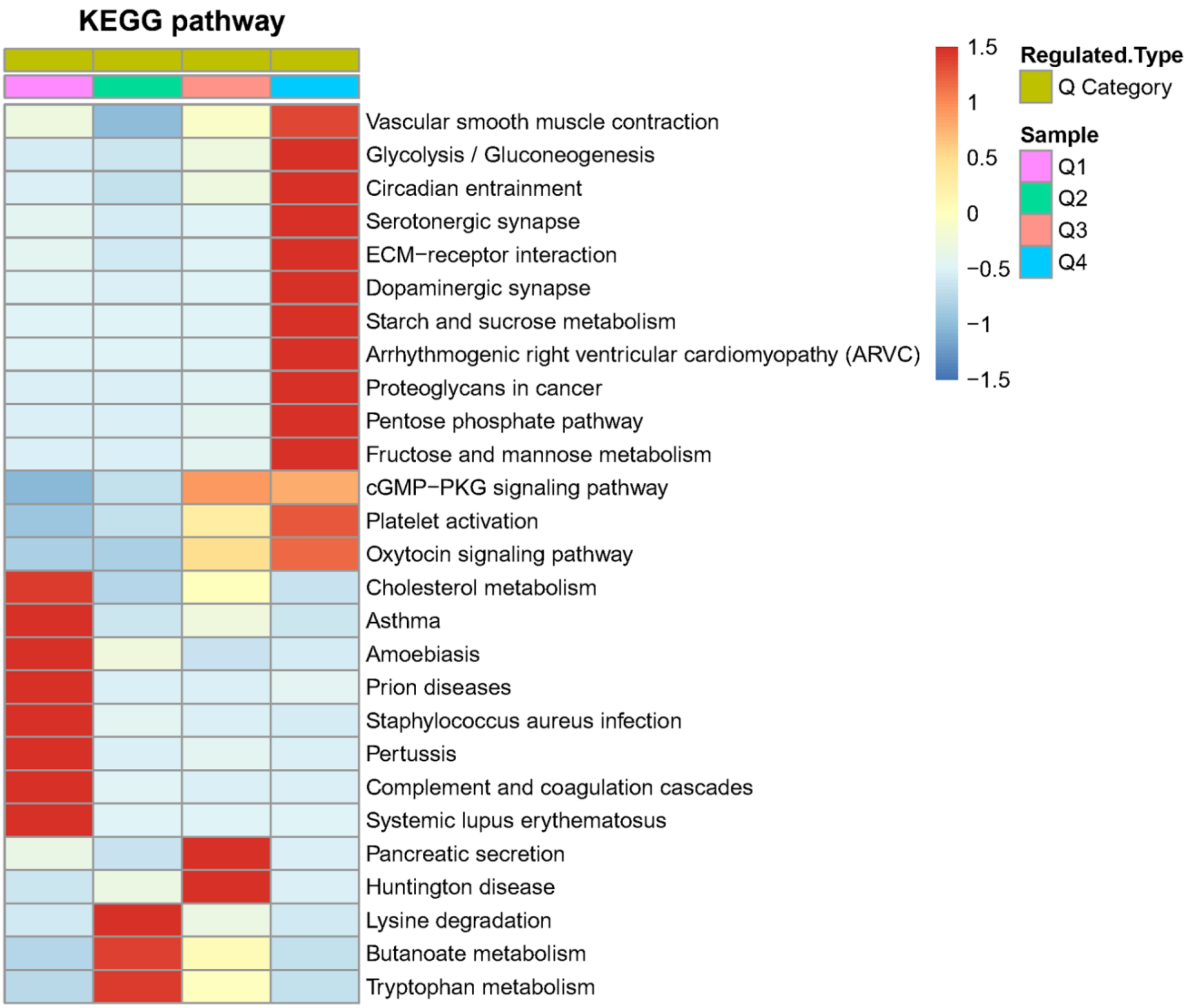

Supplementary Figure 3. Kyoto Encyclopedia of Genes and Genomes (KEGG) enrichment clustering analyses of differentially expressed proteins (DEPs) in four quantiles (Q1-Q4).

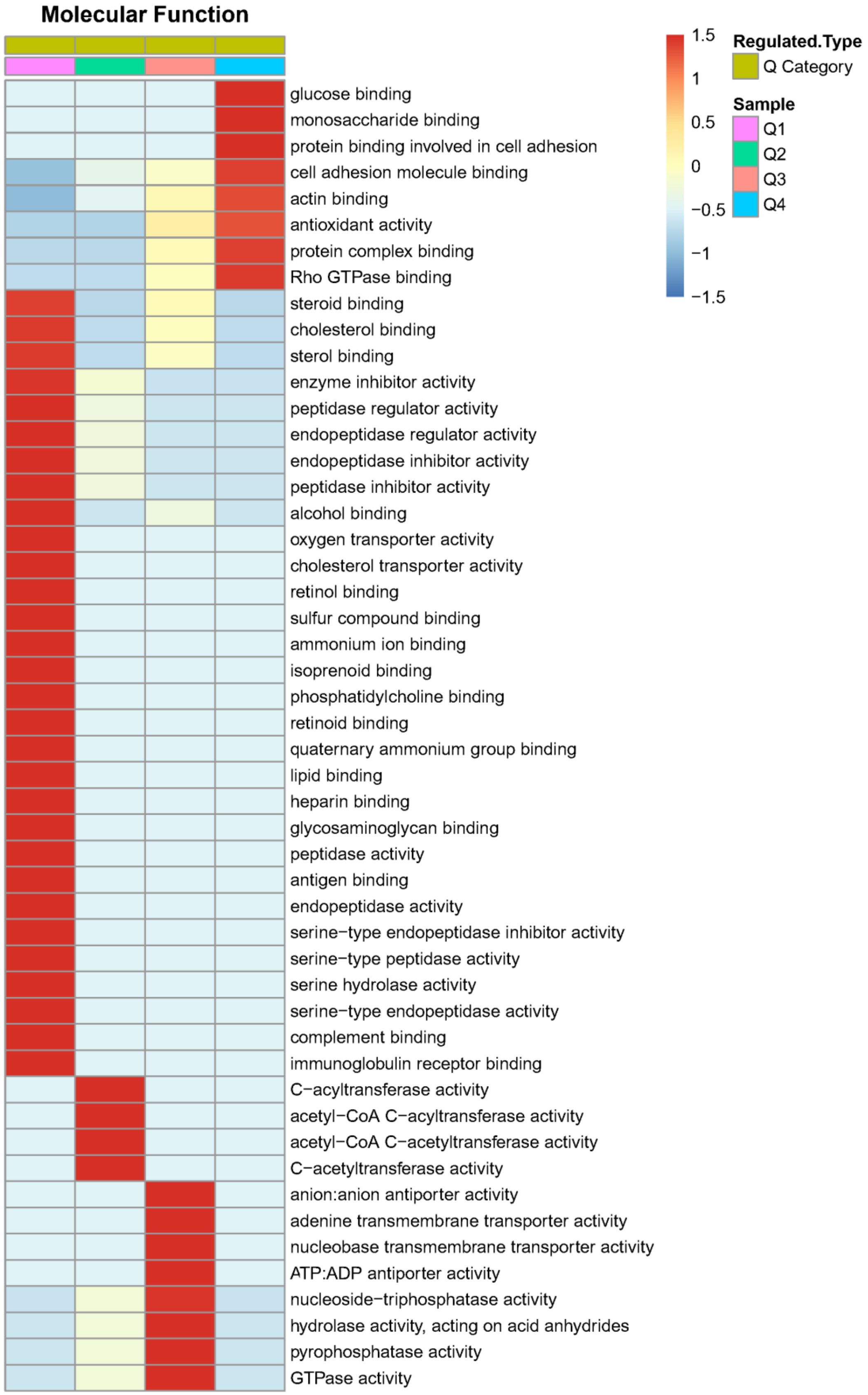

Supplementary Figure 4. Functional enrichment clustering analyses of differentially expressed proteins (DEPs) in four quantiles (Q1-Q4) based on molecular function.

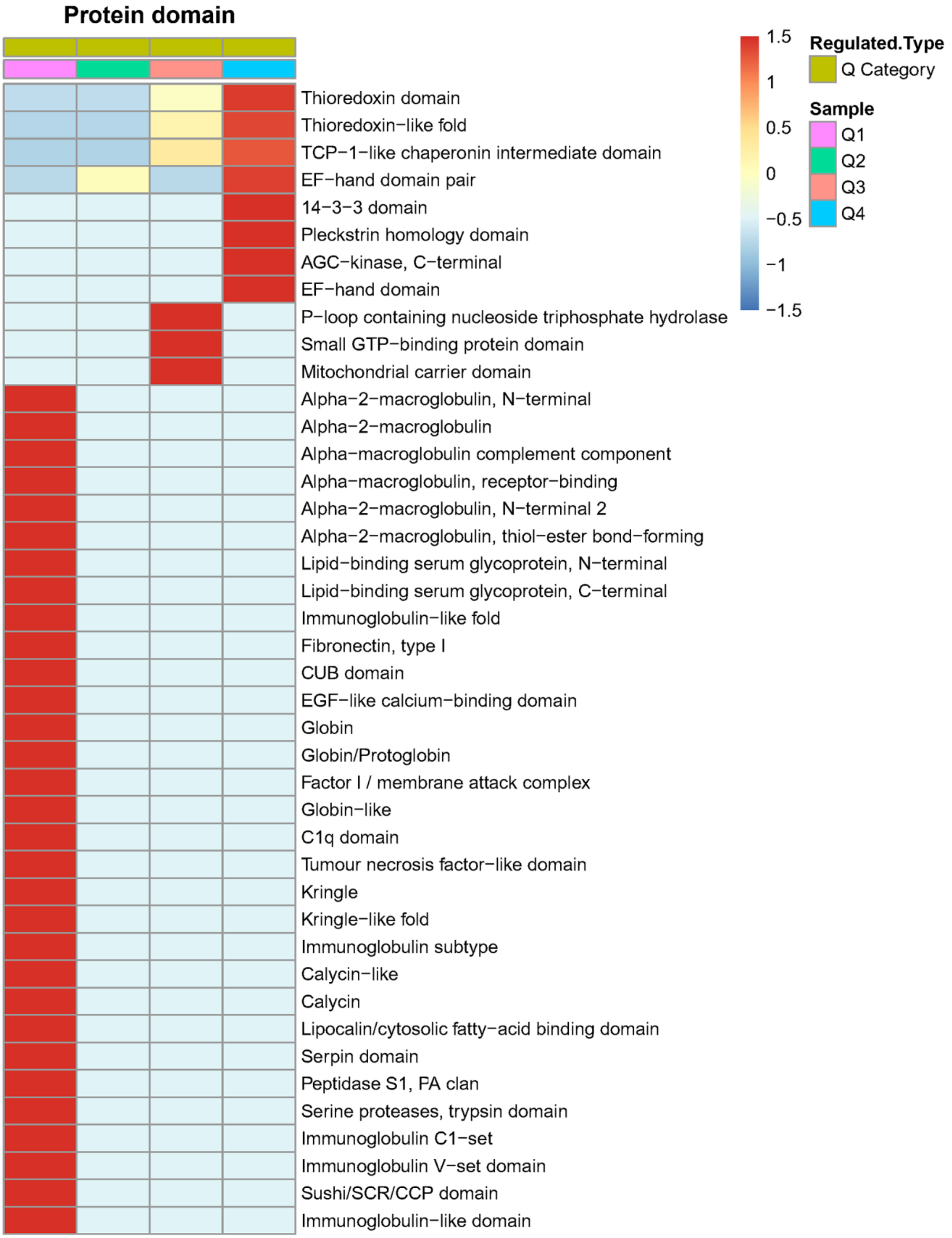

Supplementary Figure 5. Functional enrichment clustering analyses of differentially expressed proteins (DEPs) in four quantiles (Q1-Q4) based on protein domain.

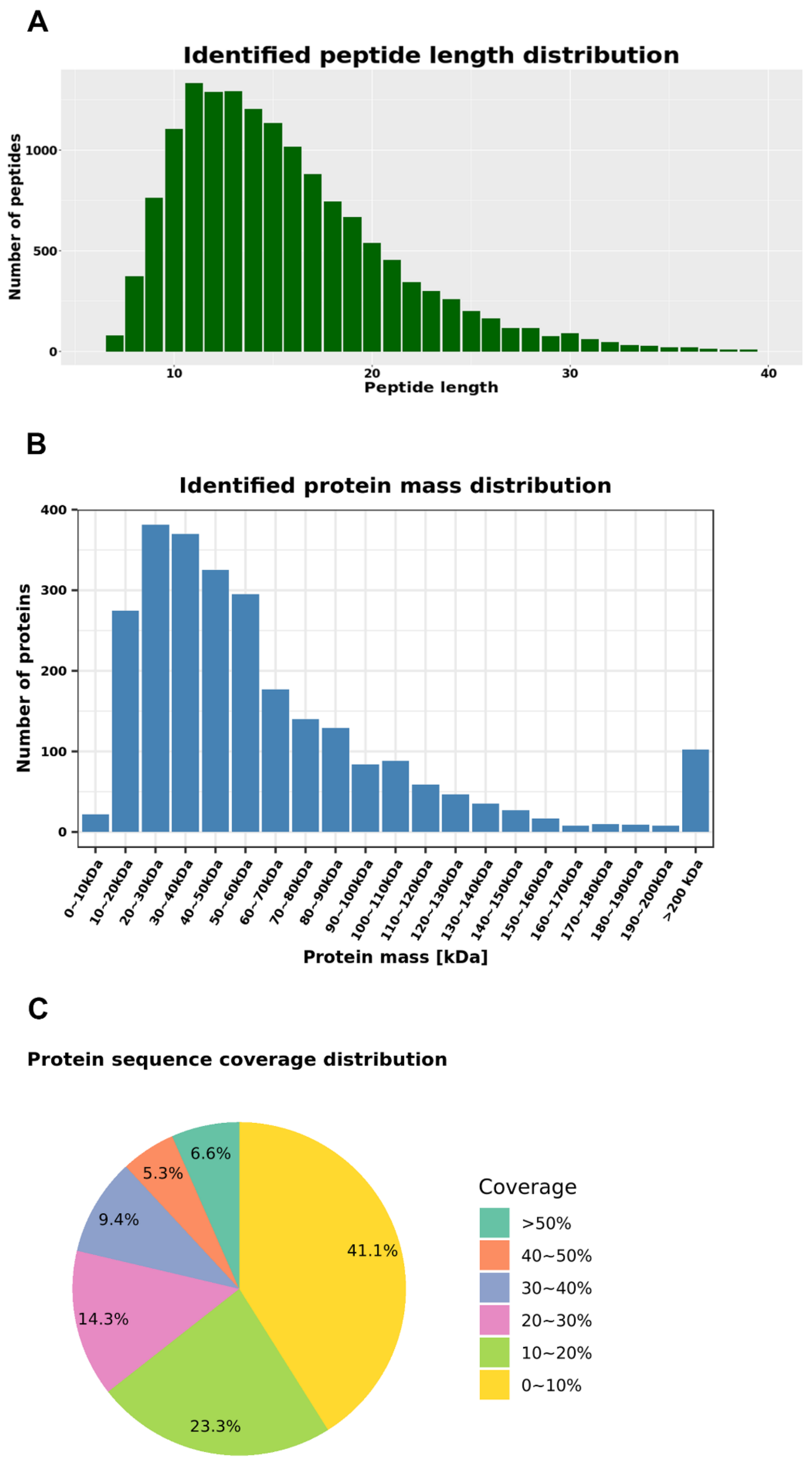

Supplementary Figure 6. (**A**) The length distribution of peptides identified by mass spectrometry. (**B**) Molecular weight distribution of all identified proteins. (**C**) Protein sequence coverage distribution.

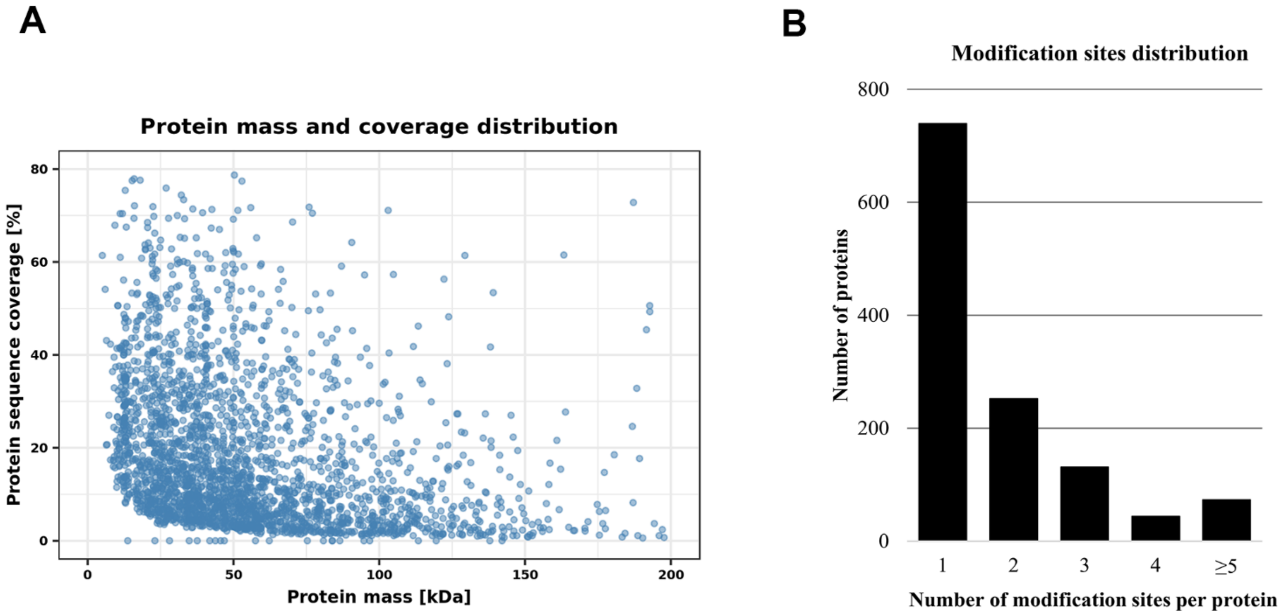

Supplementary Figure 7. (**A**) The relationship between the molecular weight of the protein and the coverage determined by mass spectrometry. (**B**) The number of modification sites of each corresponding protein.
