# Supplementary material for: Integrated proteome and phosphoproteome analyses of peripheral blood mononuclear cells in primary Sjögren syndrome patients
Source: Aging (Albany NY). 2020 Dec 3;13(1):1071–95. doi: 10.18632/aging.202233 (PMC7835054; doi:10.18632/aging.202233)
Supplement: Supplementary Table 1 [file aging-13-202233-s002.docx]

**Supplementary Table 1. All identified and quantified proteins together with quantitative information.**

| **Gene name** | **Mol. weight [kDa]** | **PSS** | **N1** | **PSS/N1 Ratio** |
| --- | --- | --- | --- | --- |
| IGLV4-69 | 12.773 |  | 1 |  |
| IGLV2-18 | 12.412 |  |  |  |
| IGLV3-10 | 12.441 | 0.01 | 1.99 | 0.005 |
| IGLV3-9 | 12.332 | 0.409 | 1.591 | 0.257 |
| IGHV3-64 | 12.891 |  |  |  |
| IGHV4-4 | 12.848 |  | 1 |  |
| IGKV2D-29 | 13.143 |  | 1 |  |
| IGKV1-27 | 12.711 |  | 1 |  |
| IGKV2D-30 | 13.215 |  | 1 |  |
| IGLV5-45 | 13.162 |  | 1 |  |
| IGKV3D-15 | 12.534 | 0.009 | 1.991 | 0.005 |
| IGKV1D-8 | 12.837 |  | 1 |  |
| IGKV2-40 | 13.31 |  | 1 |  |
| IGKV3D-11 | 12.625 | 0.005 | 1.995 | 0.003 |
| IGHV1-45 | 13.508 |  | 1 |  |
| IGHV3-49 | 13.056 |  |  |  |
| IGKV6D-21 | 12.34 |  | 1 |  |
| IGLV1-36 | 12.478 |  | 1 |  |
| IGHV3-15 | 12.926 |  |  |  |
| IGHV2-26 | 13.182 |  | 1 |  |
| IGHV3-73 | 12.858 |  |  |  |
| IGHV3-74 | 12.839 |  |  |  |
| IGHV3-43 | 13.077 |  | 1 |  |
| IGLV9-49 | 13.024 |  | 1 |  |
| IGHV3-72 | 13.203 |  |  |  |
| IGHV1-69D | 12.66 |  | 1 |  |
| IGKV3D-20 | 12.515 |  | 1 |  |
| IGHV1-3 | 13.008 |  |  |  |
| IGHV1-18 | 12.82 |  |  |  |
| IGHV3-20 | 12.673 |  | 1 |  |
| IGHV1-24 | 12.824 |  | 1 |  |
| IGHV4-28 | 13.124 |  |  |  |
| IGHV5-51 | 12.674 | 0.02 | 1.98 | 0.01 |
| IGKV1-8 | 12.537 | 0.007 | 1.993 | 0.004 |
| IGKV2-24 | 13.079 | 0.021 | 1.979 | 0.011 |
| IGKV1-6 | 12.697 |  |  |  |
| IGKV1-12 | 12.645 |  |  |  |
| IGHV3-64D | 12.822 |  |  |  |
| UBA6 | 117.97 | 1.786 | 0.214 | 8.346 |
| ESYT2 | 102.36 | 1.746 | 0.254 | 6.874 |
| IGLC7 | 11.253 |  | 1 |  |
| ILVBL | 67.867 |  |  |  |
| IGKV2-29 | 13.085 | 0.023 | 1.977 | 0.012 |
| NBAS | 268.57 | 1 |  |  |
| TARSL2 | 92.644 | 1 |  |  |
| TESPA1 | 59.213 |  |  |  |
| DENND3 | 135.89 |  |  |  |
| GSAP | 97.8 | 1 |  |  |
| WDR91 | 83.343 |  |  |  |
| CNOT1 | 266.94 | 1 |  |  |
| PGP | 34.006 | 1 |  |  |
| MFSD2B | 53.742 |  |  |  |
| GGT3P | 61.501 |  |  |  |
| TUBAL3 | 49.908 |  |  |  |
| NCF1C | 41.85 | 1.664 | 0.336 | 4.952 |
| MIF4GD | 25.423 |  |  |  |
| MYO1G | 116.44 | 0.655 | 1.345 | 0.487 |
| SLC35A4 | 11.133 | 1 |  |  |
| AGPS | 72.911 | 1.588 | 0.412 | 3.854 |
| KIF2A | 79.954 | 1.591 | 0.409 | 3.89 |
| PDLIM1 | 36.071 | 1.486 | 0.514 | 2.891 |
| ACOT7 | 41.796 | 1.579 | 0.421 | 3.751 |
| MYO1C | 121.68 |  |  |  |
| MYO1F | 124.84 |  |  |  |
| SNAP23 | 23.354 |  |  |  |
| AIP | 37.636 | 1 |  |  |
| LGALS9 | 39.518 |  |  |  |
| STXBP3 | 67.764 |  |  |  |
| RAB27B | 24.608 | 1.24 | 0.76 | 1.632 |
| AP3B1 | 121.32 | 0.943 | 1.057 | 0.892 |
| LGALS8 | 35.808 | 1 |  |  |
| PSMD11 | 47.463 |  |  |  |
| PSMD12 | 52.904 | 1.725 | 0.275 | 6.273 |
| PGRMC1 | 21.671 | 1.338 | 0.662 | 2.021 |
| CLIC1 | 26.922 | 1.584 | 0.416 | 3.808 |
| PIK3CD | 119.48 | 1 |  |  |
| QSOX1 | 82.577 | 0.753 | 1.247 | 0.604 |
| IPO5 | 123.63 | 1.516 | 0.484 | 3.132 |
| DNM1L | 81.876 | 1.38 | 0.62 | 2.226 |
| EXOC5 | 81.852 | 1 |  |  |
| BTN3A3 | 65.001 |  |  |  |
| NDUFA4 | 9.3697 | 1.104 | 0.896 | 1.232 |
| CLDN5 | 23.147 | 1 |  |  |
| KPNA3 | 57.81 | 1 |  |  |
| VWA5A | 86.488 | 1.735 | 0.265 | 6.547 |
| DDX3X | 73.243 | 1.808 | 0.192 | 9.417 |
| FCN1 | 35.078 | 1.124 | 0.876 | 1.283 |
| KPNA4 | 57.886 | 1 |  |  |
| PPP6C | 35.144 | 1.488 | 0.512 | 2.906 |
| MAN2B1 | 113.74 | 0.955 | 1.045 | 0.914 |
| PDXK | 35.102 | 1 |  |  |
| C2CD2L | 76.18 | 1 |  |  |
| COX7A2L | 12.615 | 1 |  |  |
| COPE | 34.482 |  |  |  |
| AP3D1 | 130.16 | 1.534 | 0.466 | 3.292 |
| CCS | 29.04 | 1.359 | 0.641 | 2.12 |
| ABLIM1 | 87.687 | 1 |  |  |
| STX16 | 37.031 |  |  |  |
| ADAM10 | 84.141 | 1.529 | 0.471 | 3.246 |
| APAF1 | 141.84 |  |  |  |
| ACOT8 | 35.914 | 1 |  |  |
| CDIPT | 23.539 | 1 |  |  |
| PRMT5 | 72.683 | 1 |  |  |
| SLC9A3R1 | 38.868 | 0.6 | 1.4 | 0.429 |
| TPP1 | 61.247 | 1.255 | 0.745 | 1.685 |
| GNB5 | 43.566 | 1 |  |  |
| TNPO2 | 101.39 | 1 |  |  |
| APOL1 | 43.974 |  | 1 |  |
| PSMA7 | 27.887 | 1.515 | 0.485 | 3.124 |
| OPLAH | 137.46 | 1 |  |  |
| BCKDK | 46.36 | 1 |  |  |
| IFIT3 | 55.984 | 1 |  |  |
| MGST3 | 16.516 | 1.543 | 0.457 | 3.376 |
| IKBKB | 86.563 |  |  |  |
| HAT1 | 49.512 | 1 |  |  |
| UBE2L6 | 17.769 | 1 |  |  |
| HGS | 86.191 | 1 |  |  |
| VPS26C | 33.01 |  |  |  |
| XPO1 | 123.38 | 1.391 | 0.609 | 2.284 |
| MAST4 | 284.09 |  |  |  |
| SEC16A | 251.89 |  |  |  |
| PLXNB2 | 205.12 |  |  |  |
| PFAS | 144.73 |  |  |  |
| ANKRD28 | 112.96 | 1 |  |  |
| CHUK | 84.639 | 1 |  |  |
| FYB1 | 85.386 | 1.799 | 0.201 | 8.95 |
| SCAMP2 | 36.648 | 0.607 | 1.393 | 0.436 |
| ARPC1B | 40.949 | 1.455 | 0.545 | 2.67 |
| ARPC2 | 34.333 | 1.078 | 0.922 | 1.169 |
| ARPC3 | 20.546 | 1.421 | 0.579 | 2.454 |
| PLSCR1 | 35.049 |  |  |  |
| PGRMC2 | 23.818 | 1.57 | 0.43 | 3.651 |
| CTDSPL | 31.128 |  |  |  |
| LAMA5 | 399.73 | 1 |  |  |
| ZNF185 | 73.525 | 1.125 | 0.875 | 1.286 |
| LEPROT | 14.254 | 1 |  |  |
| RER1 | 22.958 |  |  |  |
| SURF4 | 30.394 | 1.59 | 0.41 | 3.878 |
| SPTLC2 | 62.924 | 1 |  |  |
| OGT | 116.92 | 1 |  |  |
| PMM2 | 28.082 |  |  |  |
| INPP4B | 104.74 | 1.75 | 0.25 | 7 |
| EIF3D | 63.972 |  |  |  |
| EIF3H | 39.93 | 1 |  |  |
| BCAT2 | 44.287 | 1 |  |  |
| ABCC4 | 149.52 | 1 |  |  |
| YKT6 | 22.417 | 1 |  |  |
| ARPC5 | 16.32 | 1.429 | 0.571 | 2.503 |
| TAPBP | 47.625 | 1.659 | 0.341 | 4.865 |
| CLDN3 | 23.318 |  |  |  |
| DHX15 | 90.932 | 1.372 | 0.628 | 2.185 |
| ZZEF1 | 331.07 | 1.906 | 0.094 | 20.277 |
| ASAP2 | 111.65 | 1.955 | 0.045 | 43.444 |
| CYB5B | 16.694 | 0.939 | 1.061 | 0.885 |
| ARHGAP6 | 105.95 | 1.489 | 0.511 | 2.914 |
| DYNC1LI2 | 54.099 |  |  |  |
| PSMD3 | 60.977 |  |  |  |
| TGFB1I1 | 49.814 |  |  |  |
| SEC14L5 | 78.941 | 1 |  |  |
| ADCY6 | 130.61 |  |  |  |
| HNRNPR | 70.942 | 1.467 | 0.533 | 2.752 |
| TXNL1 | 32.251 | 1.721 | 0.279 | 6.168 |
| EIF4G3 | 176.65 |  |  |  |
| AKR7A2 | 39.589 | 1.562 | 0.438 | 3.566 |
| TGOLN2 | 51.018 | 1.078 | 0.922 | 1.169 |
| LAMTOR5 | 9.6138 | 0.826 | 1.174 | 0.704 |
| XPOT | 109.96 |  |  |  |
| TIMM44 | 51.355 | 1 |  |  |
| NCK2 | 42.915 | 1.352 | 0.648 | 2.086 |
| PLRG1 | 57.193 | 1 |  |  |
| RGS10 | 21.21 | 1.789 | 0.211 | 8.479 |
| ASNA1 | 38.792 | 1.311 | 0.689 | 1.903 |
| BUB3 | 37.154 |  |  |  |
| ACTN4 | 104.85 | 1.462 | 0.538 | 2.717 |
| GSTZ1 | 24.212 | 1 |  |  |
| AP1G1 | 91.35 | 1.345 | 0.655 | 2.053 |
| SYNGR2 | 24.81 |  |  |  |
| SLC25A20 | 32.943 |  |  |  |
| NARS | 62.942 | 1.794 | 0.206 | 8.709 |
| LANCL1 | 45.283 | 1.69 | 0.31 | 5.452 |
| STRN | 86.131 | 1.625 | 0.375 | 4.333 |
| IDH3B | 42.183 | 1 |  |  |
| NRDC | 131.7 | 1 |  |  |
| CALU | 37.106 | 1.613 | 0.387 | 4.168 |
| AHCYL1 | 58.951 |  |  |  |
| CD5L | 38.087 | 0.032 | 1.968 | 0.016 |
| KALRN | 340.17 | 1.52 | 0.48 | 3.167 |
| GMFG | 16.801 |  |  |  |
| PRPSAP2 | 40.925 | 1.646 | 0.354 | 4.65 |
| SMARCA5 | 121.9 | 1 |  |  |
| SPAG9 | 146.2 | 0.7 | 1.3 | 0.538 |
| OPA1 | 111.63 | 1 |  |  |
| SNX3 | 18.762 | 1 |  |  |
| DOK2 | 45.378 | 1.747 | 0.253 | 6.905 |
| SYNCRIP | 69.602 |  |  |  |
| GMDS | 41.949 | 1 |  |  |
| DIAPH1 | 141.35 | 1.461 | 0.539 | 2.711 |
| SELENOF | 18.092 | 1 |  |  |
| TSPAN2 | 24.147 | 1 |  |  |
| EXOC3 | 85.566 | 1 |  |  |
| PLIN3 | 47.074 | 1 |  |  |
| SLC16A7 | 52.2 | 1 |  |  |
| TPST2 | 41.911 | 1 |  |  |
| SNX2 | 58.47 |  |  |  |
| USO1 | 107.89 |  |  |  |
| PRAF2 | 19.258 |  |  |  |
| EIF5B | 138.83 |  |  |  |
| DNAJA2 | 45.745 | 1 |  |  |
| CUTA | 19.116 | 1.395 | 0.605 | 2.306 |
| PPP1R11 | 13.952 | 1 |  |  |
| FCGR3B | 26.216 |  |  |  |
| TMCC2 | 77.448 | 1 |  |  |
| WDR1 | 66.193 | 1.255 | 0.745 | 1.685 |
| ROCK2 | 160.9 | 1.517 | 0.483 | 3.141 |
| CPNE3 | 60.13 | 1.301 | 0.699 | 1.861 |
| DNAJC13 | 254.41 | 1.771 | 0.229 | 7.734 |
| PPP6R2 | 104.94 | 1 |  |  |
| GGCT | 21.007 |  |  |  |
| RTN2 | 59.263 | 1 |  |  |
| NDUFS2 | 52.545 |  |  |  |
| NIPSNAP2 | 33.742 |  |  |  |
| PDCD6 | 21.868 | 1.639 | 0.361 | 4.54 |
| TBCA | 12.855 |  |  |  |
| ATP6V1G1 | 13.757 |  |  |  |
| VPS4B | 49.301 | 1 |  |  |
| MPDU1 | 26.638 |  |  |  |
| H2AFY | 39.617 | 0.464 | 1.536 | 0.302 |
| SH3BGRL | 12.774 | 1.678 | 0.322 | 5.211 |
| FLNB | 278.16 | 1.672 | 0.328 | 5.098 |
| NDUFS6 | 13.711 | 1.296 | 0.704 | 1.841 |
| CS | 51.712 | 1.184 | 0.816 | 1.451 |
| SEC22B | 24.593 | 1.608 | 0.392 | 4.102 |
| MTX2 | 29.763 |  |  |  |
| VPS26A | 38.169 |  |  |  |
| ERN1 | 109.73 |  | 1 |  |
| NDUFS3 | 30.241 | 0.952 | 1.048 | 0.908 |
| SRSF10 | 31.3 | 1 |  |  |
| SF3B1 | 145.83 |  |  |  |
| CSDE1 | 88.884 | 1 |  |  |
| STX11 | 33.195 | 1.125 | 0.875 | 1.286 |
| SKAP2 | 41.216 | 1.426 | 0.574 | 2.484 |
| MYCBP2 | 513.63 | 1 |  |  |
| PGLYRP1 | 21.731 | 1.282 | 0.718 | 1.786 |
| LYPLA1 | 24.669 | 1.725 | 0.275 | 6.273 |
| FCN3 | 32.903 |  |  |  |
| TIPRL | 31.444 | 1.566 | 0.434 | 3.608 |
| PPM1B | 52.642 |  |  |  |
| RP2 | 39.641 | 1.675 | 0.325 | 5.154 |
| CRTAP | 46.561 | 1 |  |  |
| SLC25A12 | 74.761 | 1 |  |  |
| GRAP2 | 37.909 | 1.863 | 0.137 | 13.599 |
| ZMPSTE24 | 54.812 |  |  |  |
| IDH1 | 46.659 | 1.568 | 0.432 | 3.63 |
| ATRN | 158.54 | 0.009 | 1.991 | 0.005 |
| ARL6IP5 | 21.614 | 1.519 | 0.481 | 3.158 |
| DNAJC8 | 29.841 | 1 |  |  |
| ATP5PD | 18.491 | 1.447 | 0.553 | 2.617 |
| TSPAN9 | 26.779 | 1.804 | 0.196 | 9.204 |
| FLOT1 | 47.355 | 0.847 | 1.153 | 0.735 |
| ATP5MG | 11.428 | 1.092 | 0.908 | 1.203 |
| SASH3 | 41.595 | 1 |  |  |
| GLRX3 | 37.432 | 1 |  |  |
| CIAO1 | 37.84 |  |  |  |
| PDE5A | 99.984 | 1.597 | 0.403 | 3.963 |
| B3GAT3 | 37.121 |  |  |  |
| MTA2 | 75.022 |  |  |  |
| STK10 | 112.13 |  |  |  |
| TOMM70 | 67.454 | 1.592 | 0.408 | 3.902 |
| SEC24D | 113.01 | 1 |  |  |
| UFL1 | 89.594 | 1 |  |  |
| TMEM63A | 92.125 |  |  |  |
| PLPBP | 30.344 |  |  |  |
| ERLIN2 | 37.839 |  |  |  |
| ENDOD1 | 55.016 | 1.471 | 0.529 | 2.781 |
| GLS | 73.46 | 1.104 | 0.896 | 1.232 |
| AP2A2 | 103.96 |  |  |  |
| SEC31A | 133.01 | 1.16 | 0.84 | 1.381 |
| MFN2 | 86.401 |  |  |  |
| UBE4B | 146.18 | 1 |  |  |
| ELP1 | 150.25 | 1.388 | 0.612 | 2.268 |
| NDUFB8 | 21.766 |  |  |  |
| RTN3 | 112.61 | 0.496 | 1.504 | 0.33 |
| LETM1 | 83.353 | 1 |  |  |
| SNX4 | 51.908 | 1 |  |  |
| ATE1 | 59.09 | 1.612 | 0.388 | 4.155 |
| VAPB | 27.228 | 1 |  |  |
| NDUFC2 | 14.187 |  |  |  |
| NDUFA10 | 40.75 |  |  |  |
| CELF2 | 54.284 |  |  |  |
| PGLS | 27.547 | 1 |  |  |
| ATG7 | 77.959 | 1 |  |  |
| LYPLA2 | 24.737 |  |  |  |
| IPO7 | 119.52 | 1.843 | 0.157 | 11.739 |
| PIGN | 105.81 | 1 |  |  |
| AHSA1 | 38.274 | 1.576 | 0.424 | 3.717 |
| APOM | 21.253 |  |  |  |
| FMNL1 | 121.85 | 1 |  |  |
| VNN1 | 57.011 |  |  |  |
| VNN2 | 58.502 | 1 |  |  |
| SFT2D2 | 17.779 |  |  |  |
| MPC2 | 14.279 |  |  |  |
| ETHE1 | 27.873 | 1.298 | 0.702 | 1.849 |
| ACSL3 | 80.419 |  |  |  |
| STAMBP | 48.076 | 1.774 | 0.226 | 7.85 |
| ASMTL | 68.856 | 1.783 | 0.217 | 8.217 |
| CDS2 | 51.417 | 1.623 | 0.377 | 4.305 |
| RAB3D | 24.267 |  |  |  |
| OXSR1 | 58.022 |  |  |  |
| AP2A1 | 107.54 | 1.721 | 0.279 | 6.168 |
| CAVIN2 | 47.173 | 0.965 | 1.035 | 0.932 |
| MAP4K4 | 142.1 |  |  |  |
| MLYCD | 55.003 |  |  |  |
| CRYZL1 | 38.696 |  |  |  |
| AIFM1 | 66.9 |  |  |  |
| EML2 | 70.678 | 1.462 | 0.538 | 2.717 |
| GNA14 | 41.57 |  |  |  |
| TSPAN15 | 33.165 |  |  |  |
| DDAH2 | 29.644 |  |  |  |
| MPIG6B | 26.163 | 1.633 | 0.367 | 4.45 |
| ABHD16A | 63.243 | 1.392 | 0.608 | 2.289 |
| RECK | 106.46 | 1 |  |  |
| NUDT3 | 19.471 |  |  |  |
| TOMM40 | 37.893 | 1.392 | 0.608 | 2.289 |
| PEX11B | 28.431 | 1.756 | 0.244 | 7.197 |
| LDHA | 36.688 | 1.265 | 0.735 | 1.721 |
| ALDH1A1 | 54.861 | 1.604 | 0.396 | 4.051 |
| GLUD1 | 61.397 | 1.152 | 0.848 | 1.358 |
| CYB5R3 | 34.234 | 1.457 | 0.543 | 2.683 |
| GSR | 56.256 | 1.631 | 0.369 | 4.42 |
| MT-CO1 | 57.041 | 1 |  |  |
| MT-CO2 | 25.565 | 1.002 | 0.998 | 1.004 |
| MT-CO3 | 29.95 |  |  |  |
| SOD1 | 15.936 | 1.632 | 0.368 | 4.435 |
| F13A1 | 83.266 | 1.348 | 0.652 | 2.067 |
| PNP | 32.118 | 1.414 | 0.586 | 2.413 |
| HPRT1 | 24.579 | 1.194 | 0.806 | 1.481 |
| GOT2 | 47.517 | 1.324 | 0.676 | 1.959 |
| PGK1 | 44.614 | 1.188 | 0.812 | 1.463 |
| AK1 | 21.635 | 0.919 | 1.081 | 0.85 |
| C1R | 80.118 | 0.05 | 1.95 | 0.026 |
| HP | 45.205 | 0.008 | 1.992 | 0.004 |
| HPR | 39.029 |  | 1 |  |
| F9 | 51.778 |  | 1 |  |
| F10 | 54.731 |  |  |  |
| CFD | 27.033 |  |  |  |
| F12 | 67.791 | 0.025 | 1.975 | 0.013 |
| CFB | 85.532 | 0.004 | 1.996 | 0.002 |
| CA1 | 28.87 | 0.142 | 1.858 | 0.076 |
| CA2 | 29.246 | 1.072 | 0.928 | 1.155 |
| SERPINA1 | 46.736 | 0.024 | 1.976 | 0.012 |
| SERPINA3 | 47.65 | 0.028 | 1.972 | 0.014 |
| AGT | 53.154 | 0.012 | 1.988 | 0.006 |
| A2M | 163.29 | 0.004 | 1.996 | 0.002 |
| TIMP1 | 23.171 | 0.778 | 1.222 | 0.637 |
| CSTA | 11.006 | 1 |  |  |
| PDGFB | 27.283 |  |  |  |
| EGF | 133.99 | 1 |  |  |
| TGFB1 | 44.341 | 1.585 | 0.415 | 3.819 |
| JCHAIN | 18.098 | 0.025 | 1.975 | 0.013 |
| IGKV1-33 | 12.848 |  |  |  |
| IGKV1-17 | 12.778 |  | 1 |  |
| IGKV1D-16 | 12.73 |  | 1 |  |
| IGKV1-5 | 12.781 |  | 1 |  |
| IGKV3-20 | 12.557 | 0.018 | 1.982 | 0.009 |
| IGLV1-47 | 12.283 |  |  |  |
| IGLV1-40 | 12.301 |  |  |  |
| IGLV2-11 | 12.644 |  | 1 |  |
| IGLV3-19 | 12.042 | 0.007 | 1.993 | 0.004 |
| IGLV3-1 | 12.296 |  | 1 |  |
| IGLV3-25 | 12.011 |  |  |  |
| IGLV6-57 | 12.566 |  |  |  |
| IGHV1-46 | 12.933 |  |  |  |
| IGHV3-11 | 12.909 |  | 1 |  |
| IGHV3-23 | 12.582 |  | 1 |  |
| IGHV3-13 | 12.506 |  | 1 |  |
| IGHV3-33 | 13.074 | 0.146 | 1.854 | 0.079 |
| IGHV2-5 | 13.231 |  | 1 |  |
| PIGR | 83.283 |  |  |  |
| IGKC | 11.765 | 0.018 | 1.982 | 0.009 |
| IGHG2 | 35.9 | 0.011 | 1.989 | 0.006 |
| IGHG3 | 41.287 | 0.027 | 1.973 | 0.014 |
| IGHG4 | 35.94 | 0.013 | 1.987 | 0.007 |
| IGHM | 49.439 | 0.03 | 1.97 | 0.015 |
| IGHA1 | 37.654 | 0.028 | 1.972 | 0.014 |
| IGHA2 | 36.591 |  |  |  |
| HLA-A | 40.921 | 1.695 | 0.305 | 5.557 |
| HLA-DRA | 28.607 | 1.273 | 0.727 | 1.751 |
| HBD | 16.055 | 0.101 | 1.899 | 0.053 |
| COL1A1 | 138.94 | 1.368 | 0.632 | 2.165 |
| LMNA | 74.139 |  |  |  |
| APOA2 | 11.175 | 0.022 | 1.978 | 0.011 |
| APOC2 | 11.284 |  | 1 |  |
| APOC3 | 10.852 |  |  |  |
| FGG | 51.511 | 0.573 | 1.427 | 0.402 |
| SLC4A1 | 101.79 |  |  |  |
| APCS | 25.387 | 0.018 | 1.982 | 0.009 |
| C1QA | 26.016 | 0.313 | 1.687 | 0.186 |
| C1QB | 26.721 | 0.166 | 1.834 | 0.091 |
| C1QC | 25.773 | 0.258 | 1.742 | 0.148 |
| C9 | 63.173 |  |  |  |
| LRG1 | 38.177 |  |  |  |
| FN1 | 262.62 | 0.072 | 1.928 | 0.037 |
| RBP4 | 23.01 | 0.024 | 1.976 | 0.012 |
| ORM1 | 23.511 | 0.037 | 1.963 | 0.019 |
| TTR | 15.887 | 0.042 | 1.958 | 0.021 |
| PPBP | 13.894 | 1.61 | 0.39 | 4.128 |
| PF4 | 10.845 | 0.463 | 1.537 | 0.301 |
| TFRC | 84.87 |  | 1 |  |
| TF | 77.063 | 0.008 | 1.992 | 0.004 |
| LTF | 78.181 | 1.347 | 0.653 | 2.063 |
| HPX | 51.676 | 0.012 | 1.988 | 0.006 |
| FTL | 20.019 | 1.755 | 0.245 | 7.163 |
| FTH1 | 21.225 |  |  |  |
| F11 | 70.108 |  | 1 |  |
| KLKB1 | 71.369 | 0.005 | 1.995 | 0.003 |
| C4BPA | 67.033 | 0.042 | 1.958 | 0.021 |
| CAT | 59.755 | 1.201 | 0.799 | 1.503 |
| FUCA1 | 53.688 | 1 |  |  |
| ALDOA | 39.42 | 1.33 | 0.67 | 1.985 |
| CSTB | 11.139 | 1.285 | 0.715 | 1.797 |
| ANXA1 | 38.714 | 1.762 | 0.238 | 7.403 |
| PRNP | 27.661 | 1.577 | 0.423 | 3.728 |
| SOD2 | 24.75 | 1.708 | 0.292 | 5.849 |
| LCAT | 49.577 |  |  |  |
| HRG | 59.578 | 0.018 | 1.982 | 0.009 |
| A1BG | 54.253 | 0.015 | 1.985 | 0.008 |
| HLA-C | 40.86 | 1 |  |  |
| KRT6B | 60.066 | 0.534 | 1.466 | 0.364 |
| VWF | 309.26 | 1.494 | 0.506 | 2.953 |
| SHBG | 43.779 |  | 1 |  |
| GAPDH | 36.053 | 0.604 | 1.396 | 0.433 |
| ASL | 51.657 | 1.199 | 0.801 | 1.497 |
| IGKV1-16 | 12.618 |  |  |  |
| IGKV1D-39 | 12.737 |  |  |  |
| HLA-DPB1 | 29.159 | 1 |  |  |
| CAPNS1 | 28.315 | 0.897 | 1.103 | 0.813 |
| HSPB1 | 22.782 | 0.73 | 1.27 | 0.575 |
| CYBB | 65.335 | 1.268 | 0.732 | 1.732 |
| RPN1 | 68.569 | 1.324 | 0.676 | 1.959 |
| RPN2 | 69.283 | 1.162 | 0.838 | 1.387 |
| GNAI2 | 40.45 | 1.455 | 0.545 | 2.67 |
| ATP1A1 | 112.89 | 1.268 | 0.732 | 1.732 |
| APP | 86.942 | 1.648 | 0.352 | 4.682 |
| APOD | 21.275 | 0.007 | 1.993 | 0.004 |
| ALDH2 | 56.381 | 0.846 | 1.154 | 0.733 |
| ITGB3 | 87.057 | 1.433 | 0.567 | 2.527 |
| ITGB2 | 84.781 | 1.086 | 0.914 | 1.188 |
| S100A8 | 10.834 | 1.707 | 0.293 | 5.826 |
| SERPINE1 | 45.059 | 0.676 | 1.324 | 0.511 |
| SLC25A5 | 32.852 | 1.263 | 0.737 | 1.714 |
| SERPINA5 | 45.674 | 0.033 | 1.967 | 0.017 |
| SERPING1 | 55.154 | 0.01 | 1.99 | 0.005 |
| F13B | 75.51 | 0.063 | 1.937 | 0.033 |
| MPO | 83.868 | 1.562 | 0.438 | 3.566 |
| PCCA | 80.058 | 1 |  |  |
| PCCB | 58.215 | 1 |  |  |
| EIF2S1 | 36.112 |  |  |  |
| RPLP1 | 11.514 |  | 1 |  |
| SSB | 46.836 | 1 |  |  |
| HLA-A | 40.688 | 1.479 | 0.521 | 2.839 |
| SERPINA7 | 46.324 |  | 1 |  |
| ITGB1 | 88.414 | 1.87 | 0.13 | 14.385 |
| PRKCB | 76.868 | 1.648 | 0.352 | 4.682 |
| UROD | 40.786 |  |  |  |
| LCK | 58 | 1 |  |  |
| FYN | 60.761 | 1 |  |  |
| BCHE | 68.417 |  | 1 |  |
| GLA | 48.766 | 1 |  |  |
| IGKV4-1 | 13.38 | 0.008 | 1.992 | 0.004 |
| IGHV4-34 | 13.815 |  |  |  |
| PTMA | 12.203 | 1 |  |  |
| ATP5F1B | 56.559 | 0.923 | 1.077 | 0.857 |
| C2 | 83.267 | 0.02 | 1.98 | 0.01 |
| S100A9 | 13.242 | 1.729 | 0.271 | 6.38 |
| ENO1 | 47.168 | 1.384 | 0.616 | 2.247 |
| PYGL | 97.147 | 1.445 | 0.555 | 2.604 |
| GPI | 63.146 | 1.435 | 0.565 | 2.54 |
| NPM1 | 32.575 | 0.907 | 1.093 | 0.83 |
| TPM3 | 32.95 | 1.615 | 0.385 | 4.195 |
| ITGAV | 116.04 | 1.655 | 0.345 | 4.797 |
| HEXA | 60.702 | 1.062 | 0.938 | 1.132 |
| SERPINE2 | 44.002 | 1.537 | 0.463 | 3.32 |
| EPHX1 | 52.948 |  |  |  |
| DBI | 10.044 | 1 |  |  |
| FABP1 | 14.208 |  |  |  |
| LDHB | 36.638 | 1.393 | 0.607 | 2.295 |
| GPX1 | 22.088 | 1.057 | 0.943 | 1.121 |
| P4HB | 57.116 | 1.493 | 0.507 | 2.945 |
| FES | 93.495 | 1 |  |  |
| CTSD | 44.552 | 1.317 | 0.683 | 1.928 |
| ANXA2 | 38.604 | 0.513 | 1.487 | 0.345 |
| C8A | 65.163 |  |  |  |
| C8B | 67.046 | 0.063 | 1.937 | 0.033 |
| GP1BA | 71.539 | 1.484 | 0.516 | 2.876 |
| C8G | 22.277 | 0.006 | 1.994 | 0.003 |
| CAPN1 | 81.889 | 1.56 | 0.44 | 3.545 |
| TUBB | 49.67 | 0.655 | 1.345 | 0.487 |
| PSAP | 58.112 | 1 |  |  |
| HEXB | 63.111 | 1.302 | 0.698 | 1.865 |
| PFN1 | 15.054 | 1.261 | 0.739 | 1.706 |
| APRT | 19.608 | 1.019 | 0.981 | 1.039 |
| CD3E | 23.147 | 1.563 | 0.437 | 3.577 |
| EPRS | 170.59 |  |  |  |
| CTSB | 37.821 | 1.553 | 0.447 | 3.474 |
| HSP90AA1 | 84.659 | 1.26 | 0.74 | 1.703 |
| HNRNPC | 33.67 | 0.992 | 1.008 | 0.984 |
| UQCRH | 10.739 |  |  |  |
| YES1 | 60.801 | 1 |  |  |
| LYN | 58.573 | 1.399 | 0.601 | 2.328 |
| FH | 54.636 | 0.836 | 1.164 | 0.718 |
| COL1A2 | 129.31 | 1.47 | 0.53 | 2.774 |
| ANXA6 | 75.872 | 0.507 | 1.493 | 0.34 |
| SERPINA6 | 45.14 | 0.328 | 1.672 | 0.196 |
| SLC3A2 | 67.993 |  |  |  |
| GUSB | 74.731 | 1.643 | 0.357 | 4.602 |
| PFKM | 85.182 |  |  |  |
| HSP90AB1 | 83.263 | 1.548 | 0.452 | 3.425 |
| SRPRA | 69.81 |  |  |  |
| ELANE | 28.518 | 1.497 | 0.503 | 2.976 |
| SOD3 | 25.851 | 1 |  |  |
| CTSG | 28.837 | 1.716 | 0.284 | 6.042 |
| ITGA2B | 113.38 | 1.432 | 0.568 | 2.521 |
| LPA | 501.31 | 0.012 | 1.988 | 0.006 |
| PDHA1 | 43.295 |  |  |  |
| PLEK | 40.124 | 1.643 | 0.357 | 4.602 |
| CD14 | 40.076 | 0.25 | 1.75 | 0.143 |
| CYC1 | 35.422 | 1.392 | 0.608 | 2.289 |
| PTPRC | 147.48 | 1.03 | 0.97 | 1.062 |
| CFH | 139.09 | 0.025 | 1.975 | 0.013 |
| HCK | 59.599 | 1 |  |  |
| ITGA5 | 114.54 |  |  |  |
| GNAI3 | 40.532 | 1.531 | 0.469 | 3.264 |
| ANXA5 | 35.936 | 0.896 | 1.104 | 0.812 |
| RPSA | 32.854 |  |  |  |
| CD63 | 25.636 |  |  |  |
| SNRPA | 31.279 | 1 |  |  |
| ENO2 | 47.268 | 1.687 | 0.313 | 5.39 |
| ACAA1 | 44.292 |  |  |  |
| DBH | 69.064 |  | 1 |  |
| GSTP1 | 23.356 | 1.471 | 0.529 | 2.781 |
| SNRPC | 17.394 | 1 |  |  |
| VIL1 | 92.694 | 1 |  |  |
| LGALS1 | 14.716 | 1.221 | 0.779 | 1.567 |
| QDPR | 25.789 | 1.518 | 0.482 | 3.149 |
| HMGB1 | 24.893 | 1.346 | 0.654 | 2.058 |
| FBP1 | 36.842 | 1.42 | 0.58 | 2.448 |
| SPARC | 34.632 | 1.385 | 0.615 | 2.252 |
| GSTM1 | 25.712 | 1 |  |  |
| TPM1 | 32.708 | 1 |  |  |
| ANXA4 | 35.882 | 1.264 | 0.736 | 1.717 |
| CNP | 47.578 |  |  |  |
| DLD | 54.177 | 0.855 | 1.145 | 0.747 |
| HNRNPA1 | 38.746 | 1.503 | 0.497 | 3.024 |
| CTSH | 37.393 | 0.989 | 1.011 | 0.978 |
| COX6C | 8.7813 |  |  |  |
| FGR | 59.478 |  |  |  |
| C1S | 76.684 | 0.034 | 1.966 | 0.017 |
| PARP1 | 113.08 | 1 |  |  |
| ALOX5 | 77.982 | 1 |  |  |
| LTA4H | 69.284 | 1.892 | 0.108 | 17.519 |
| ALDOC | 39.455 | 1.415 | 0.585 | 2.419 |
| C4B | 192.75 | 0.006 | 1.994 | 0.003 |
| SLFN14 | 103.91 |  |  |  |
| POTEJ | 117.39 |  |  |  |
| SAA1 | 13.532 |  | 1 |  |
| SULT1A4 | 34.196 | 1.566 | 0.434 | 3.608 |
| HSPA1B | 70.051 | 1.222 | 0.778 | 1.571 |
| PPIAL4F | 18.197 | 1 |  |  |
| --- | 48.934 | 0.011 | 1.989 | 0.006 |
| --- | 56.224 | 0.008 | 1.992 | 0.004 |
| --- | 49.328 | 0.03 | 1.97 | 0.015 |
| --- | 63.485 |  | 1 |  |
| --- | 23.379 | 0.029 | 1.971 | 0.015 |
| --- | 22.83 |  |  |  |
| IGLC2 | 11.293 | 0.039 | 1.961 | 0.02 |
| IGHV1-8 | 12.992 |  | 1 |  |
| IGHV3-30-3 | 12.989 |  |  |  |
| IGHV3-30-5 | 12.947 |  |  |  |
| IGHV3-43D | 13.017 | 0.016 | 1.984 | 0.008 |
| IGKV1-13 | 12.569 |  | 1 |  |
| CALM3 | 16.837 | 1.642 | 0.358 | 4.587 |
| TUBA3C | 49.959 |  |  |  |
| GATD3A | 28.17 | 1.29 | 0.71 | 1.817 |
| RO60 | 60.67 | 1 |  |  |
| GAA | 105.32 |  |  |  |
| RRAS | 23.48 | 1.515 | 0.485 | 3.124 |
| HLA-B | 40.337 | 1.551 | 0.449 | 3.454 |
| HLA-C | 40.648 |  |  |  |
| DLAT | 68.996 | 1.525 | 0.475 | 3.211 |
| TXN | 11.737 | 1.302 | 0.698 | 1.865 |
| COX5B | 13.696 | 1.712 | 0.288 | 5.944 |
| CTSA | 54.465 | 1.316 | 0.684 | 1.924 |
| C7 | 93.517 | 0.022 | 1.978 | 0.011 |
| PRKAR1A | 42.981 | 1.185 | 0.815 | 1.454 |
| TFPI | 35.015 | 1 |  |  |
| PF4V1 | 11.553 |  |  |  |
| ESD | 31.462 | 1.541 | 0.459 | 3.357 |
| HSPD1 | 61.054 | 1.023 | 0.977 | 1.047 |
| CLU | 52.494 | 0.297 | 1.703 | 0.174 |
| HSPA5 | 72.332 | 1.374 | 0.626 | 2.195 |
| LAMC1 | 177.6 | 1 |  |  |
| HSPA8 | 70.897 | 1.334 | 0.666 | 2.003 |
| SLC2A1 | 54.083 |  |  |  |
| SLC2A3 | 53.924 | 1.361 | 0.639 | 2.13 |
| EPB41 | 97.016 |  |  |  |
| UMPS | 52.221 | 1 |  |  |
| PDHB | 39.233 |  |  |  |
| DBT | 53.486 | 1 |  |  |
| ITGAM | 127.18 | 1.105 | 0.895 | 1.235 |
| PYGB | 96.695 | 1.499 | 0.501 | 2.992 |
| RALB | 23.408 | 1.464 | 0.536 | 2.731 |
| BCR | 142.82 |  |  |  |
| SPTB | 246.47 | 1.424 | 0.576 | 2.472 |
| LAMP1 | 44.882 |  |  |  |
| ACADM | 46.588 | 1.582 | 0.418 | 3.785 |
| G6PD | 59.256 | 1.616 | 0.384 | 4.208 |
| MTHFD1 | 101.56 | 1 |  |  |
| CETP | 54.756 | 0.051 | 1.949 | 0.026 |
| EPX | 81.04 | 1.412 | 0.588 | 2.401 |
| IGF2R | 274.37 | 0.843 | 1.157 | 0.729 |
| ADH5 | 39.724 | 1.5 | 0.5 | 3 |
| PRPS2 | 34.769 | 1.604 | 0.396 | 4.051 |
| PABPC1 | 70.67 | 0.907 | 1.093 | 0.83 |
| PCNA | 28.768 | 1 |  |  |
| HARS | 57.41 | 1.33 | 0.67 | 1.985 |
| COL6A1 | 108.53 |  |  |  |
| SLC25A6 | 32.866 | 1.271 | 0.729 | 1.743 |
| F5 | 251.7 | 1.186 | 0.814 | 1.457 |
| IMPDH2 | 55.804 | 1 |  |  |
| FCGR2A | 35 |  |  |  |
| ANXA3 | 36.375 | 1.286 | 0.714 | 1.801 |
| GZMA | 28.999 | 1.223 | 0.777 | 1.574 |
| RNASE3 | 18.385 |  |  |  |
| ACTN1 | 103.06 | 1.472 | 0.528 | 2.788 |
| DEFA4 | 10.504 | 1 |  |  |
| SRC | 59.834 | 1.292 | 0.708 | 1.825 |
| PEPD | 54.548 | 1.059 | 0.941 | 1.125 |
| XRCC6 | 69.842 | 1.351 | 0.649 | 2.082 |
| XRCC5 | 82.704 | 0.901 | 1.099 | 0.82 |
| COX4I1 | 19.576 | 1.244 | 0.756 | 1.646 |
| GP1BB | 21.717 | 1.384 | 0.616 | 2.247 |
| LAMP2 | 44.96 |  |  |  |
| RNH1 | 49.973 | 1.624 | 0.376 | 4.319 |
| CYBA | 21.012 | 0.405 | 1.595 | 0.254 |
| ICAM2 | 30.654 | 1.518 | 0.482 | 3.149 |
| EEF2 | 95.337 | 1.313 | 0.687 | 1.911 |
| PDIA4 | 72.932 | 1.4 | 0.6 | 2.333 |
| C6 | 104.79 | 0.014 | 1.986 | 0.007 |
| TPT1 | 19.595 |  |  |  |
| ALAD | 36.294 | 1.352 | 0.648 | 2.086 |
| PRG2 | 25.205 | 0.088 | 1.912 | 0.046 |
| HLA-A | 40.936 | 1.052 | 0.948 | 1.11 |
| HLA-E | 40.057 | 1.506 | 0.494 | 3.049 |
| LCP1 | 70.288 | 1.133 | 0.867 | 1.307 |
| PLS3 | 70.81 | 1 |  |  |
| APEH | 81.224 | 1.609 | 0.391 | 4.115 |
| ETFA | 35.079 | 1.075 | 0.925 | 1.162 |
| GYS1 | 83.785 | 1.751 | 0.249 | 7.032 |
| PRKAR2A | 45.518 | 1 |  |  |
| CD59 | 14.177 |  |  |  |
| SELL | 42.187 |  |  |  |
| MIF | 12.476 | 0.626 | 1.374 | 0.456 |
| CD99 | 18.848 |  |  |  |
| PRF1 | 61.377 | 0.149 | 1.851 | 0.08 |
| PRKCSH | 59.425 | 1.675 | 0.325 | 5.154 |
| HCLS1 | 54.013 |  |  |  |
| FDPS | 48.275 | 1.396 | 0.604 | 2.311 |
| COX7A2 | 9.3959 |  |  |  |
| NID1 | 136.38 | 1.453 | 0.547 | 2.656 |
| AKR1A1 | 36.573 | 1.566 | 0.434 | 3.608 |
| PKM | 57.936 | 1.305 | 0.695 | 1.878 |
| ACYP2 | 11.139 | 1 |  |  |
| HSP90B1 | 92.468 | 1.371 | 0.629 | 2.18 |
| IDE | 117.97 | 1 |  |  |
| GP9 | 19.046 | 1.557 | 0.443 | 3.515 |
| MMP9 | 78.457 | 1.593 | 0.407 | 3.914 |
| COX6B1 | 10.192 | 1 |  |  |
| HNRNPL | 64.132 | 1.554 | 0.446 | 3.484 |
| DARS | 57.136 | 1.583 | 0.417 | 3.796 |
| UQCRB | 13.53 |  |  |  |
| AKR1B1 | 35.853 | 1.621 | 0.379 | 4.277 |
| ANPEP | 109.54 |  |  |  |
| RAC2 | 21.429 | 1.019 | 0.981 | 1.039 |
| GSPT1 | 55.755 | 1.065 | 0.935 | 1.139 |
| ARSA | 53.588 | 1 |  |  |
| EZR | 69.412 | 1.487 | 0.513 | 2.899 |
| UCHL3 | 26.182 |  |  |  |
| VAV1 | 98.313 | 1 |  |  |
| CD46 | 43.747 |  |  |  |
| NME1 | 17.149 | 1.571 | 0.429 | 3.662 |
| GNS | 62.081 | 1.551 | 0.449 | 3.454 |
| PHKG2 | 46.442 | 1 |  |  |
| ST6GAL1 | 46.604 | 1 |  |  |
| CD44 | 81.537 |  | 1 |  |
| NQO2 | 25.918 | 1 |  |  |
| SELP | 90.833 | 1.797 | 0.203 | 8.852 |
| CBR1 | 30.375 | 1.032 | 0.968 | 1.066 |
| ANK1 | 206.26 |  |  |  |
| HLA-A | 40.904 | 1 |  |  |
| HLA-A | 41.003 | 1 |  |  |
| ACADS | 44.297 | 1.334 | 0.666 | 2.003 |
| GLB1 | 76.074 | 1.489 | 0.511 | 2.914 |
| PECAM1 | 82.521 | 1.397 | 0.603 | 2.317 |
| PPP3CB | 59.024 |  |  |  |
| ATP2A2 | 114.76 | 1.558 | 0.442 | 3.525 |
| CD36 | 53.053 | 1.722 | 0.278 | 6.194 |
| PLCG2 | 147.87 |  |  |  |
| FAH | 46.374 | 1.528 | 0.472 | 3.237 |
| ZNF22 | 25.915 | 1 |  |  |
| HSPA6 | 71.027 |  |  |  |
| GOT1 | 46.247 | 1 |  |  |
| PRKCA | 76.749 | 1.548 | 0.452 | 3.425 |
| ITGA2 | 129.29 | 1.457 | 0.543 | 2.683 |
| PRKACA | 40.589 |  |  |  |
| CAPN2 | 79.994 | 1.807 | 0.193 | 9.363 |
| HLA-G | 38.224 |  |  |  |
| CTPS1 | 66.69 | 1.192 | 0.808 | 1.475 |
| ENG | 70.577 |  | 1 |  |
| DDX5 | 69.147 |  |  |  |
| PFKL | 85.018 | 1.417 | 0.583 | 2.431 |
| LGALS3 | 26.152 | 1.251 | 0.749 | 1.67 |
| IGFBP3 | 31.674 |  | 1 |  |
| PSMC3 | 49.203 | 1 |  |  |
| TCP1 | 60.343 | 1.379 | 0.621 | 2.221 |
| PTPN1 | 49.966 |  |  |  |
| ALOX12 | 75.693 | 1.453 | 0.547 | 2.656 |
| ITGB5 | 88.053 |  |  |  |
| ARF4 | 20.511 |  |  |  |
| VCL | 123.8 | 1.143 | 0.857 | 1.334 |
| LBP | 53.383 | 0.069 | 1.931 | 0.036 |
| PTPRA | 90.718 |  |  |  |
| NAT1 | 33.898 | 1 |  |  |
| PGAM1 | 28.804 | 1.632 | 0.368 | 4.435 |
| RCC1 | 44.969 |  |  |  |
| ATP5PF | 12.587 | 1 |  |  |
| GNAZ | 40.923 | 1.525 | 0.475 | 3.211 |
| MYL12A | 19.794 |  |  |  |
| CD58 | 28.147 | 1 |  |  |
| NCL | 76.613 | 1.708 | 0.292 | 5.849 |
| HK1 | 102.48 | 1.387 | 0.613 | 2.263 |
| TRIM21 | 54.169 | 1 |  |  |
| EIF2AK2 | 62.094 | 1 |  |  |
| SLC9A1 | 90.762 |  |  |  |
| ORM2 | 23.602 | 0.028 | 1.972 | 0.014 |
| CSNK2A2 | 41.213 | 1 |  |  |
| NCF2 | 59.761 | 1 |  |  |
| TYMP | 49.955 | 1.254 | 0.746 | 1.681 |
| ATP2B1 | 134.68 |  |  |  |
| HLA-DPA1 | 29.38 |  |  |  |
| HLA-DRB1 | 30.16 |  |  |  |
| EIF2S2 | 38.388 | 1 |  |  |
| ANXA7 | 52.739 | 1.399 | 0.601 | 2.328 |
| CD33 | 39.825 | 1 |  |  |
| AZU1 | 26.885 | 1.402 | 0.598 | 2.344 |
| TPSB2 | 30.515 | 1.537 | 0.463 | 3.32 |
| RAB3A | 24.984 | 1 |  |  |
| RAB4A | 24.389 | 1.735 | 0.265 | 6.547 |
| RAB5A | 23.658 |  |  |  |
| RAB6A | 23.593 | 1 |  |  |
| PVALB | 12.059 | 1 |  |  |
| MX1 | 75.519 |  |  |  |
| PSMB1 | 26.489 | 1.545 | 0.455 | 3.396 |
| M6PR | 30.993 | 1.547 | 0.453 | 3.415 |
| COX5A | 16.762 |  |  |  |
| LMNB1 | 66.408 | 1.004 | 0.996 | 1.008 |
| ITGAL | 128.77 | 0.767 | 1.233 | 0.622 |
| ITGAX | 127.83 |  |  |  |
| PZP | 163.86 | 0.108 | 1.892 | 0.057 |
| OGN | 33.922 | 1 |  |  |
| CAST | 76.572 |  |  |  |
| C4BPB | 28.357 |  |  |  |
| AGA | 37.208 |  |  |  |
| RASA1 | 116.4 | 1.364 | 0.636 | 2.145 |
| GSTM3 | 26.559 |  |  |  |
| ATP6V1B2 | 56.5 | 1.614 | 0.386 | 4.181 |
| CSRP1 | 20.567 | 1.727 | 0.273 | 6.326 |
| FLNA | 280.74 | 1.338 | 0.662 | 2.021 |
| ACO1 | 98.398 |  |  |  |
| VDAC1 | 30.772 | 1.283 | 0.717 | 1.789 |
| SDHB | 31.629 |  |  |  |
| CD9 | 25.416 | 1.436 | 0.564 | 2.546 |
| COMT | 30.037 | 1.531 | 0.469 | 3.264 |
| OSBP | 89.42 | 1.44 | 0.56 | 2.571 |
| PCMT1 | 24.636 | 0.863 | 1.137 | 0.759 |
| GART | 107.77 |  |  |  |
| PAICS | 47.079 | 1.562 | 0.438 | 3.566 |
| SCP2 | 58.993 |  |  |  |
| UBA1 | 117.85 | 1.495 | 0.505 | 2.96 |
| GPX3 | 25.552 | 0.162 | 1.838 | 0.088 |
| NME2 | 17.298 | 1.635 | 0.365 | 4.479 |
| FDXR | 53.836 | 1 |  |  |
| HNRNPA2B1 | 37.429 | 0.701 | 1.299 | 0.54 |
| CBL | 99.632 | 1 |  |  |
| PRKACB | 40.622 | 1.578 | 0.422 | 3.739 |
| UQCRC2 | 48.442 | 1.013 | 0.987 | 1.026 |
| CPN2 | 60.556 |  | 1 |  |
| PROZ | 44.743 |  | 1 |  |
| MMP8 | 53.411 |  |  |  |
| IGHV1-2 | 13.085 |  |  |  |
| CES1 | 62.52 | 0.691 | 1.309 | 0.528 |
| PTGS1 | 68.686 | 1.469 | 0.531 | 2.766 |
| ITGA6 | 126.6 | 1.398 | 0.602 | 2.322 |
| SFPQ | 76.149 | 1.775 | 0.225 | 7.889 |
| PPIB | 23.742 | 0.83 | 1.17 | 0.709 |
| ME2 | 65.443 | 1.08 | 0.92 | 1.174 |
| WARS | 53.165 | 1.388 | 0.612 | 2.268 |
| RPS3 | 26.688 | 1 |  |  |
| PTPRD | 214.76 | 1 |  |  |
| AHCY | 47.716 | 1.712 | 0.288 | 5.944 |
| CFL1 | 18.502 | 1.303 | 0.697 | 1.869 |
| EIF4B | 69.15 | 1.62 | 0.38 | 4.263 |
| DGKA | 82.629 | 1 |  |  |
| CPT2 | 73.776 |  |  |  |
| DTYMK | 23.819 |  |  |  |
| PRTN3 | 27.807 | 1.69 | 0.31 | 5.452 |
| KDELR1 | 24.542 | 1 |  |  |
| EEF1B2 | 24.763 | 0.891 | 1.109 | 0.803 |
| ATP5PB | 28.908 | 1.354 | 0.646 | 2.096 |
| TBXAS1 | 60.518 | 1.552 | 0.448 | 3.464 |
| ACP1 | 18.042 | 1.403 | 0.597 | 2.35 |
| ACAT1 | 45.199 | 0.795 | 1.205 | 0.66 |
| MYL9 | 19.827 | 1.793 | 0.207 | 8.662 |
| GRK2 | 79.573 | 1.495 | 0.505 | 2.96 |
| AZGP1 | 34.258 | 0.04 | 1.96 | 0.02 |
| MPST | 33.178 | 1.383 | 0.617 | 2.241 |
| RPS12 | 14.515 |  |  |  |
| DNAJB1 | 38.044 |  |  |  |
| DNAJB2 | 35.58 |  |  |  |
| ATP5F1A | 59.75 | 1.232 | 0.768 | 1.604 |
| CTSS | 37.495 |  |  |  |
| PSMA1 | 29.555 | 1.632 | 0.368 | 4.435 |
| PSMA2 | 25.898 | 0.994 | 1.006 | 0.988 |
| PSMA3 | 28.433 | 0.929 | 1.071 | 0.867 |
| PSMA4 | 29.483 | 1.516 | 0.484 | 3.132 |
| S100P | 10.4 | 1 |  |  |
| CD40 | 30.619 | 1 |  |  |
| MSN | 67.819 | 1.365 | 0.635 | 2.15 |
| DDX6 | 54.416 |  |  |  |
| U2AF2 | 53.5 | 1 |  |  |
| IVD | 46.65 |  |  |  |
| S100A4 | 11.728 | 1.305 | 0.695 | 1.878 |
| HMGB2 | 24.033 | 1.623 | 0.377 | 4.305 |
| PTBP1 | 57.221 | 1 |  |  |
| TARS | 83.434 | 1.666 | 0.334 | 4.988 |
| VARS | 140.47 | 1.597 | 0.403 | 3.963 |
| EEF1G | 50.118 | 1.433 | 0.567 | 2.527 |
| FKBP2 | 15.649 | 1 |  |  |
| MST1 | 80.319 |  | 1 |  |
| STOM | 31.73 | 1.43 | 0.57 | 2.509 |
| PON1 | 39.731 | 0.014 | 1.986 | 0.007 |
| MAOB | 58.762 | 1.736 | 0.264 | 6.576 |
| YWHAQ | 27.764 | 1.39 | 0.61 | 2.279 |
| MAPK3 | 43.135 | 1 |  |  |
| ATP6V0C | 15.736 |  |  |  |
| RPA1 | 68.137 | 1 |  |  |
| APEX1 | 35.554 | 1.546 | 0.454 | 3.405 |
| CAD | 242.98 |  |  |  |
| CALR | 48.141 | 1.695 | 0.305 | 5.557 |
| CANX | 67.567 | 1.603 | 0.397 | 4.038 |
| CFP | 51.276 | 0.099 | 1.901 | 0.052 |
| PSMB8 | 30.354 | 1.541 | 0.459 | 3.357 |
| PSMB9 | 23.264 | 0.726 | 1.274 | 0.57 |
| PSMA5 | 26.411 | 1.612 | 0.388 | 4.155 |
| PSMB4 | 29.204 |  |  |  |
| PSMB6 | 25.357 |  |  |  |
| PSMB5 | 28.48 |  |  |  |
| GSTM2 | 25.744 |  |  |  |
| NDUFS1 | 79.467 | 1.286 | 0.714 | 1.801 |
| MAPK1 | 41.389 | 1.456 | 0.544 | 2.676 |
| GRN | 63.544 | 1 |  |  |
| LAP3 | 56.166 | 1.472 | 0.528 | 2.788 |
| CD38 | 34.328 | 1.307 | 0.693 | 1.886 |
| PCSK6 | 106.42 |  |  |  |
| TPP2 | 138.35 | 1.578 | 0.422 | 3.739 |
| IMPA1 | 30.188 |  |  |  |
| PTPN6 | 67.56 | 1.604 | 0.396 | 4.051 |
| TKT | 67.877 | 1.482 | 0.518 | 2.861 |
| CASP1 | 45.158 | 1 |  |  |
| PML | 97.55 | 1 |  |  |
| SERPINA4 | 48.541 | 0.029 | 1.971 | 0.015 |
| EEF1D | 31.121 | 1 |  |  |
| OAS2 | 82.43 |  |  |  |
| MARCKS | 31.554 | 1.353 | 0.647 | 2.091 |
| ALDH4A1 | 61.719 |  |  |  |
| ERP29 | 28.993 |  |  |  |
| PRDX6 | 25.035 | 1.49 | 0.51 | 2.922 |
| BLVRB | 22.119 | 1.057 | 0.943 | 1.121 |
| PRDX5 | 22.086 | 1.53 | 0.47 | 3.255 |
| DDT | 12.712 |  |  |  |
| GCHFR | 9.6981 | 1 |  |  |
| PRDX3 | 27.692 | 1.085 | 0.915 | 1.186 |
| ATP5F1D | 17.49 | 1.264 | 0.736 | 1.717 |
| ECHS1 | 31.387 | 1.279 | 0.721 | 1.774 |
| CMPK1 | 22.222 | 1.451 | 0.549 | 2.643 |
| PEBP1 | 21.057 | 1.408 | 0.592 | 2.378 |
| PDIA3 | 56.782 | 1.438 | 0.562 | 2.559 |
| PPP2R1A | 65.308 | 1.03 | 0.97 | 1.062 |
| CD6 | 71.8 | 1 |  |  |
| PPIF | 22.04 | 1.737 | 0.263 | 6.605 |
| NMT1 | 56.806 | 1 |  |  |
| HLA-A | 41.054 | 1.729 | 0.271 | 6.38 |
| HLA-B | 40.474 | 1 |  |  |
| HLA-B | 40.539 | 1.825 | 0.175 | 10.429 |
| HLA-B | 40.38 |  |  |  |
| HLA-B | 40.478 |  |  |  |
| HLA-C | 40.964 |  |  |  |
| HLA-C | 40.885 |  |  |  |
| HLA-F | 39.061 | 1 |  |  |
| HMOX2 | 36.032 |  |  |  |
| ADSS | 50.097 |  |  |  |
| TSPO | 18.828 | 1.235 | 0.765 | 1.614 |
| ADSL | 54.889 | 1.431 | 0.569 | 2.515 |
| SRI | 21.676 | 1.382 | 0.618 | 2.236 |
| GSTT1 | 27.335 | 1 |  |  |
| SERPINB1 | 42.741 | 1.784 | 0.216 | 8.259 |
| SLC7A1 | 67.638 | 1 |  |  |
| SDHA | 72.691 | 0.963 | 1.037 | 0.929 |
| CORO1A | 51.026 | 1 | 1 | 1 |
| GDI1 | 50.582 | 1.215 | 0.785 | 1.548 |
| MAT2A | 43.66 |  |  |  |
| PRKAR2B | 46.302 |  |  |  |
| SLC6A6 | 69.829 |  |  |  |
| AKT1 | 55.686 |  |  |  |
| AKT2 | 55.768 | 1.729 | 0.271 | 6.38 |
| UQCRC1 | 52.645 | 1.384 | 0.616 | 2.247 |
| HIBADH | 35.329 |  |  |  |
| ATIC | 64.615 | 1.662 | 0.338 | 4.917 |
| APOBEC3A | 23.012 | 1 |  |  |
| HNRNPH3 | 36.926 |  |  |  |
| HNRNPH1 | 49.229 |  |  |  |
| YWHAB | 28.082 | 1.462 | 0.538 | 2.717 |
| S100A11 | 11.74 | 1.02 | 0.98 | 1.041 |
| CEACAM8 | 38.153 |  |  |  |
| INPP5B | 112.85 | 1.652 | 0.348 | 4.747 |
| PRDX2 | 21.892 | 1.203 | 0.797 | 1.509 |
| GK | 61.244 | 1.668 | 0.332 | 5.024 |
| CDA | 16.185 |  |  |  |
| DCTD | 20.016 |  |  |  |
| GBP1 | 67.93 | 1 |  |  |
| ICAM3 | 59.54 |  |  |  |
| ACSL1 | 77.942 | 1.671 | 0.329 | 5.079 |
| CDH5 | 87.527 |  | 1 |  |
| KIF5B | 109.68 | 1.686 | 0.314 | 5.369 |
| LSP1 | 37.191 |  |  |  |
| DUT | 26.563 | 1 |  |  |
| ABCC1 | 171.59 | 1 |  |  |
| MAN1A1 | 72.968 |  |  |  |
| CD68 | 37.408 |  |  |  |
| SHMT1 | 53.082 |  |  |  |
| EPHX2 | 62.615 | 1 |  |  |
| HSPA4 | 94.33 | 1.467 | 0.533 | 2.752 |
| MPI | 46.655 | 1.677 | 0.323 | 5.192 |
| PHB | 29.804 | 1.17 | 0.83 | 1.41 |
| PTPN7 | 40.529 |  |  |  |
| SERPINB6 | 42.621 | 1.695 | 0.305 | 5.557 |
| RDX | 68.563 | 1 |  |  |
| SPR | 28.048 | 1 |  |  |
| SAA4 | 14.746 |  |  |  |
| FBN1 | 312.3 |  |  |  |
| AGL | 174.76 | 1 |  |  |
| MYH9 | 226.53 | 1.273 | 0.727 | 1.751 |
| COPB2 | 102.49 | 1.118 | 0.882 | 1.268 |
| ADD1 | 80.954 | 1.355 | 0.645 | 2.101 |
| BSG | 42.2 | 1.56 | 0.44 | 3.545 |
| TIMP3 | 24.145 | 1 |  |  |
| MYH11 | 227.34 |  |  |  |
| PPM1A | 42.447 | 1.68 | 0.32 | 5.25 |
| IGFALS | 66.034 | 0.02 | 1.98 | 0.01 |
| HMGCL | 34.36 | 1.225 | 0.775 | 1.581 |
| PSMC2 | 48.633 | 1.703 | 0.297 | 5.734 |
| CHI3L1 | 42.625 | 1 |  |  |
| ATP6V1E1 | 26.145 |  |  |  |
| LONP1 | 106.49 | 1 |  |  |
| PGM1 | 61.448 | 1.602 | 0.398 | 4.025 |
| DLST | 48.755 | 1.048 | 0.952 | 1.101 |
| GMPR | 37.418 | 1.618 | 0.382 | 4.236 |
| GPX4 | 22.174 | 0.896 | 1.104 | 0.812 |
| CFHR2 | 30.65 |  | 1 |  |
| HPCAL1 | 22.313 | 1.608 | 0.392 | 4.102 |
| TAGLN2 | 22.391 | 0.937 | 1.063 | 0.881 |
| TALDO1 | 37.54 | 1.509 | 0.491 | 3.073 |
| SNCA | 14.46 |  |  |  |
| ETFB | 27.843 |  |  |  |
| ATP6V1A | 68.303 | 1.134 | 0.866 | 1.309 |
| HSPA9 | 73.68 | 1.453 | 0.547 | 2.656 |
| MPV17 | 19.733 |  |  |  |
| DDOST | 50.8 | 1.249 | 0.751 | 1.663 |
| PLA2G5 | 15.674 | 0.405 | 1.595 | 0.254 |
| IL6ST | 103.54 | 1 |  |  |
| GP5 | 60.958 | 1.253 | 0.747 | 1.677 |
| CEACAM6 | 37.194 |  |  |  |
| CCT6A | 58.024 | 1.569 | 0.431 | 3.64 |
| PSMB10 | 28.936 |  |  |  |
| ARL1 | 20.417 | 1 |  |  |
| STAT3 | 88.067 | 1.607 | 0.393 | 4.089 |
| USP8 | 127.52 | 1 |  |  |
| MDH1 | 36.426 | 1.475 | 0.525 | 2.81 |
| MDH2 | 35.503 | 1.318 | 0.682 | 1.933 |
| HADHA | 82.999 | 0.776 | 1.224 | 0.634 |
| EIF2S3 | 51.109 | 1.704 | 0.296 | 5.757 |
| MNDA | 45.836 |  |  |  |
| PTGDS | 21.029 |  | 1 |  |
| UBA7 | 111.69 | 1.508 | 0.492 | 3.065 |
| CSK | 50.704 | 1.738 | 0.262 | 6.634 |
| GARS | 83.165 | 1.576 | 0.424 | 3.717 |
| IARS | 144.5 | 1 |  |  |
| FOLR3 | 27.884 | 1 |  |  |
| ACTR1B | 42.293 | 1.899 | 0.101 | 18.802 |
| ECI1 | 32.816 |  |  |  |
| STAT1 | 87.334 |  |  |  |
| STAT6 | 94.134 | 1 |  |  |
| STAT5A | 90.646 | 1 |  |  |
| PIK3CB | 122.76 | 1 |  |  |
| MTOR | 288.89 |  |  |  |
| PI4KA | 236.83 | 1 |  |  |
| EPS15 | 98.655 |  |  |  |
| CASP3 | 31.608 | 1.172 | 0.828 | 1.415 |
| TEC | 73.58 | 1 |  |  |
| LRPPRC | 157.9 |  |  |  |
| ACAA2 | 41.924 | 0.432 | 1.568 | 0.276 |
| WAS | 52.912 |  |  |  |
| PRCP | 55.799 | 1 |  |  |
| HTT | 347.6 |  |  |  |
| ECE1 | 87.163 | 1.496 | 0.504 | 2.968 |
| PAFAH1B1 | 46.637 | 1.661 | 0.339 | 4.9 |
| CRAT | 70.857 |  |  |  |
| GRK6 | 65.99 | 1 |  |  |
| BTD | 61.132 | 0.103 | 1.897 | 0.054 |
| GPD2 | 80.852 | 0.907 | 1.093 | 0.83 |
| SYK | 72.065 | 1.028 | 0.972 | 1.058 |
| RANBP1 | 23.31 | 1 |  |  |
| NAMPT | 55.52 |  |  |  |
| AFM | 69.068 |  |  |  |
| PSMC4 | 47.366 |  |  |  |
| VDAC2 | 31.566 | 1.531 | 0.469 | 3.264 |
| ACADSB | 47.485 | 1.671 | 0.329 | 5.079 |
| USP5 | 95.785 | 1.543 | 0.457 | 3.376 |
| PHKA2 | 138.41 | 1 |  |  |
| RECQL | 73.457 | 0.482 | 1.518 | 0.318 |
| CRK | 33.83 | 1 |  |  |
| CRKL | 33.777 | 1.346 | 0.654 | 2.058 |
| BAG6 | 119.41 | 1 |  |  |
| NSF | 82.593 | 1.614 | 0.386 | 4.181 |
| MAP2K3 | 39.318 | 1 |  |  |
| BRCC3 | 36.072 | 1 |  |  |
| RPL5 | 34.362 | 0.2 | 1.8 | 0.111 |
| GNPDA1 | 32.668 | 1 |  |  |
| NEDD4 | 149.11 | 1 |  |  |
| UTRN | 394.46 |  |  |  |
| IQGAP1 | 189.25 | 1.642 | 0.358 | 4.587 |
| GYG1 | 39.383 | 1.384 | 0.616 | 2.247 |
| STT3A | 80.529 | 1.677 | 0.323 | 5.192 |
| PLA2G4A | 85.238 | 1 |  |  |
| CAPZA2 | 32.949 | 1.518 | 0.482 | 3.149 |
| CAPZB | 31.35 | 1.578 | 0.422 | 3.739 |
| QARS | 87.798 | 1.382 | 0.618 | 2.236 |
| P2RY1 | 42.071 | 1 |  |  |
| UQCRFS1 | 29.668 | 1.23 | 0.77 | 1.597 |
| ATP5PO | 23.277 | 0.776 | 1.224 | 0.634 |
| LIMS1 | 37.251 | 1.873 | 0.127 | 14.748 |
| PREP | 80.699 | 1.702 | 0.298 | 5.711 |
| PIP4K2A | 46.224 | 1.667 | 0.333 | 5.006 |
| ARCN1 | 57.21 | 1.653 | 0.347 | 4.764 |
| LSS | 83.308 | 1 |  |  |
| GCLM | 30.727 | 1.253 | 0.747 | 1.677 |
| CD151 | 28.295 | 1.517 | 0.483 | 3.141 |
| TRAPPC10 | 142.19 | 1 |  |  |
| PSMD8 | 39.611 | 1.726 | 0.274 | 6.299 |
| SERPINB10 | 45.402 | 1.557 | 0.443 | 3.515 |
| GSS | 52.384 | 1.883 | 0.117 | 16.094 |
| CCT5 | 59.67 | 1.403 | 0.597 | 2.35 |
| HSPA13 | 51.927 | 1 |  |  |
| IDH2 | 50.909 | 0.9 | 1.1 | 0.818 |
| PIK3CG | 126.45 | 1 |  |  |
| PITPNB | 31.54 | 1.614 | 0.386 | 4.181 |
| MASP1 | 79.246 |  |  |  |
| PXN | 64.505 | 1 |  |  |
| MAPKAPK2 | 45.567 | 1.388 | 0.612 | 2.268 |
| DNASE1L1 | 33.892 | 1 |  |  |
| ALDH9A1 | 53.801 | 1.726 | 0.274 | 6.299 |
| RPIA | 33.269 |  |  |  |
| LMAN1 | 57.548 | 1.188 | 0.812 | 1.463 |
| FASN | 273.42 | 1.479 | 0.521 | 2.839 |
| FNTB | 48.773 | 1 |  |  |
| CCT3 | 60.533 | 1.569 | 0.431 | 3.64 |
| ARRB1 | 47.065 | 1.776 | 0.224 | 7.929 |
| TUFM | 49.541 | 0.793 | 1.207 | 0.657 |
| INPP1 | 43.998 | 1 |  |  |
| SRP9 | 10.112 |  |  |  |
| AARS | 106.81 | 1.589 | 0.411 | 3.866 |
| CARS | 85.472 |  |  |  |
| SARS | 58.777 | 1.617 | 0.383 | 4.222 |
| PPM1F | 49.83 | 1.463 | 0.537 | 2.724 |
| PSMB3 | 22.949 |  |  |  |
| PSMB2 | 22.836 | 0.615 | 1.385 | 0.444 |
| ACADVL | 70.389 | 1.307 | 0.693 | 1.886 |
| TMED10 | 24.976 | 1.43 | 0.57 | 2.509 |
| RGS6 | 54.422 | 1 |  |  |
| VEGFC | 46.883 | 1 |  |  |
| EIF2B2 | 38.989 | 1 |  |  |
| HINT1 | 13.802 |  |  |  |
| FHIT | 16.858 | 1 |  |  |
| RGS19 | 24.635 | 1 |  |  |
| NDUFV1 | 50.817 |  |  |  |
| GZMK | 28.882 | 1.707 | 0.293 | 5.826 |
| NT5C2 | 64.969 |  |  |  |
| SELENOP | 43.173 |  |  |  |
| CAMP | 19.301 | 1.536 | 0.464 | 3.31 |
| GMPS | 76.715 | 1 |  |  |
| GNAQ | 42.142 | 1.244 | 0.756 | 1.646 |
| IDH3A | 39.591 |  |  |  |
| SULT1A1 | 34.165 | 1.469 | 0.531 | 2.766 |
| CRIP1 | 8.5328 |  |  |  |
| PPOX | 50.765 | 1 |  |  |
| GDI2 | 50.663 | 1.173 | 0.827 | 1.418 |
| CPT1A | 88.367 | 1.253 | 0.747 | 1.677 |
| GATM | 48.455 | 1 |  |  |
| SERPINB8 | 42.766 |  |  |  |
| SERPINB9 | 42.403 | 1.641 | 0.359 | 4.571 |
| SERPINH1 | 46.44 | 1 |  |  |
| VASP | 39.829 | 0.67 | 1.33 | 0.504 |
| DNM2 | 98.063 | 1.524 | 0.476 | 3.202 |
| METAP2 | 52.891 | 1 |  |  |
| NUDT2 | 16.829 | 1 |  |  |
| LRBA | 319.1 | 1.599 | 0.401 | 3.988 |
| PPT1 | 34.193 | 1 |  |  |
| CCT8 | 59.62 | 1.529 | 0.471 | 3.246 |
| CCT4 | 57.924 | 1.307 | 0.693 | 1.886 |
| ANXA11 | 54.389 | 0.992 | 1.008 | 0.984 |
| FXR1 | 69.72 | 1 |  |  |
| GZMM | 27.545 |  |  |  |
| RAB5C | 23.482 | 1.084 | 0.916 | 1.183 |
| RAB7A | 23.489 | 1.292 | 0.708 | 1.825 |
| RAB9A | 22.837 | 1 |  |  |
| RAB13 | 22.774 |  |  |  |
| RAB27A | 24.868 |  |  |  |
| DUSP3 | 20.478 | 1.608 | 0.392 | 4.102 |
| GALK1 | 42.272 |  |  |  |
| SSR4 | 18.998 | 1.718 | 0.282 | 6.092 |
| BCAP31 | 27.991 | 1.238 | 0.762 | 1.625 |
| P2RX1 | 44.98 | 1.452 | 0.548 | 2.65 |
| TPMT | 28.18 | 1.09 | 0.91 | 1.198 |
| RENBP | 48.83 | 1.491 | 0.509 | 2.929 |
| HCFC1 | 208.73 | 1 |  |  |
| HSD17B4 | 79.685 | 1.66 | 0.34 | 4.882 |
| PSMD7 | 37.025 |  |  |  |
| SGSH | 56.695 | 1 |  |  |
| ARSD | 64.859 | 1 |  |  |
| STAT5B | 89.865 | 1.524 | 0.476 | 3.202 |
| DYNLT3 | 13.062 | 1 |  |  |
| VAMP7 | 24.935 |  |  |  |
| RPS6KA3 | 83.735 | 1.543 | 0.457 | 3.376 |
| HDGF | 26.788 | 1 |  |  |
| PRELP | 43.809 |  |  |  |
| HNRNPA3 | 39.594 | 1.233 | 0.767 | 1.608 |
| PGD | 53.139 | 1.701 | 0.299 | 5.689 |
| RAP1GDS1 | 66.316 | 1.425 | 0.575 | 2.478 |
| JAK3 | 125.1 |  |  |  |
| POLR2H | 17.143 | 1 |  |  |
| ARHGDIA | 23.207 | 1.411 | 0.589 | 2.396 |
| ARHGDIB | 22.988 | 1.359 | 0.641 | 2.12 |
| HNRNPF | 45.671 | 1.767 | 0.233 | 7.584 |
| STAT2 | 97.915 | 1 |  |  |
| SMS | 41.268 | 1.839 | 0.161 | 11.422 |
| HK3 | 99.024 | 1.552 | 0.448 | 3.464 |
| THOP1 | 78.839 | 1 |  |  |
| CAPZA1 | 32.922 | 1.43 | 0.57 | 2.509 |
| BLVRA | 33.428 | 1.273 | 0.727 | 1.751 |
| SLC25A1 | 34.012 | 1.378 | 0.622 | 2.215 |
| PPP5C | 56.878 | 1 |  |  |
| ACLY | 120.84 | 1.522 | 0.478 | 3.184 |
| METAP1 | 43.215 | 1.555 | 0.445 | 3.494 |
| SUCLG1 | 36.249 |  |  |  |
| COPB1 | 107.14 | 1.512 | 0.488 | 3.098 |
| COPA | 138.34 | 1.03 | 0.97 | 1.062 |
| CTSC | 51.853 | 1.067 | 0.933 | 1.144 |
| CLTCL1 | 187.03 | 1 |  |  |
| AP2S1 | 17.018 | 1.449 | 0.551 | 2.63 |
| SEC24C | 118.32 | 1 |  |  |
| SUB1 | 14.395 | 1 |  |  |
| CRISP3 | 27.63 |  |  |  |
| RARS | 75.378 | 1.418 | 0.582 | 2.436 |
| YARS | 59.143 | 1.485 | 0.515 | 2.883 |
| USP14 | 56.068 | 1.541 | 0.459 | 3.357 |
| PRKAG1 | 37.579 | 1.405 | 0.595 | 2.361 |
| ATP1B3 | 31.512 |  |  |  |
| NAGLU | 82.265 | 1 |  |  |
| AK2 | 26.477 | 1.384 | 0.616 | 2.247 |
| ALDH18A1 | 87.301 |  |  |  |
| NAPA | 33.232 | 1.486 | 0.514 | 2.891 |
| DRG2 | 40.746 | 1 |  |  |
| PLTP | 54.739 | 0.105 | 1.895 | 0.055 |
| CSE1L | 110.42 | 1.637 | 0.363 | 4.51 |
| VCP | 89.321 | 1.522 | 0.478 | 3.184 |
| HADHB | 51.294 |  |  |  |
| NCKAP1L | 128.15 | 1.587 | 0.413 | 3.843 |
| NAP1L1 | 45.374 | 1.291 | 0.709 | 1.821 |
| CASP6 | 33.31 |  |  |  |
| ADK | 40.545 | 1 |  |  |
| LAMB2 | 195.98 |  |  |  |
| SEC13 | 35.54 | 1.772 | 0.228 | 7.772 |
| NPEPPS | 103.28 | 1.426 | 0.574 | 2.484 |
| HNRNPH2 | 49.263 | 1.447 | 0.553 | 2.617 |
| OXCT1 | 56.157 | 1.499 | 0.501 | 2.992 |
| EIF3B | 92.48 | 0.581 | 1.419 | 0.409 |
| BID | 21.994 |  |  |  |
| ATP5MF | 10.918 | 1.613 | 0.387 | 4.168 |
| MARS | 101.11 |  |  |  |
| CTSW | 42.12 |  |  |  |
| TCL1A | 13.459 | 1 |  |  |
| ATP5MPL | 6.662 |  |  |  |
| LGALS4 | 35.941 | 1 |  |  |
| EIF6 | 26.599 |  |  |  |
| SLC37A1 | 57.647 | 1 |  |  |
| TMEM33 | 27.978 |  |  |  |
| RAB38 | 23.712 |  |  |  |
| CORO7 | 100.6 |  |  |  |
| GSDMD | 52.8 | 1 |  |  |
| EPPK1 | 555.65 |  |  |  |
| LOXL3 | 83.166 | 1 |  |  |
| MTPN | 12.895 | 1.48 | 0.52 | 2.846 |
| DEFA3 | 10.245 | 1.792 | 0.208 | 8.615 |
| GNG2 | 7.8501 | 0.655 | 1.345 | 0.487 |
| ARPC4 | 19.667 | 1.549 | 0.451 | 3.435 |
| TPI1 | 30.791 | 1.376 | 0.624 | 2.205 |
| EIF3E | 52.22 |  |  |  |
| SEC61B | 9.9743 |  |  |  |
| MYL6 | 16.93 | 1.353 | 0.647 | 2.091 |
| ACTB | 41.736 | 1.369 | 0.631 | 2.17 |
| EIF4A1 | 46.153 | 1.359 | 0.641 | 2.12 |
| PRPS1 | 34.834 | 1.545 | 0.455 | 3.396 |
| PSMA6 | 27.399 | 1.43 | 0.57 | 2.509 |
| S100A10 | 11.203 | 0.781 | 1.219 | 0.641 |
| CDC42 | 21.258 | 1.179 | 0.821 | 1.436 |
| DSTN | 18.506 | 1.541 | 0.459 | 3.357 |
| RAB8A | 23.668 | 1.532 | 0.468 | 3.274 |
| SPCS3 | 20.313 | 1 |  |  |
| RAB4B | 23.586 | 1.347 | 0.653 | 2.063 |
| RAB2A | 23.545 | 1.528 | 0.472 | 3.237 |
| RAB5B | 23.707 |  |  |  |
| RAB10 | 22.541 | 1.384 | 0.616 | 2.247 |
| UBE2M | 20.9 |  |  |  |
| UBE2K | 22.406 |  |  |  |
| UBE2N | 17.138 |  |  |  |
| RAB14 | 23.897 | 1.515 | 0.485 | 3.124 |
| ACTR3 | 47.371 | 1.519 | 0.481 | 3.158 |
| ACTR2 | 44.76 | 1.655 | 0.345 | 4.797 |
| ACTR1A | 42.613 | 1.265 | 0.735 | 1.721 |
| COPS2 | 51.596 |  |  |  |
| ABCE1 | 67.314 | 1.401 | 0.599 | 2.339 |
| RAP1B | 20.825 | 1.256 | 0.744 | 1.688 |
| RAP2B | 20.504 | 1.458 | 0.542 | 2.69 |
| RPL15 | 24.146 | 0.651 | 1.349 | 0.483 |
| MAGOH | 17.163 | 1.523 | 0.477 | 3.193 |
| ATP6V0D1 | 40.329 |  |  |  |
| RHOA | 21.768 | 1.289 | 0.711 | 1.813 |
| HSPE1 | 10.932 | 1.137 | 0.863 | 1.317 |
| SEC61A1 | 52.264 | 1.175 | 0.825 | 1.424 |
| LYZ | 16.537 | 0.841 | 1.159 | 0.726 |
| STXBP1 | 67.568 |  |  |  |
| B2M | 13.714 | 1.263 | 0.737 | 1.714 |
| DAD1 | 12.497 |  |  |  |
| NPC2 | 16.57 | 1.319 | 0.681 | 1.937 |
| COPZ1 | 20.198 | 1.604 | 0.396 | 4.051 |
| UFM1 | 9.1175 | 1 |  |  |
| AP1S1 | 18.733 |  |  |  |
| NUTF2 | 14.478 |  |  |  |
| HNRNPK | 50.976 | 1.37 | 0.63 | 2.175 |
| YWHAG | 28.302 | 1.38 | 0.62 | 2.226 |
| PPP1CA | 37.512 | 1.744 | 0.256 | 6.812 |
| PPP1CB | 37.186 | 1.471 | 0.529 | 2.781 |
| PSMC1 | 49.184 | 1.316 | 0.684 | 1.924 |
| PSMC5 | 45.626 |  |  |  |
| RPS15A | 14.839 | 1.413 | 0.587 | 2.407 |
| UBE2H | 20.655 | 1.222 | 0.778 | 1.571 |
| YWHAE | 29.174 | 1.579 | 0.421 | 3.751 |
| SNRPD3 | 13.916 | 1.662 | 0.338 | 4.917 |
| TMSB4X | 5.0526 | 1.616 | 0.384 | 4.208 |
| ARF6 | 20.082 |  |  |  |
| SELENOT | 22.324 | 1 |  |  |
| ETF1 | 49.03 | 1 |  |  |
| CNBP | 19.463 | 1 |  |  |
| PPP2CB | 35.575 | 1.724 | 0.276 | 6.246 |
| HIST1H4A | 11.367 |  |  |  |
| RAB1A | 22.677 | 1.262 | 0.738 | 1.71 |
| RAN | 24.423 | 1.37 | 0.63 | 2.175 |
| RAP1A | 20.987 |  |  |  |
| UBE2D2 | 16.735 | 1.55 | 0.45 | 3.444 |
| RPS28 | 7.8409 |  |  |  |
| GNB1 | 37.377 | 1.481 | 0.519 | 2.854 |
| GNB2 | 37.331 | 1.728 | 0.272 | 6.353 |
| PPIA | 18.012 | 1.36 | 0.64 | 2.125 |
| FKBP1A | 11.951 | 0.653 | 1.347 | 0.485 |
| UBA52 | 14.728 | 1.62 | 0.38 | 4.263 |
| GRB2 | 25.206 | 1.721 | 0.279 | 6.168 |
| RAC1 | 21.45 | 1.231 | 0.769 | 1.601 |
| AP2B1 | 104.55 | 1.598 | 0.402 | 3.975 |
| GNAI1 | 40.361 |  |  |  |
| YWHAZ | 27.745 | 1.333 | 0.667 | 1.999 |
| DYNLL1 | 10.366 | 1.526 | 0.474 | 3.219 |
| SKP1 | 18.658 | 1 |  |  |
| RPS21 | 9.1113 |  |  |  |
| EIF5A | 16.832 | 1.339 | 0.661 | 2.026 |
| RACK1 | 35.076 | 0.889 | 1.111 | 0.8 |
| ACTG1 | 41.792 |  |  |  |
| PPP2CA | 35.594 | 1 |  |  |
| YBX1 | 35.924 | 1 |  |  |
| SEC11A | 20.625 | 1 |  |  |
| CSNK2B | 24.942 |  |  |  |
| TPM4 | 28.521 | 1.308 | 0.692 | 1.89 |
| ACTC1 | 42.019 | 1.333 | 0.667 | 1.999 |
| UBE2L3 | 17.861 | 1 |  |  |
| EEF1A1 | 50.14 | 1.489 | 0.511 | 2.914 |
| TUBA4A | 49.924 | 0.828 | 1.172 | 0.706 |
| TUBB4B | 49.83 | 0.822 | 1.178 | 0.698 |
| CSNK2A1 | 45.143 | 1.546 | 0.454 | 3.405 |
| PAFAH1B2 | 25.569 |  |  |  |
| HBB | 15.998 | 0.079 | 1.921 | 0.041 |
| NOMO3 | 134.13 |  |  |  |
| SIRPA | 54.966 | 1.111 | 0.889 | 1.25 |
| EIF4G2 | 102.36 |  |  |  |
| CSNK1G2 | 47.457 |  |  |  |
| CCT2 | 57.488 | 1.258 | 0.742 | 1.695 |
| GSTO1 | 27.566 | 1.289 | 0.711 | 1.813 |
| RELN | 388.38 | 1 |  |  |
| PRKDC | 469.08 | 1.162 | 0.838 | 1.387 |
| GPLD1 | 92.335 |  |  |  |
| LCN2 | 22.588 | 0.76 | 1.24 | 0.613 |
| IFI35 | 31.546 | 1 |  |  |
| NUCB2 | 50.222 |  |  |  |
| S100A12 | 10.575 | 1.843 | 0.157 | 11.739 |
| BASP1 | 22.693 | 1.325 | 0.675 | 1.963 |
| IGLV3-21 | 12.446 |  | 1 |  |
| ARF1 | 20.697 | 1.139 | 0.861 | 1.323 |
| ARF5 | 20.529 | 1.28 | 0.72 | 1.778 |
| ERH | 12.259 | 1.448 | 0.552 | 2.623 |
| RHOG | 21.308 | 1.754 | 0.246 | 7.13 |
| H3F3A | 15.328 |  | 1 |  |
| CCZ1 | 55.866 | 1 |  |  |
| DAB2 | 82.447 | 1 |  |  |
| HSPG2 | 468.83 |  |  |  |
| ARHGAP4 | 105.02 | 1 |  |  |
| EFNB1 | 38.006 | 1 |  |  |
| ATP2C1 | 100.58 | 1.708 | 0.292 | 5.849 |
| MPP1 | 52.296 | 1.332 | 0.668 | 1.994 |
| PITPNA | 31.806 |  |  |  |
| SLC25A3 | 40.094 | 1.193 | 0.807 | 1.478 |
| CLTC | 191.61 | 1.424 | 0.576 | 2.472 |
| REEP5 | 21.493 |  |  |  |
| SORD | 38.324 | 1 |  |  |
| HNRNPU | 90.583 | 1.727 | 0.273 | 6.326 |
| SPTBN1 | 274.61 | 1.626 | 0.374 | 4.348 |
| SET | 33.488 | 1.597 | 0.403 | 3.963 |
| SRSF2 | 25.476 | 1 |  |  |
| AMPD2 | 100.69 | 1.182 | 0.818 | 1.445 |
| CTBS | 43.759 | 0.719 | 1.281 | 0.561 |
| FABP5 | 15.164 | 1.509 | 0.491 | 3.073 |
| CAP1 | 51.901 | 1.065 | 0.935 | 1.139 |
| PFKP | 85.595 | 1.587 | 0.413 | 3.843 |
| EWSR1 | 68.477 | 1 |  |  |
| GUCY1A1 | 77.452 | 1.296 | 0.704 | 1.841 |
| GUCY1B1 | 70.514 | 1.593 | 0.407 | 3.914 |
| OGDH | 115.93 | 1.027 | 0.973 | 1.055 |
| MAP2K1 | 43.439 |  |  |  |
| FKBP4 | 51.804 |  |  |  |
| SLC25A11 | 34.061 | 1.296 | 0.704 | 1.841 |
| DST | 860.65 |  | 1 |  |
| GSTM4 | 25.561 | 1 |  |  |
| LMNB2 | 69.948 |  |  |  |
| PLAUR | 36.978 | 1 |  |  |
| TAP1 | 87.217 | 1.48 | 0.52 | 2.846 |
| TAP2 | 75.663 | 1.549 | 0.451 | 3.435 |
| CFHR1 | 37.65 | 0.068 | 1.932 | 0.035 |
| CEBPZ | 120.97 | 1 |  |  |
| RELA | 60.218 |  |  |  |
| GBE1 | 80.473 | 1.671 | 0.329 | 5.079 |
| EIF4G1 | 175.49 |  |  |  |
| HGFAC | 70.681 |  |  |  |
| PRKCQ | 81.864 | 1 |  |  |
| GLO1 | 20.777 | 1.5 | 0.5 | 3 |
| HLA-B | 40.505 |  |  |  |
| SSBP1 | 17.259 | 1.57 | 0.43 | 3.651 |
| YWHAH | 28.218 | 1.51 | 0.49 | 3.082 |
| DNM1 | 97.407 | 1.854 | 0.146 | 12.699 |
| PTPN12 | 88.105 | 1.55 | 0.45 | 3.444 |
| CLC | 16.453 |  |  |  |
| PTK2 | 119.23 | 1.444 | 0.556 | 2.597 |
| PRKCD | 77.504 | 1.618 | 0.382 | 4.236 |
| CALD1 | 93.23 | 0.304 | 1.696 | 0.179 |
| SLC18A2 | 55.712 |  |  |  |
| PTPN11 | 68.436 | 1.494 | 0.506 | 2.953 |
| BTK | 76.28 | 1.366 | 0.634 | 2.155 |
| PPAT | 57.398 | 1 |  |  |
| GFPT1 | 78.806 |  |  |  |
| PSME1 | 28.723 | 1.513 | 0.487 | 3.107 |
| PRDX1 | 22.11 | 1.459 | 0.541 | 2.697 |
| EGR3 | 42.613 |  |  |  |
| C1QBP | 31.362 | 1.148 | 0.852 | 1.347 |
| CKAP4 | 66.022 | 1 |  |  |
| CD69 | 22.559 | 1 |  |  |
| BAX | 21.184 | 1.589 | 0.411 | 3.866 |
| BCL2L1 | 26.049 |  |  |  |
| SOS1 | 152.46 | 1 |  |  |
| LRP1 | 504.6 |  |  |  |
| SRSF1 | 27.744 |  |  |  |
| ARHGAP1 | 50.435 | 1.086 | 0.914 | 1.188 |
| PPP3CA | 58.687 |  |  |  |
| DHX9 | 140.96 | 1.192 | 0.808 | 1.475 |
| CRYZ | 35.206 | 1 |  |  |
| LGALS3BP | 65.33 | 0.691 | 1.309 | 0.528 |
| RBBP4 | 47.655 |  |  |  |
| AHNAK | 629.09 | 0.523 | 1.477 | 0.354 |
| APOBR | 114.87 |  |  |  |
| NEXN | 80.657 | 1.485 | 0.515 | 2.883 |
| MGAT2 | 51.55 | 1 |  |  |
| GALNT2 | 64.732 |  |  |  |
| GALNT1 | 64.218 | 1 |  |  |
| AP1B1 | 104.64 | 1.479 | 0.521 | 2.839 |
| BST1 | 35.724 | 1.15 | 0.85 | 1.353 |
| PMPCA | 58.252 | 1 |  |  |
| WASHC5 | 134.28 |  |  |  |
| ASPH | 85.862 | 1 |  |  |
| EFEMP1 | 54.64 | 0.413 | 1.587 | 0.26 |
| STX4 | 34.18 |  |  |  |
| MAP4K2 | 91.555 |  |  |  |
| DPYD | 111.4 |  |  |  |
| AIMP1 | 34.352 | 1 |  |  |
| ILF2 | 43.062 | 1.194 | 0.806 | 1.481 |
| LMAN2 | 40.228 | 1.467 | 0.533 | 2.752 |
| PTPRJ | 145.94 | 1.543 | 0.457 | 3.376 |
| TRAP1 | 80.109 |  |  |  |
| TRAF2 | 55.859 |  | 1 |  |
| DLG1 | 100.45 |  |  |  |
| BNIP2 | 36.017 | 1.372 | 0.628 | 2.185 |
| TIAM1 | 177.51 | 1.822 | 0.178 | 10.236 |
| ECH1 | 35.816 | 1.113 | 0.887 | 1.255 |
| STK4 | 55.63 |  |  |  |
| FLII | 144.75 | 1.81 | 0.19 | 9.526 |
| COASY | 62.328 | 1 |  |  |
| ACACA | 265.55 | 1 |  |  |
| LCP2 | 60.187 | 1.638 | 0.362 | 4.525 |
| GPS1 | 55.536 |  |  |  |
| SPP2 | 24.337 |  |  |  |
| USP4 | 108.56 | 1 |  |  |
| MTAP | 31.236 | 1.362 | 0.638 | 2.135 |
| PRKAA1 | 64.009 |  |  |  |
| HNRNPA0 | 30.84 | 1 |  |  |
| AIMP2 | 35.348 | 1.784 | 0.216 | 8.259 |
| PRDX4 | 30.54 | 1.573 | 0.427 | 3.684 |
| PAK2 | 58.042 | 1 |  |  |
| PSMD2 | 100.2 | 1.44 | 0.56 | 2.571 |
| MMRN1 | 138.11 | 1.293 | 0.707 | 1.829 |
| DNAJC3 | 57.579 |  |  |  |
| CHIT1 | 51.681 | 1 |  |  |
| NME3 | 19.015 | 1 |  |  |
| TRIM28 | 88.549 | 1 |  |  |
| G3BP1 | 52.164 | 1.452 | 0.548 | 2.65 |
| KCNAB2 | 41 |  |  |  |
| PTK7 | 118.39 | 1 |  |  |
| IFIT5 | 55.846 | 1 |  |  |
| EIF3I | 36.501 | 1.812 | 0.188 | 9.638 |
| UBE2V1 | 16.495 | 1.537 | 0.463 | 3.32 |
| ILK | 51.419 | 1.554 | 0.446 | 3.484 |
| NNT | 113.89 | 1.506 | 0.494 | 3.049 |
| ADAM9 | 90.555 | 1 |  |  |
| FKBP5 | 51.212 |  |  |  |
| MYO9B | 243.4 | 1 |  |  |
| ROCK1 | 158.17 | 1.378 | 0.622 | 2.215 |
| PICALM | 70.754 | 1.289 | 0.711 | 1.813 |
| MTM1 | 69.931 | 1 |  |  |
| MTX1 | 51.462 |  |  |  |
| ASAH1 | 44.659 | 1.352 | 0.648 | 2.086 |
| PIN1 | 18.243 |  |  |  |
| RIPK1 | 75.93 |  |  |  |
| CAMK2D | 56.369 | 1 |  |  |
| DCTN2 | 44.23 | 0.433 | 1.567 | 0.276 |
| NAE1 | 60.246 | 1.568 | 0.432 | 3.63 |
| IQGAP2 | 180.58 | 1.397 | 0.603 | 2.317 |
| STIM1 | 77.422 | 1.282 | 0.718 | 1.786 |
| CUL1 | 89.677 | 1 |  |  |
| CUL2 | 86.982 |  |  |  |
| CUL3 | 88.929 | 1 |  |  |
| CUL4A | 87.679 | 0.803 | 1.197 | 0.671 |
| TSTA3 | 35.892 | 1 |  |  |
| RAB32 | 24.997 | 1.593 | 0.407 | 3.914 |
| FHL1 | 36.263 | 1.753 | 0.247 | 7.097 |
| MOGS | 91.916 | 1.393 | 0.607 | 2.295 |
| APOF | 35.399 |  | 1 |  |
| SPTAN1 | 284.54 | 1.968 | 0.032 | 61.5 |
| DDX39B | 48.991 | 1.124 | 0.876 | 1.283 |
| BLMH | 52.562 | 1.576 | 0.424 | 3.717 |
| SNTB1 | 58.06 | 1.261 | 0.739 | 1.706 |
| CBFB | 21.508 | 1 |  |  |
| PRKG1 | 76.364 | 1.833 | 0.167 | 10.976 |
| CKAP5 | 225.49 | 1.325 | 0.675 | 1.963 |
| CIRBP | 18.648 |  |  |  |
| CAMK1 | 41.337 | 1.41 | 0.59 | 2.39 |
| COTL1 | 15.945 | 1.417 | 0.583 | 2.431 |
| HNRNPD | 38.434 |  |  |  |
| SCARB2 | 54.29 | 1 |  |  |
| NID2 | 151.25 | 1.526 | 0.474 | 3.219 |
| IL18 | 22.326 | 1 |  |  |
| DPYS | 56.629 |  |  |  |
| DAG1 | 97.44 |  |  |  |
| SEPT6 | 49.716 | 1.573 | 0.427 | 3.684 |
| EIF3A | 166.57 |  |  |  |
| ARHGEF7 | 90.011 | 1 |  |  |
| EFR3A | 92.923 | 1 |  |  |
| SCARF1 | 87.386 |  |  |  |
| MLEC | 32.233 | 1.566 | 0.434 | 3.608 |
| TTLL12 | 74.403 | 1 |  |  |
| FHL2 | 32.193 | 1 |  |  |
| DCTN1 | 141.69 | 1.693 | 0.307 | 5.515 |
| DYNC1H1 | 532.4 | 1.816 | 0.184 | 9.87 |
| EIF2B1 | 33.712 |  |  |  |
| CTTN | 61.585 |  |  |  |
| FLOT2 | 47.064 | 1.411 | 0.589 | 2.396 |
| TRIM25 | 70.973 |  |  |  |
| PTK2B | 115.87 | 1 |  |  |
| GNA13 | 44.049 | 1.325 | 0.675 | 1.963 |
| GAMT | 26.318 |  |  |  |
| LRRC32 | 71.978 | 1 |  |  |
| PDE3A | 124.98 |  |  |  |
| BECN1 | 51.896 |  |  |  |
| RBM39 | 59.379 | 1 |  |  |
| HABP2 | 62.671 | 0.017 | 1.983 | 0.009 |
| PDIA5 | 59.594 | 1.512 | 0.488 | 3.098 |
| PRPSAP1 | 39.393 |  |  |  |
| ITPR2 | 308.06 | 1.69 | 0.31 | 5.452 |
| INPP5A | 47.819 | 1.34 | 0.66 | 2.03 |
| ITPR1 | 313.93 | 1.572 | 0.428 | 3.673 |
| RASA3 | 95.698 | 1.492 | 0.508 | 2.937 |
| IRF3 | 47.219 | 1 |  |  |
| CLINT1 | 68.259 | 0.656 | 1.344 | 0.488 |
| NCOA6 | 219.14 | 1 |  |  |
| MESD | 26.076 | 1 |  |  |
| GANAB | 106.87 | 1.054 | 0.946 | 1.114 |
| MFSD10 | 48.339 |  |  |  |
| LBR | 70.702 |  |  |  |
| MVP | 99.326 | 1.448 | 0.552 | 2.623 |
| LTBP1 | 186.79 | 1.491 | 0.509 | 2.929 |
| GOLGB1 | 376.01 |  |  |  |
| LASP1 | 29.717 | 1.079 | 0.921 | 1.172 |
| KPNB1 | 97.169 | 1.31 | 0.69 | 1.899 |
| NUMA1 | 238.26 |  |  |  |
| SLMAP | 95.197 | 1 |  |  |
| GAPVD1 | 164.98 |  |  |  |
| SPCS2 | 25.003 | 1.294 | 0.706 | 1.833 |
| EMC2 | 34.833 | 1 |  |  |
| PSMD6 | 45.531 | 1.609 | 0.391 | 4.115 |
| SEPT2 | 41.487 | 1.326 | 0.674 | 1.967 |
| EFTUD2 | 109.43 |  |  |  |
| SNX17 | 52.901 |  |  |  |
| ARL6IP1 | 23.362 |  |  |  |
| RAB3GAP1 | 110.52 | 1 |  |  |
| KARS | 68.047 |  |  |  |
| ARHGEF6 | 87.498 | 1 |  |  |
| EIF4H | 27.385 | 1 |  |  |
| ACOX1 | 74.423 | 1.365 | 0.635 | 2.15 |
| PDIA6 | 48.121 | 1.266 | 0.734 | 1.725 |
| PCOLCE | 47.972 |  |  |  |
| PDK1 | 49.244 | 1 |  |  |
| PEA15 | 15.04 | 1 |  |  |
| EBP | 26.352 |  |  |  |
| PMVK | 21.995 |  |  |  |
| PLEC | 531.78 | 1.315 | 0.685 | 1.92 |
| PON2 | 39.38 |  |  |  |
| PON3 | 39.607 |  |  |  |
| PPP2R5A | 56.193 | 1 |  |  |
| PPA1 | 32.66 | 1 |  |  |
| PTGES3 | 18.697 | 1.547 | 0.453 | 3.415 |
| STK38 | 54.19 | 1 |  |  |
| PTPA | 40.667 | 1 |  |  |
| RAB35 | 23.025 | 1.592 | 0.408 | 3.902 |
| RCN1 | 38.89 | 1 |  |  |
| TMED2 | 22.761 | 1.281 | 0.719 | 1.782 |
| PCBP1 | 37.497 | 1.304 | 0.696 | 1.874 |
| PCBP2 | 38.58 | 1.504 | 0.496 | 3.032 |
| ELOC | 12.473 | 1.683 | 0.317 | 5.309 |
| ELOB | 13.133 | 1.557 | 0.443 | 3.515 |
| RHEB | 20.497 |  |  |  |
| UBE3C | 123.92 |  |  |  |
| ANGPT1 | 57.512 |  |  |  |
| SF3B3 | 135.58 |  |  |  |
| RSU1 | 31.54 | 1.537 | 0.463 | 3.32 |
| RPS6KA1 | 82.722 |  |  |  |
| SF3B4 | 44.385 | 1 |  |  |
| SEC23A | 86.16 | 1.665 | 0.335 | 4.97 |
| SEC23B | 86.478 | 1.124 | 0.876 | 1.283 |
| CYTH1 | 46.412 | 1 |  |  |
| SURF1 | 33.331 |  |  |  |
| MAPRE2 | 37.031 | 0.717 | 1.283 | 0.559 |
| TGFBI | 74.68 |  | 1 |  |
| TSN | 26.183 | 1.358 | 0.642 | 2.115 |
| TRIP10 | 68.351 | 1 |  |  |
| MAPRE1 | 29.999 |  |  |  |
| NSDHL | 41.9 | 1 |  |  |
| MYLK | 210.71 | 1.745 | 0.255 | 6.843 |
| CD226 | 38.614 | 1.304 | 0.696 | 1.874 |
| RAB30 | 23.058 | 1 |  |  |
| SMAD2 | 52.306 | 1 |  |  |
| TBCE | 59.345 | 1 |  |  |
| UBE2V2 | 16.363 |  |  |  |
| STXBP2 | 66.452 | 1.213 | 0.787 | 1.541 |
| NEDD8 | 9.0714 | 1.782 | 0.218 | 8.174 |
| ADIPOQ | 26.413 |  | 1 |  |
| ATP6AP1 | 52.025 |  |  |  |
| RAB11B | 24.488 | 0.944 | 1.056 | 0.894 |
| ZYX | 61.277 | 1.125 | 0.875 | 1.286 |
| ETFDH | 68.495 | 1 |  |  |
| SEPT7 | 50.679 | 1.411 | 0.589 | 2.396 |
| ADRM1 | 42.153 |  |  |  |
| PSMD5 | 56.195 | 1 |  |  |
| CSRP2 | 20.954 | 1 |  |  |
| DDB1 | 126.97 | 1.511 | 0.489 | 3.09 |
| MAPK14 | 41.293 | 1.641 | 0.359 | 4.571 |
| CDC37 | 44.468 |  |  |  |
| DPYSL2 | 62.293 | 1.465 | 0.535 | 2.738 |
| RBBP7 | 47.82 | 1 |  |  |
| FXN | 23.135 |  |  |  |
| ECM1 | 60.673 | 0.086 | 1.914 | 0.045 |
| SRSF7 | 27.366 | 1 |  |  |
| CPSF6 | 59.209 |  |  |  |
| DBN1 | 71.428 | 1.481 | 0.519 | 2.854 |
| MAPKAPK3 | 42.987 |  |  |  |
| DECR1 | 36.067 | 1.023 | 0.977 | 1.047 |
| MAN2A1 | 131.14 | 1 |  |  |
| TST | 33.429 | 0.835 | 1.165 | 0.717 |
| HAGH | 33.805 |  |  |  |
| NDUFA9 | 42.509 | 1.066 | 0.934 | 1.141 |
| RTN1 | 83.617 |  |  |  |
| PCK2 | 70.698 | 1.544 | 0.456 | 3.386 |
| HADH | 34.293 | 1.137 | 0.863 | 1.317 |
| UGP2 | 56.94 | 1.104 | 0.896 | 1.232 |
| DGUOK | 32.055 |  |  |  |
| TXNRD1 | 70.905 | 1.658 | 0.342 | 4.848 |
| IMMT | 83.677 | 0.877 | 1.123 | 0.781 |
| HNRNPUL2 | 85.104 | 1 |  |  |
| INF2 | 135.62 | 1.374 | 0.626 | 2.195 |
| PDS5A | 150.83 |  |  |  |
| AAK1 | 103.88 | 1 |  |  |
| WASHC4 | 136.4 | 1 |  |  |
| IAH1 | 27.598 | 1 |  |  |
| HLA-DRB1 | 30.004 | 1.209 | 0.791 | 1.528 |
| HLA-B | 40.4 |  |  |  |
| LRRFIP1 | 89.252 | 1 |  |  |
| P3H1 | 83.393 |  |  |  |
| EML3 | 95.196 | 1 |  |  |
| SLC25A35 | 32.437 |  |  |  |
| TKFC | 58.946 | 1.457 | 0.543 | 2.683 |
| HSDL1 | 37.001 | 1 |  |  |
| RABL6 | 79.548 |  |  |  |
| TUBB8 | 49.775 | 0.948 | 1.052 | 0.901 |
| LGALSL | 18.986 | 1.629 | 0.371 | 4.391 |
| GLYR1 | 60.547 | 1 |  |  |
| GTDC1 | 52.596 | 1 |  |  |
| VPS26B | 39.154 | 1 |  |  |
| TBC1D10B | 87.198 | 1 |  |  |
| ANO6 | 106.16 | 1.85 | 0.15 | 12.333 |
| SVEP1 | 390.17 | 1 |  |  |
| CCDC58 | 16.62 | 1 |  |  |
| PDCD4 | 51.735 | 1 |  |  |
| HSD17B12 | 34.324 | 1.38 | 0.62 | 2.226 |
| AGK | 47.137 |  |  |  |
| LACTB2 | 32.805 | 1 |  |  |
| EIPR1 | 43.603 | 1 |  |  |
| ACTBL2 | 42.003 | 1.508 | 0.492 | 3.065 |
| PAN3 | 95.612 | 1 |  |  |
| HSP90AB4P | 58.264 |  |  |  |
| YIF1B | 34.435 | 1 |  |  |
| HERC4 | 118.56 |  |  |  |
| PPP6R3 | 97.668 | 0.909 | 1.091 | 0.833 |
| DDX60L | 197.67 | 1 |  |  |
| EARS2 | 58.688 |  | 1 |  |
| PITRM1 | 117.41 | 1.179 | 0.821 | 1.436 |
| WDR44 | 101.37 | 1.587 | 0.413 | 3.843 |
| DOCK11 | 237.67 | 1 |  |  |
| TOR1AIP1 | 66.248 |  |  |  |
| GNAS | 111.02 | 1 |  |  |
| SAMD9 | 184.28 | 1 |  |  |
| WDR45B | 38.121 | 1 |  |  |
| WIPI1 | 48.673 |  |  |  |
| TTC38 | 52.787 | 1 |  |  |
| LY6G6F | 32.464 | 1.253 | 0.747 | 1.677 |
| FKBP15 | 133.63 | 1 |  |  |
| ARMH3 | 78.71 | 1 |  |  |
| HECTD3 | 97.112 | 1.205 | 0.795 | 1.516 |
| UBR4 | 573.83 | 1.009 | 0.991 | 1.018 |
| ARHGAP21 | 217.46 | 1 |  |  |
| EMC10 | 27.347 |  |  |  |
| YOD1 | 38.322 | 1.569 | 0.431 | 3.64 |
| BROX | 46.476 | 1.67 | 0.33 | 5.061 |
| HACD4 | 27.52 | 1 |  |  |
| PEAR1 | 110.67 | 1.711 | 0.289 | 5.92 |
| ECPAS | 204.29 | 1 |  |  |
| DENND4C | 212.71 | 1 |  |  |
| FAM160B1 | 86.557 | 1 |  |  |
| SPRYD7 | 21.666 | 1 |  |  |
| MICOS13 | 13.087 | 1 |  |  |
| RNF213 | 591.4 | 1.31 | 0.69 | 1.899 |
| STEAP3 | 54.6 | 1.782 | 0.218 | 8.174 |
| FAM91A1 | 93.908 | 1 |  |  |
| LARP1B | 105.32 | 1 |  |  |
| TBC1D9B | 140.52 |  |  |  |
| MAP1S | 112.21 | 0.951 | 1.049 | 0.907 |
| PPP2R2D | 52.042 |  |  |  |
| RAP1GAP2 | 80.055 |  |  |  |
| MBLAC2 | 31.371 |  |  |  |
| ARHGAP17 | 95.436 | 1 |  |  |
| ATL3 | 60.541 | 1.477 | 0.523 | 2.824 |
| VASN | 71.712 |  | 1 |  |
| PTRHD1 | 15.805 | 1 |  |  |
| NADSYN1 | 79.284 |  |  |  |
| DHRS7B | 35.119 |  |  |  |
| TWF2 | 39.548 | 1.306 | 0.694 | 1.882 |
| TANGO2 | 30.937 |  |  |  |
| SULT6B1 | 34.918 |  | 1 |  |
| PDE12 | 67.351 |  |  |  |
| SLC25A24 | 53.354 | 0.708 | 1.292 | 0.548 |
| HIBCH | 43.482 | 1.068 | 0.932 | 1.146 |
| LPCAT3 | 56.034 |  |  |  |
| SLC27A4 | 72.063 | 1.283 | 0.717 | 1.789 |
| SCYL2 | 103.71 |  |  |  |
| TTC27 | 96.631 |  |  |  |
| PDXDC2P | 51.809 | 1 |  |  |
| PLBD1 | 63.254 |  |  |  |
| MEAK7 | 50.993 | 1 |  |  |
| EMB | 36.881 | 1 |  |  |
| PGM2L1 | 70.441 | 1 |  |  |
| TTC37 | 175.48 | 1 |  |  |
| DARS2 | 73.562 | 1 |  |  |
| NCEH1 | 45.807 | 0.426 | 1.574 | 0.271 |
| TMED8 | 35.74 | 1 |  |  |
| POTEE | 121.36 | 1 |  |  |
| CCDC171 | 152.81 |  | 1 |  |
| CYP20A1 | 52.432 |  |  |  |
| TMEM205 | 21.198 |  |  |  |
| SUSD1 | 82.709 |  |  |  |
| LCLAT1 | 48.92 | 1 |  |  |
| OLFM4 | 57.279 |  |  |  |
| PLXDC2 | 59.582 | 1.459 | 0.541 | 2.697 |
| PI16 | 49.471 |  | 1 |  |
| CRELD2 | 38.191 |  |  |  |
| APOOL | 29.159 |  |  |  |
| PACS1 | 104.9 |  |  |  |
| NAPRT | 57.578 |  |  |  |
| CD109 | 161.69 | 1.436 | 0.564 | 2.546 |
| HSDL2 | 45.394 | 1 |  |  |
| NBEAL2 | 302.51 | 1.59 | 0.41 | 3.878 |
| MFSD6 | 88.087 | 1 |  |  |
| ARHGEF18 | 151.64 | 1 |  |  |
| TMPPE | 49.452 | 1 |  |  |
| BARGIN | 73.599 | 1 |  |  |
| PIK3AP1 | 90.397 |  |  |  |
| LHFPL2 | 24.486 |  |  |  |
| NUDT18 | 35.501 | 1 |  |  |
| TOM1L2 | 55.556 | 1 |  |  |
| MBOAT2 | 59.526 | 1 |  |  |
| VPS13C | 422.39 | 1.795 | 0.205 | 8.756 |
| MOB2 | 26.926 | 1 |  |  |
| UNC13D | 123.28 | 1.48 | 0.52 | 2.846 |
| TUBA1A | 50.135 | 0.717 | 1.283 | 0.559 |
| SND1 | 102 | 1.769 | 0.231 | 7.658 |
| BZW1 | 48.043 |  |  |  |
| LRRC8D | 98.2 | 1 |  |  |
| ASRGL1 | 32.054 | 1 |  |  |
| EIF3M | 42.502 | 1.516 | 0.484 | 3.132 |
| RRAGA | 36.566 |  |  |  |
| CYFIP1 | 145.18 | 1.629 | 0.371 | 4.391 |
| DOK3 | 53.288 |  |  |  |
| GET4 | 36.504 | 1 |  |  |
| COPS6 | 36.163 | 1.728 | 0.272 | 6.353 |
| EPM2AIP1 | 70.369 | 1 |  |  |
| FASTKD5 | 86.573 | 1 |  |  |
| RASGRP2 | 69.248 | 1.162 | 0.838 | 1.387 |
| PHF5A | 12.405 | 1 |  |  |
| ERMP1 | 100.23 |  |  |  |
| ZC3HAV1 | 101.43 | 1 |  |  |
| TRAPPC11 | 128.88 | 1 |  |  |
| ATG9A | 94.446 | 1 |  |  |
| DGLUCY | 66.436 | 1 |  |  |
| VPS35L | 109.56 | 1 |  |  |
| MON2 | 190.36 | 1 |  |  |
| DENND4A | 209.24 | 1 |  |  |
| MYH14 | 227.87 |  |  |  |
| DHX29 | 155.23 | 1 |  |  |
| POGLUT3 | 58.572 | 1 |  |  |
| DCXR | 25.913 | 1.366 | 0.634 | 2.155 |
| BRAP | 67.304 | 1 |  |  |
| HUWE1 | 481.89 | 1.721 | 0.279 | 6.168 |
| TMED4 | 25.943 | 1 |  |  |
| GALNT7 | 75.388 | 1 |  |  |
| GLRX5 | 16.628 | 1 |  |  |
| PRUNE1 | 50.199 | 1.622 | 0.378 | 4.291 |
| MTDH | 63.836 | 1 |  |  |
| TSPAN33 | 31.538 | 1.417 | 0.583 | 2.431 |
| KTN1 | 156.27 | 1 |  |  |
| NLRX1 | 107.61 | 1.653 | 0.347 | 4.764 |
| FERMT3 | 75.952 | 1.248 | 0.752 | 1.66 |
| ALYREF | 26.888 |  |  |  |
| GPR180 | 49.395 |  |  |  |
| CD163 | 125.45 | 1 |  |  |
| NRAP | 197.07 |  | 1 |  |
| VPS36 | 43.816 | 1.752 | 0.248 | 7.065 |
| CAND1 | 136.37 | 1.323 | 0.677 | 1.954 |
| RETREG3 | 51.396 | 1 |  |  |
| HOOK3 | 83.125 | 1 |  |  |
| COMTD1 | 28.808 | 1 |  |  |
| NLRP8 | 119.43 |  |  |  |
| PKHD1L1 | 465.73 | 1.615 | 0.385 | 4.195 |
| CCDC25 | 24.479 | 1 |  |  |
| TMEM173 | 42.192 | 1 |  |  |
| NIT1 | 35.896 |  |  |  |
| HEATR5A | 222 | 1 |  |  |
| ANGPTL5 | 44.144 |  | 1 |  |
| NDUFA11 | 14.852 |  |  |  |
| ERO1B | 53.543 |  |  |  |
| C2CD5 | 110.45 | 1 |  |  |
| RASAL3 | 111.9 | 1 |  |  |
| TREML1 | 32.678 | 0.891 | 1.109 | 0.803 |
| HIST2H2AB | 13.995 |  |  |  |
| CRLF3 | 49.765 | 1.394 | 0.606 | 2.3 |
| TRAPPC5 | 20.783 | 1 |  |  |
| PLD3 | 54.705 | 1 |  |  |
| SLC9A9 | 72.564 | 1.619 | 0.381 | 4.249 |
| NAXD | 36.576 | 1 |  |  |
| SLC44A2 | 80.123 | 1.678 | 0.322 | 5.211 |
| WDFY1 | 46.323 | 1.663 | 0.337 | 4.935 |
| UBR1 | 200.21 |  |  |  |
| RHOT2 | 68.117 |  |  |  |
| PARP9 | 96.342 | 1 |  |  |
| CMIP | 86.33 |  |  |  |
| DIS3L2 | 99.278 | 1 |  |  |
| ANKRD13A | 67.618 | 1 |  |  |
| ALDH16A1 | 85.126 | 1.323 | 0.677 | 1.954 |
| ABI1 | 55.08 | 1.741 | 0.259 | 6.722 |
| ORMDL3 | 17.494 |  |  |  |
| VPS52 | 82.22 |  |  |  |
| NUP93 | 93.487 |  |  |  |
| LRRC47 | 63.472 | 1 |  |  |
| CA13 | 29.443 |  |  |  |
| ARMC10 | 37.54 |  |  |  |
| ARHGAP18 | 74.976 |  |  |  |
| GUF1 | 74.327 | 1 |  |  |
| PNKD | 42.875 |  |  |  |
| PIP4P2 | 28.081 |  |  |  |
| ZADH2 | 40.14 | 1 |  |  |
| OXR1 | 97.969 | 1 |  |  |
| MINDY1 | 51.777 | 1.643 | 0.357 | 4.602 |
| CISD2 | 15.278 |  |  |  |
| JAGN1 | 21.125 |  |  |  |
| MYCT1 | 26.592 | 1.23 | 0.77 | 1.597 |
| C1orf52 | 20.599 |  | 1 |  |
| EMC1 | 111.76 | 1.521 | 0.479 | 3.175 |
| ANKRD44 | 107.6 | 1 |  |  |
| ENAH | 66.509 | 1 |  |  |
| ATP6V0D2 | 40.426 | 1 |  |  |
| TC2N | 55.284 |  |  |  |
| GATD1 | 23.297 | 1 |  |  |
| NHLRC2 | 79.443 | 1.695 | 0.305 | 5.557 |
| SUMF2 | 33.843 |  |  |  |
| PCYOX1L | 54.646 |  |  |  |
| TMEM87A | 63.429 | 1 |  |  |
| HSD17B11 | 32.935 | 0.869 | 1.131 | 0.768 |
| TXNDC5 | 47.628 | 1.501 | 0.499 | 3.008 |
| SCCPDH | 47.151 | 1 |  |  |
| PDPR | 99.363 | 1 |  |  |
| SMG8 | 109.68 | 1 |  |  |
| CCNY | 39.336 | 1 |  |  |
| BANK1 | 89.281 | 1 |  |  |
| MAPK1IP1L | 24.269 |  |  |  |
| RGS22 | 147.16 |  | 1 |  |
| MCU | 39.866 | 1.548 | 0.452 | 3.425 |
| APPL2 | 74.493 | 1.791 | 0.209 | 8.569 |
| SLC30A7 | 41.625 | 1 |  |  |
| DOCK8 | 238.53 | 1.81 | 0.19 | 9.526 |
| SYNE1 | 1011.1 |  |  |  |
| TRIM58 | 54.766 | 1.349 | 0.651 | 2.072 |
| TSPAN14 | 30.69 | 1.549 | 0.451 | 3.435 |
| MOSPD2 | 59.745 | 1 |  |  |
| GIMAP7 | 34.508 | 1 |  |  |
| RPLP0P6 | 34.364 | 0.805 | 1.195 | 0.674 |
| OCC1 | 6.4069 |  |  |  |
| NPLOC4 | 68.119 |  |  |  |
| RIN3 | 107.85 | 1 |  |  |
| UBA3 | 51.852 |  |  |  |
| FAM174A | 19.954 | 1 |  |  |
| PIP4K2C | 47.299 | 1 |  |  |
| TBC1D15 | 79.49 | 1.633 | 0.367 | 4.45 |
| RDH11 | 35.386 | 1.471 | 0.529 | 2.781 |
| IFI44 | 50.49 | 1 |  |  |
| NT5C | 23.382 |  |  |  |
| ZFAND1 | 30.787 | 1 |  |  |
| STT3B | 93.673 | 1.577 | 0.423 | 3.728 |
| SPPL2A | 58.143 |  |  |  |
| HM13 | 41.488 |  |  |  |
| DYNC2LI1 | 39.624 |  |  |  |
| NEK9 | 107.17 |  |  |  |
| DTX3L | 83.553 | 1 |  |  |
| GNPDA2 | 31.084 |  |  |  |
| NEK7 | 34.551 |  |  |  |
| MICAL1 | 117.87 | 1.717 | 0.283 | 6.067 |
| DTD1 | 23.423 | 1.595 | 0.405 | 3.938 |
| NUP210 | 205.11 |  |  |  |
| UBASH3B | 72.695 | 1.774 | 0.226 | 7.85 |
| GIPC3 | 33.981 |  |  |  |
| MZB1 | 20.694 | 1 |  |  |
| SCFD2 | 75.126 | 1 |  |  |
| SMAP2 | 46.785 | 1 |  |  |
| TBC1D22A | 59.12 | 1 |  |  |
| RAB2B | 24.214 | 1.503 | 0.497 | 3.024 |
| PDCD6IP | 96.022 | 1.488 | 0.512 | 2.906 |
| BRK1 | 8.7448 | 1 |  |  |
| THEM6 | 23.865 |  |  |  |
| TRAPPC12 | 79.374 |  |  |  |
| UBLCP1 | 36.804 | 1 |  |  |
| ITLN1 | 34.961 | 1.43 | 0.57 | 2.509 |
| TMEM40 | 25.495 | 0.25 | 1.75 | 0.143 |
| SLC44A1 | 73.301 | 1.558 | 0.442 | 3.525 |
| GIMAP1 | 34.369 |  |  |  |
| STON2 | 101.16 |  |  |  |
| ATL1 | 63.543 | 1.356 | 0.644 | 2.106 |
| MADD | 183.3 | 1 |  |  |
| ADGRV1 | 693.06 | 1 |  |  |
| BRI3BP | 27.835 | 1 |  |  |
| SEPT1 | 41.97 | 1 |  |  |
| CFHR4 | 65.35 |  |  |  |
| DDX1 | 82.431 |  |  |  |
| SLC39A7 | 50.117 | 1 |  |  |
| FAM3C | 24.68 |  |  |  |
| PSMF1 | 29.816 | 1.419 | 0.581 | 2.442 |
| GBF1 | 206.44 | 1 |  |  |
| NCSTN | 78.41 | 1 |  |  |
| ELMO1 | 83.829 |  |  |  |
| AP3S1 | 21.732 | 1 |  |  |
| HSPH1 | 96.864 | 1 |  |  |
| CNOT9 | 33.631 |  |  |  |
| TBC1D5 | 89.003 |  |  |  |
| MYO18A | 233.11 | 1.723 | 0.277 | 6.22 |
| ARHGAP45 | 124.61 | 1.771 | 0.229 | 7.734 |
| RABGGTA | 65.071 |  |  |  |
| TFG | 43.447 |  |  |  |
| ARPC1A | 41.569 | 1 |  |  |
| TAF15 | 61.829 |  |  |  |
| GGH | 35.964 | 0.682 | 1.318 | 0.517 |
| INPP5D | 133.29 |  |  |  |
| SEMA4D | 96.149 |  |  |  |
| OSTF1 | 23.787 | 1.741 | 0.259 | 6.722 |
| ARHGEF1 | 102.43 |  |  |  |
| UFD1 | 34.5 | 1 |  |  |
| GLG1 | 134.55 | 1 |  |  |
| UPF1 | 124.34 |  |  |  |
| HPS1 | 79.291 | 1 |  |  |
| COPS5 | 37.578 |  |  |  |
| RAB8B | 23.584 |  |  |  |
| KHSRP | 73.114 |  |  |  |
| PRG4 | 151.06 |  | 1 |  |
| TNFRSF14 | 30.391 | 1 |  |  |
| TNPO1 | 102.35 | 1.535 | 0.465 | 3.301 |
| ARHGEF2 | 111.54 | 1 |  |  |
| USP9X | 292.28 |  |  |  |
| USP7 | 128.3 | 1 |  |  |
| ATP6V0A1 | 96.412 | 1.711 | 0.289 | 5.92 |
| LPP | 65.746 |  |  |  |
| ATP2A3 | 113.98 | 1.582 | 0.418 | 3.785 |
| PHKB | 124.88 | 1 |  |  |
| HLA-B | 40.416 | 1.032 | 0.968 | 1.066 |
| MYDGF | 18.795 | 1.557 | 0.443 | 3.515 |
| TOMM40L | 33.916 | 1 |  |  |
| OSBP2 | 101.26 | 1 |  |  |
| WBP2 | 28.087 | 1.579 | 0.421 | 3.751 |
| PSMG2 | 29.396 | 1 |  |  |
| NCLN | 62.974 | 1 |  |  |
| RILPL2 | 23.986 | 1 |  |  |
| ERGIC1 | 32.592 | 1.678 | 0.322 | 5.211 |
| TBRG4 | 70.737 |  |  |  |
| CCDC47 | 55.873 | 1 |  |  |
| EXOC4 | 110.5 |  |  |  |
| ISOC2 | 22.337 | 1 |  |  |
| LRRC59 | 34.93 | 1 |  |  |
| ESAM | 41.176 | 1.288 | 0.712 | 1.809 |
| RPE | 24.927 | 1 |  |  |
| VPS33A | 67.61 |  |  |  |
| RAB37 | 24.815 | 1.554 | 0.446 | 3.484 |
| ISG20 | 20.363 | 1 |  |  |
| ARL8A | 21.416 | 1.213 | 0.787 | 1.541 |
| TESC | 24.75 | 0.933 | 1.067 | 0.874 |
| DOCK10 | 249.53 | 1.299 | 0.701 | 1.853 |
| EFHD2 | 26.697 | 1.459 | 0.541 | 2.697 |
| GALM | 37.765 |  |  |  |
| SYTL4 | 76.023 | 1.596 | 0.404 | 3.95 |
| DCPS | 38.608 | 1 |  |  |
| ACSF2 | 68.124 | 1 |  |  |
| ISOC1 | 32.236 | 1.12 | 0.88 | 1.273 |
| AP2M1 | 49.654 | 1 |  |  |
| KCTD12 | 35.7 | 1 |  |  |
| REPS1 | 86.661 | 1 |  |  |
| SNRNP40 | 39.31 | 1 |  |  |
| ERLEC1 | 54.858 | 1 |  |  |
| CMTM5 | 24.652 | 1.589 | 0.411 | 3.866 |
| RAB3C | 25.952 | 1 |  |  |
| SEH1L | 39.648 |  |  |  |
| L3HYPDH | 38.137 | 1 |  |  |
| CYFIP2 | 148.4 | 1 |  |  |
| DYNLL2 | 10.35 |  |  |  |
| CPNE2 | 61.189 | 1 |  |  |
| SCRN2 | 46.596 | 1 |  |  |
| OTUB1 | 31.284 | 1.593 | 0.407 | 3.914 |
| PGM2 | 68.283 | 1.452 | 0.548 | 2.65 |
| CERS2 | 44.876 |  |  |  |
| DCUN1D1 | 30.124 | 1 |  |  |
| SNF8 | 28.864 | 1 |  |  |
| PDLIM5 | 63.944 | 1.768 | 0.232 | 7.621 |
| CRELD1 | 45.439 | 1 |  |  |
| ERO1A | 54.392 | 1.232 | 0.768 | 1.604 |
| AHCYL2 | 66.72 | 1 |  |  |
| DHTKD1 | 103.08 | 1 |  |  |
| ZNF625 | 34.746 |  |  |  |
| SUCLG2 | 46.51 |  |  |  |
| GMPPA | 46.291 | 1.631 | 0.369 | 4.42 |
| ABHD14B | 22.345 | 1.424 | 0.576 | 2.472 |
| NGLY1 | 74.389 | 1 |  |  |
| ATP5MD | 6.4575 |  |  |  |
| CPB2 | 48.424 | 0.021 | 1.979 | 0.011 |
| THOC3 | 38.771 | 1 |  |  |
| ITCH | 102.8 |  |  |  |
| COG3 | 94.095 |  |  |  |
| CDK5RAP3 | 56.92 | 1 |  |  |
| VPS39 | 101.81 |  |  |  |
| SPG11 | 278.86 |  |  |  |
| RNF170 | 29.814 | 1 |  |  |
| SLC35E1 | 44.772 |  |  |  |
| TMEM87B | 63.535 | 1 |  |  |
| USP47 | 157.31 |  |  |  |
| CNDP1 | 56.705 |  | 1 |  |
| EXOC2 | 104.07 | 1 |  |  |
| CNDP2 | 52.878 | 1.844 | 0.156 | 11.821 |
| SNX27 | 61.264 |  |  |  |
| PRRC1 | 46.701 | 1 |  |  |
| FGD4 | 86.625 |  |  |  |
| RIPOR3 | 105.29 |  |  |  |
| MBOAT7 | 52.764 | 1 |  |  |
| SFXN2 | 36.231 | 1 |  |  |
| NLRP3 | 118.17 |  |  |  |
| ARAP1 | 162.19 | 1.638 | 0.362 | 4.525 |
| IPO9 | 115.96 |  |  |  |
| RCHY1 | 30.11 | 1 |  |  |
| TRAPPC9 | 128.53 | 1 |  |  |
| TRNT1 | 50.127 |  |  |  |
| VPS35 | 91.706 | 1.499 | 0.501 | 2.992 |
| TSPAN32 | 34.631 |  |  |  |
| PANX1 | 48.05 | 1 |  |  |
| FCRL5 | 106.44 |  | 1 |  |
| F2RL3 | 41.133 |  |  |  |
| VPS13A | 360.27 | 1.831 | 0.169 | 10.834 |
| ERGIC2 | 42.548 | 1 |  |  |
| ERBIN | 158.3 | 1.104 | 0.896 | 1.232 |
| METTL26 | 22.578 | 1 |  |  |
| MYADM | 35.273 |  |  |  |
| ADO | 29.751 | 1 |  |  |
| RUFY1 | 79.817 | 1.796 | 0.204 | 8.804 |
| MMS19 | 113.29 |  |  |  |
| RMDN3 | 52.118 |  |  |  |
| TBCB | 27.325 |  |  |  |
| PSMB7 | 29.965 |  |  |  |
| CNN2 | 33.697 | 0.673 | 1.327 | 0.507 |
| PSMD1 | 105.84 | 1.284 | 0.716 | 1.793 |
| PFDN5 | 17.328 | 1 |  |  |
| PARK7 | 19.891 | 0.957 | 1.043 | 0.918 |
| GAS2L1 | 72.716 | 1 |  |  |
| SORT1 | 92.067 | 1.746 | 0.254 | 6.874 |
| VAT1 | 41.92 |  |  |  |
| EIF3C | 105.34 | 1 |  |  |
| PHB2 | 33.296 | 1.367 | 0.633 | 2.16 |
| COPS8 | 23.225 | 1 |  |  |
| CHP1 | 22.456 | 1 |  |  |
| MAP3K5 | 154.54 |  |  |  |
| MGLL | 33.261 | 1.518 | 0.482 | 3.149 |
| LYST | 429.13 | 1 |  |  |
| DOK1 | 52.391 |  |  |  |
| HSD17B10 | 26.923 | 0.858 | 1.142 | 0.751 |
| SEPT5 | 42.777 | 0.938 | 1.062 | 0.883 |
| NAP1L4 | 42.823 |  |  |  |
| MGST2 | 16.62 | 1 |  |  |
| ACO2 | 85.424 | 1.313 | 0.687 | 1.911 |
| TM9SF2 | 75.775 | 1.753 | 0.247 | 7.097 |
| TSG101 | 43.944 |  |  |  |
| CPNE1 | 59.058 | 1.525 | 0.475 | 3.211 |
| CCT7 | 59.366 | 1.599 | 0.401 | 3.988 |
| HIST1H2AJ | 13.936 |  |  |  |
| HIST1H2BL | 13.952 | 0.573 | 1.427 | 0.402 |
| AGPAT1 | 31.716 | 1 |  |  |
| SH3GL1 | 41.489 | 1 |  |  |
| RARRES2 | 18.617 | 0.887 | 1.113 | 0.797 |
| AKAP9 | 452.98 | 1 |  |  |
| CYBC1 | 20.774 |  |  |  |
| VKORC1 | 18.234 | 1 |  |  |
| TUBA1C | 49.895 | 0.579 | 1.421 | 0.407 |
| FYCO1 | 166.98 | 1 |  |  |
| CORO1B | 54.234 | 1.049 | 0.951 | 1.103 |
| TXNDC17 | 13.941 | 1.495 | 0.505 | 2.96 |
| CPPED1 | 35.548 | 1.573 | 0.427 | 3.684 |
| VPS25 | 20.747 | 1 |  |  |
| PAAF1 | 42.19 | 1 |  |  |
| ADPGK | 54.088 | 1.118 | 0.882 | 1.268 |
| MIEN1 | 12.403 | 1 |  |  |
| TRIM56 | 81.487 |  |  |  |
| ERP44 | 46.971 | 1.614 | 0.386 | 4.181 |
| LXN | 25.75 | 0.996 | 1.004 | 0.992 |
| NIPSNAP3B | 28.313 | 1 |  |  |
| TACO1 | 32.477 |  |  |  |
| HDHD3 | 28 |  |  |  |
| TUBGCP2 | 102.53 | 1 |  |  |
| ESYT1 | 122.85 | 1.383 | 0.617 | 2.241 |
| CCM2 | 48.836 | 1 |  |  |
| CNPY3 | 30.748 |  |  |  |
| LIMD2 | 14.07 |  | 1 |  |
| COPS4 | 46.268 | 1 |  |  |
| TMEM43 | 44.875 | 1.422 | 0.578 | 2.46 |
| TBCD | 132.6 |  |  |  |
| FUCA2 | 54.066 |  |  |  |
| TUBB6 | 49.857 | 1 |  |  |
| PAXX | 21.639 | 1.783 | 0.217 | 8.217 |
| PDCD10 | 24.701 |  |  |  |
| MENT | 36.769 |  | 1 |  |
| DERL1 | 28.8 |  |  |  |
| HTATIP2 | 27.049 | 1 |  |  |
| APOO | 22.284 | 1 |  |  |
| MRI1 | 39.149 |  |  |  |
| TUBB2B | 49.953 | 1 |  |  |
| MLST8 | 35.876 | 1 |  |  |
| TMEM109 | 26.21 |  |  |  |
| PBDC1 | 26.056 | 1 |  |  |
| PTDSS2 | 56.252 |  |  |  |
| TMED9 | 27.277 | 1.462 | 0.538 | 2.717 |
| TARS2 | 81.035 | 1 |  |  |
| ACAT2 | 41.35 | 1.189 | 0.811 | 1.466 |
| SFXN3 | 35.503 | 1.628 | 0.372 | 4.376 |
| CHID1 | 44.94 |  |  |  |
| GTPBP2 | 65.768 | 1.514 | 0.486 | 3.115 |
| TAPBPL | 50.182 | 1 |  |  |
| JAM3 | 35.02 | 1.256 | 0.744 | 1.688 |
| HINT2 | 17.162 | 1.157 | 0.843 | 1.372 |
| NAA15 | 101.27 | 1 |  |  |
| BCL2L13 | 52.723 | 1 |  |  |
| SRRT | 100.67 | 1 |  |  |
| AP1M1 | 48.586 | 1.246 | 0.754 | 1.653 |
| GON7 | 10.859 | 1 |  |  |
| ITPA | 21.445 |  |  |  |
| FUT8 | 66.515 |  |  |  |
| FAM126A | 57.625 | 1 |  |  |
| PITPNM2 | 148.93 | 1 |  |  |
| OSBPL8 | 101.19 | 1 |  |  |
| WDR11 | 136.68 | 1 |  |  |
| TBL1XR1 | 55.594 |  |  |  |
| FAM129A | 103.13 | 1 |  |  |
| UBXN6 | 49.753 | 1 |  |  |
| TM6SF1 | 41.636 | 1 |  |  |
| UBE2O | 141.29 | 1.848 | 0.152 | 12.158 |
| LNPK | 47.739 |  |  |  |
| TTYH3 | 57.544 | 1 |  |  |
| MTMR12 | 86.147 | 1.872 | 0.128 | 14.625 |
| PITHD1 | 24.178 |  |  |  |
| WDR61 | 33.58 |  |  |  |
| SLIRP | 12.349 |  |  |  |
| CCDC90B | 29.505 |  |  |  |
| NIF3L1 | 41.968 | 1 |  |  |
| MFF | 38.464 |  |  |  |
| SLC22A4 | 62.154 | 1 |  |  |
| PAIP1 | 53.524 | 1 |  |  |
| NT5C3A | 37.948 | 1.611 | 0.389 | 4.141 |
| TMEM222 | 23.23 | 1 |  |  |
| RAB1B | 22.171 | 1.41 | 0.59 | 2.39 |
| THAP2 | 26.26 |  | 1 |  |
| C11orf54 | 35.117 |  |  |  |
| FAM234A | 59.659 | 1 |  |  |
| MUC3B | 131.4 |  |  |  |
| TMX4 | 38.952 | 1 |  |  |
| WDR13 | 53.695 |  |  |  |
| EHD4 | 61.174 | 1 |  |  |
| VPS33B | 70.584 | 1 |  |  |
| VPS16 | 94.693 |  |  |  |
| VPS11 | 107.84 | 1 |  |  |
| SH3BGRL3 | 10.438 | 1.016 | 0.984 | 1.033 |
| SLK | 142.69 | 1 |  |  |
| PPIL3 | 18.154 | 1.767 | 0.233 | 7.584 |
| TAOK3 | 105.4 | 1 |  |  |
| RAB3GAP2 | 155.98 | 1 |  |  |
| PPA2 | 37.92 | 1.308 | 0.692 | 1.89 |
| C11orf68 | 31.43 | 1 |  |  |
| BOLA2 | 10.116 | 1 |  |  |
| TMX1 | 31.791 | 1.52 | 0.48 | 3.167 |
| ACBD3 | 60.593 |  |  |  |
| UNC45A | 103.08 | 1.609 | 0.391 | 4.115 |
| MFSD1 | 51.208 |  |  |  |
| DNAJC5 | 22.149 |  |  |  |
| FN3K | 35.171 | 1.246 | 0.754 | 1.653 |
| POFUT1 | 43.955 | 1 |  |  |
| RNPEP | 72.595 |  |  |  |
| GLIPR2 | 17.218 |  |  |  |
| EHD1 | 60.626 | 1.521 | 0.479 | 3.175 |
| RGCC | 14.559 |  | 1 |  |
| SLA2 | 28.585 | 1 |  |  |
| LDAH | 37.318 |  |  |  |
| SMYD3 | 49.097 | 1 |  |  |
| AAMDC | 13.332 | 1 |  |  |
| DOCK5 | 215.31 | 1 |  |  |
| WDR26 | 72.123 | 1 |  |  |
| VSIR | 33.908 | 1 |  |  |
| ACAD9 | 68.76 | 1.006 | 0.994 | 1.012 |
| METTL7A | 28.319 | 1.281 | 0.719 | 1.782 |
| MOB1A | 25.079 | 1.432 | 0.568 | 2.521 |
| PLEKHF2 | 27.797 | 1 |  |  |
| GORASP2 | 47.145 |  |  |  |
| PSTPIP2 | 38.858 | 1.564 | 0.436 | 3.587 |
| SFXN1 | 35.619 | 1.571 | 0.429 | 3.662 |
| VPS37B | 31.307 | 1 |  |  |
| L2HGDH | 50.315 | 1 |  |  |
| FN3KRP | 34.412 |  |  |  |
| CARS2 | 62.223 | 1 |  |  |
| PPCS | 34.005 | 1.141 | 0.859 | 1.328 |
| SIAE | 58.314 | 1.546 | 0.454 | 3.405 |
| GNB4 | 37.567 | 1.364 | 0.636 | 2.145 |
| CACYBP | 26.21 |  |  |  |
| RRAGC | 44.223 |  |  |  |
| RHOF | 23.625 | 1.216 | 0.784 | 1.551 |
| PARVG | 37.485 |  |  |  |
| PARVB | 41.714 | 1.51 | 0.49 | 3.082 |
| PLGRKT | 17.201 | 1 |  |  |
| TMEM165 | 34.905 | 1 |  |  |
| APOBEC3G | 46.407 | 1.359 | 0.641 | 2.12 |
| EML4 | 108.91 |  |  |  |
| GLOD4 | 34.793 | 1.7 | 0.3 | 5.667 |
| MCCC2 | 61.332 | 1.272 | 0.728 | 1.747 |
| GBA2 | 104.65 | 1 |  |  |
| PLXNA4 | 212.45 | 1 |  |  |
| GP6 | 36.866 | 1.583 | 0.417 | 3.796 |
| ATP13A1 | 132.95 | 1 |  |  |
| TM9SF3 | 67.887 | 1.497 | 0.503 | 2.976 |
| RETN | 11.419 | 1 |  |  |
| APMAP | 46.48 | 0.708 | 1.292 | 0.548 |
| TXNRD2 | 56.506 | 1.516 | 0.484 | 3.132 |
| RAB18 | 22.977 | 1.324 | 0.676 | 1.959 |
| VTA1 | 33.879 | 1.54 | 0.46 | 3.348 |
| SARS2 | 58.282 |  |  |  |
| GPCPD1 | 76.034 |  |  |  |
| OSGEP | 36.426 |  |  |  |
| ISYNA1 | 61.067 | 1 |  |  |
| BRD7 | 74.138 |  | 1 |  |
| CASS4 | 87.143 |  |  |  |
| CRTAC1 | 71.42 |  | 1 |  |
| RTN4 | 129.93 | 1.633 | 0.367 | 4.45 |
| PFDN4 | 15.314 | 1 |  |  |
| NIT2 | 30.608 |  |  |  |
| KIF13B | 202.79 | 1 |  |  |
| XPNPEP1 | 69.917 | 1.406 | 0.594 | 2.367 |
| PDLIM7 | 49.844 | 1.713 | 0.287 | 5.969 |
| ACSS2 | 78.579 | 1 |  |  |
| SAR1A | 22.367 | 0.982 | 1.018 | 0.965 |
| MAN1C1 | 70.91 |  |  |  |
| SH3GLB2 | 43.973 | 1 |  |  |
| EIF2B3 | 50.24 | 1 |  |  |
| MBNL1 | 41.817 | 1 |  |  |
| PDGFC | 39.029 | 1 |  |  |
| PRTFDC1 | 25.673 |  |  |  |
| TUBG2 | 51.091 |  |  |  |
| AASDHPPT | 35.776 | 1 |  |  |
| OSTC | 16.829 |  |  |  |
| HEBP1 | 21.097 | 1 |  |  |
| RAB6B | 23.461 | 0.928 | 1.072 | 0.866 |
| APOBEC3C | 22.826 | 1 |  |  |
| DUSP22 | 20.91 |  |  |  |
| VPS45 | 65.076 |  |  |  |
| PHPT1 | 13.832 | 1.33 | 0.67 | 1.985 |
| SERINC1 | 50.494 | 1 |  |  |
| ARHGAP35 | 170.51 | 1 |  |  |
| FAM114A2 | 55.468 | 1 |  |  |
| RGS18 | 27.582 | 1.32 | 0.68 | 1.941 |
| TOMM22 | 15.521 | 1 |  |  |
| LANCL2 | 50.854 |  |  |  |
| FARSB | 66.115 | 1 |  |  |
| IARS2 | 113.79 | 0.428 | 1.572 | 0.272 |
| ATG3 | 35.864 |  |  |  |
| MAN2C1 | 115.83 |  |  |  |
| SACM1L | 66.966 | 1.238 | 0.762 | 1.625 |
| OLA1 | 44.743 | 0.757 | 1.243 | 0.609 |
| SLC35D1 | 39.24 | 1 |  |  |
| ACSS1 | 74.856 |  |  |  |
| ABHD10 | 33.932 |  |  |  |
| RYDEN | 33.11 | 1 |  |  |
| FAM49B | 36.748 | 1.597 | 0.403 | 3.963 |
| GIMAP4 | 37.533 | 0.723 | 1.277 | 0.566 |
| TBC1D23 | 78.321 |  |  |  |
| TMEM30A | 40.683 | 1.485 | 0.515 | 2.883 |
| SEPT11 | 49.398 | 1.681 | 0.319 | 5.27 |
| PANK4 | 85.99 | 1 |  |  |
| TBC1D13 | 46.553 |  |  |  |
| DNAJC11 | 63.277 | 1 |  |  |
| NECAP2 | 28.338 | 1 |  |  |
| ANO10 | 76.328 |  |  |  |
| HIF1AN | 40.285 |  |  |  |
| CZIB | 18.048 |  |  |  |
| BABAM1 | 36.56 | 1 |  |  |
| NDUFB11 | 17.316 |  |  |  |
| OCIAD1 | 27.626 | 1.15 | 0.85 | 1.353 |
| CMTM6 | 20.419 | 1 |  |  |
| TOR4A | 46.914 | 1.344 | 0.656 | 2.049 |
| SIDT1 | 93.838 | 1 |  |  |
| BABAM2 | 43.551 |  |  |  |
| STAB1 | 275.48 | 1 |  |  |
| DPP3 | 82.588 | 1.353 | 0.647 | 2.091 |
| TUBA8 | 50.093 | 0.655 | 1.345 | 0.487 |
| TMOD3 | 39.594 | 1.379 | 0.621 | 2.221 |
| UGGT1 | 177.19 | 1.053 | 0.947 | 1.112 |
| TECR | 36.034 |  |  |  |
| ERAP1 | 107.23 | 1.427 | 0.573 | 2.49 |
| ACTR10 | 46.306 | 1 |  |  |
| CLIC5 | 46.502 |  |  |  |
| FAM120A | 121.89 | 1.391 | 0.609 | 2.284 |
| EHD3 | 60.886 | 1.434 | 0.566 | 2.534 |
| C1RL | 53.498 |  |  |  |
| NCKIPSD | 78.959 | 1.522 | 0.478 | 3.184 |
| OGFR | 73.324 | 1 |  |  |
| COMMD9 | 21.819 |  |  |  |
| CNIH4 | 16.093 | 1 |  |  |
| HACD3 | 43.159 | 1.522 | 0.478 | 3.184 |
| NDUFA13 | 16.698 | 1 |  |  |
| PDP1 | 61.053 |  |  |  |
| KCMF1 | 41.945 | 1 |  |  |
| VAPA | 27.893 | 1.622 | 0.378 | 4.291 |
| ORMDL1 | 17.371 |  |  |  |
| TMEM14C | 11.564 |  |  |  |
| CLEC1B | 26.595 | 1.219 | 0.781 | 1.561 |
| ABRACL | 9.0564 |  |  |  |
| CALCOCO1 | 77.335 | 1 |  |  |
| VPS18 | 110.18 |  |  |  |
| RCC2 | 56.084 | 1.206 | 0.794 | 1.519 |
| RELCH | 134.63 | 1 |  |  |
| STK26 | 46.528 | 1 |  |  |
| HEATR5B | 224.3 | 1 |  |  |
| RRBP1 | 152.45 | 0.992 | 1.008 | 0.984 |
| TBC1D14 | 78.136 | 1 |  |  |
| ANKFY1 | 128.4 | 1 |  |  |
| SUCLA2 | 50.317 | 1.516 | 0.484 | 3.132 |
| DPM3 | 10.094 | 1 |  |  |
| ATXN10 | 53.488 |  |  |  |
| EPS15L1 | 94.254 | 1.36 | 0.64 | 2.125 |
| COPG2 | 97.621 | 1 |  |  |
| COMMD3 | 22.151 | 1 |  |  |
| HDAC6 | 131.42 |  |  |  |
| VPS29 | 20.505 | 1.201 | 0.799 | 1.503 |
| EIF3K | 25.059 |  |  |  |
| GRHPR | 35.668 | 1.435 | 0.565 | 2.54 |
| CTSZ | 33.868 |  |  |  |
| DNAJB11 | 40.513 | 1.515 | 0.485 | 3.124 |
| UBA2 | 71.223 | 1 |  |  |
| PEF1 | 30.381 |  |  |  |
| BIN2 | 61.874 | 1.919 | 0.081 | 23.691 |
| UQCR10 | 7.3084 |  |  |  |
| TJP2 | 133.96 | 1 |  |  |
| STK39 | 59.473 | 1 |  |  |
| ADD3 | 79.154 | 1.738 | 0.262 | 6.634 |
| NIPSNAP3A | 28.466 | 1.712 | 0.288 | 5.944 |
| PRKAG2 | 63.065 | 1 |  |  |
| FETUB | 42.054 |  | 1 |  |
| ARMCX3 | 42.5 | 1.653 | 0.347 | 4.764 |
| SUN2 | 80.31 | 0.431 | 1.569 | 0.275 |
| LAMTOR3 | 13.623 |  |  |  |
| NDOR1 | 66.762 | 1 |  |  |
| SRP68 | 70.729 | 1 |  |  |
| CHORDC1 | 37.489 | 1.676 | 0.324 | 5.173 |
| TBK1 | 83.641 | 1 |  |  |
| PCYOX1 | 56.639 | 0.206 | 1.794 | 0.115 |
| SHPK | 51.49 | 1 |  |  |
| DPP7 | 54.341 | 1.407 | 0.593 | 2.373 |
| BCAP29 | 28.32 |  |  |  |
| PFDN2 | 16.648 | 1.277 | 0.723 | 1.766 |
| NRBP1 | 59.844 | 1 |  |  |
| ATP6V1H | 55.882 | 1 |  |  |
| XPO7 | 123.91 | 1.602 | 0.398 | 4.025 |
| CD84 | 38.782 | 0.78 | 1.22 | 0.639 |
| AK3 | 25.565 | 1 |  |  |
| LNPEP | 117.35 |  |  |  |
| RABGEF1 | 56.89 | 1 |  |  |
| MSRA | 26.132 | 1.423 | 0.577 | 2.466 |
| NAGK | 37.375 | 1.568 | 0.432 | 3.63 |
| SH3BGRL2 | 12.326 | 1.364 | 0.636 | 2.145 |
| SLC25A13 | 74.175 | 1.288 | 0.712 | 1.809 |
| DBNL | 48.207 | 0.759 | 1.241 | 0.612 |
| STOML2 | 38.534 |  |  |  |
| SERPINA10 | 50.706 |  |  |  |
| APPL1 | 79.663 | 1.437 | 0.563 | 2.552 |
| NUDT5 | 24.327 | 1 |  |  |
| VAV3 | 97.775 |  |  |  |
| RAB21 | 24.347 | 1.421 | 0.579 | 2.454 |
| RAB22A | 21.855 |  |  |  |
| TRAPPC2L | 16.145 | 1 |  |  |
| PSME2 | 27.401 | 1.521 | 0.479 | 3.175 |
| MCTS1 | 20.555 | 1 |  |  |
| ACSL5 | 75.99 |  |  |  |
| ASAP1 | 125.5 |  |  |  |
| PLXNB3 | 206.84 |  |  |  |
| CORO1C | 53.248 | 1.383 | 0.617 | 2.241 |
| PYCARD | 21.627 | 1 |  |  |
| PRPF19 | 55.18 | 1 |  |  |
| UBQLN1 | 62.518 | 1 |  |  |
| DAPP1 | 32.193 | 1.664 | 0.336 | 4.952 |
| VPS4A | 48.897 | 1 |  |  |
| PACSIN2 | 55.738 |  |  |  |
| MAGED2 | 64.953 | 1 |  |  |
| SNX6 | 46.648 |  |  |  |
| SSR3 | 21.08 |  |  |  |
| PSMD13 | 42.945 | 1.699 | 0.301 | 5.645 |
| PROCR | 26.671 |  | 1 |  |
| PPIE | 33.43 | 1 |  |  |
| COPS3 | 47.873 | 1.306 | 0.694 | 1.882 |
| NSFL1C | 40.572 | 0.252 | 1.748 | 0.144 |
| MACF1 | 838.3 | 1.778 | 0.222 | 8.009 |
| PPP6R1 | 96.723 | 1 |  |  |
| EXOC7 | 83.381 | 1 |  |  |
| USP24 | 294.36 | 1.591 | 0.409 | 3.89 |
| DNM3 | 97.745 | 1 |  |  |
| PA2G4 | 43.786 | 1.319 | 0.681 | 1.937 |
| MTMR6 | 71.967 | 1 |  |  |
| TRAF3IP3 | 63.626 | 1 |  |  |
| RUVBL2 | 51.156 |  |  |  |
| HIGD1A | 10.143 |  |  |  |
| HPSE | 61.148 | 1.034 | 0.966 | 1.07 |
| KCNK6 | 33.747 |  |  |  |
| CHKB | 45.271 | 1 |  |  |
| EIF3L | 66.726 | 1.622 | 0.378 | 4.291 |
| PLAA | 87.156 | 1.807 | 0.193 | 9.363 |
| RUVBL1 | 50.227 |  |  |  |
| ST3GAL6 | 38.213 | 1.183 | 0.817 | 1.448 |
| VDAC3 | 30.658 | 1.501 | 0.499 | 3.008 |
| FARSA | 57.563 | 1 |  |  |
| SIGLEC7 | 51.142 | 1 |  |  |
| TRAPPC4 | 24.34 | 1 |  |  |
| NCKAP1 | 128.79 | 1.909 | 0.091 | 20.978 |
| CNPY2 | 20.652 | 1.705 | 0.295 | 5.78 |
| DLGAP4 | 108.01 |  |  |  |
| EPB41L3 | 120.68 | 1 |  |  |
| ATP8A1 | 131.37 | 1.489 | 0.511 | 2.914 |
| GSTK1 | 25.497 | 1.227 | 0.773 | 1.587 |
| LAMTOR2 | 13.507 | 1 |  |  |
| CRYL1 | 35.419 | 1.456 | 0.544 | 2.676 |
| AP3M1 | 46.939 |  |  |  |
| LEMD3 | 99.996 | 1 |  |  |
| NOP58 | 59.578 |  | 1 |  |
| GIT1 | 84.34 |  |  |  |
| PRG3 | 25.405 | 1 |  |  |
| SUGT1 | 41.024 | 1.46 | 0.54 | 2.704 |
| ACOT9 | 49.901 | 1.312 | 0.688 | 1.907 |
| DERA | 35.23 | 1 |  |  |
| MEMO1 | 33.733 |  |  |  |
| LSM2 | 10.834 |  |  |  |
| SIGLEC9 | 50.081 |  |  |  |
| SH3GLB1 | 40.796 |  |  |  |
| CAB39 | 39.869 | 1.377 | 0.623 | 2.21 |
| LUC7L2 | 46.513 | 1 |  |  |
| UBE2J1 | 35.198 | 1.134 | 0.866 | 1.309 |
| DHRS7 | 38.298 | 1.401 | 0.599 | 2.339 |
| MOB4 | 26.032 |  |  |  |
| SBDS | 28.763 |  |  |  |
| TMED5 | 26.005 |  |  |  |
| TMED7 | 25.171 | 1.672 | 0.328 | 5.098 |
| PPIL1 | 18.237 |  |  |  |
| UFC1 | 19.458 | 1.407 | 0.593 | 2.373 |
| FIS1 | 16.937 | 1 |  |  |
| STRAP | 38.438 | 1.562 | 0.438 | 3.566 |
| RTCB | 55.21 | 1.311 | 0.689 | 1.903 |
| RABGAP1 | 121.74 | 1.868 | 0.132 | 14.152 |
| TMED3 | 24.777 |  |  |  |
| SAMHD1 | 72.2 | 1.555 | 0.445 | 3.494 |
| ATP6V0A2 | 98.081 | 1 |  |  |
| TLN1 | 269.76 | 1.014 | 0.986 | 1.028 |
| KIF3A | 80.04 | 1 |  |  |
| DAAM1 | 123.47 | 1.287 | 0.713 | 1.805 |
| USP15 | 112.42 | 1.547 | 0.453 | 3.415 |
| TLN2 | 271.61 |  |  |  |
| MYO5A | 215.4 | 0.317 | 1.683 | 0.188 |
| CRYBG1 | 188.67 |  | 1 |  |
| TRAF6 | 59.573 |  |  |  |
| MAP4K5 | 95.023 | 1 |  |  |
| HYOU1 | 111.33 | 1.114 | 0.886 | 1.257 |
| ARIH1 | 64.117 | 1 |  |  |
| LSM5 | 9.9374 | 1 |  |  |
| SAMM50 | 51.976 | 1.291 | 0.709 | 1.821 |
| HSPB11 | 16.297 | 1 |  |  |
| PPME1 | 42.315 | 1.72 | 0.28 | 6.143 |
| AP4S1 | 17.005 | 1 |  |  |
| TNPO3 | 104.2 | 1 |  |  |
| SRPRB | 29.702 | 1.632 | 0.368 | 4.435 |
| GMPPB | 39.834 | 1.639 | 0.361 | 4.54 |
| CDC42BPB | 194.31 | 1.655 | 0.345 | 4.797 |
| RBM8A | 19.889 | 1 |  |  |
| IER3IP1 | 8.9687 | 1 |  |  |
| SNX9 | 66.591 |  |  |  |
| SNX5 | 46.816 | 1 |  |  |
| FHOD1 | 126.55 | 1.55 | 0.45 | 3.444 |
| SLC25A15 | 32.736 |  |  |  |
| F11R | 32.583 | 1.339 | 0.661 | 2.026 |
| NPTN | 44.387 |  |  |  |
| COPG1 | 97.717 | 1.128 | 0.872 | 1.294 |
| CLIC4 | 28.772 | 1.518 | 0.482 | 3.149 |
| SPCS1 | 11.805 |  |  |  |
| EMILIN1 | 106.69 | 1.424 | 0.576 | 2.472 |
| MTCH2 | 33.331 | 1.327 | 0.673 | 1.972 |
| ARFGEF2 | 202.04 |  |  |  |
| ARFGEF1 | 208.76 | 1.707 | 0.293 | 5.826 |
| STK24 | 49.307 | 1.471 | 0.529 | 2.781 |
| MRVI1 | 97.929 | 1 |  |  |
| TEX264 | 34.188 | 1 |  |  |
| OAS3 | 121.17 | 1 |  |  |
| NDUFB9 | 21.831 |  |  |  |
| SQOR | 49.96 | 1.249 | 0.751 | 1.663 |
| FCGBP | 572.01 | 0.522 | 1.478 | 0.353 |
|  |  |  |  |  |
