# Supplementary material for: Integrated proteome and phosphoproteome analyses of peripheral blood mononuclear cells in primary Sjögren syndrome patients
Source: Aging (Albany NY). 2020 Dec 3;13(1):1071–95. doi: 10.18632/aging.202233 (PMC7835054; doi:10.18632/aging.202233)
Supplement: Supplementary Tables 2 and 3 [file aging-13-202233-s003.pdf]

SUPPLEMENTARY TABLES

Supplementary Table 2. Clinical characteristics of the primary Sjögren's syndrome (pSS) and normal control (NC) subjects.

	pSS Age Group (30-65 years old)	NC Age Group (30-65 years old)
Number of Subjects	8	10
Age, mean ± SD years old	46±6.2	43.2±6.01
Sex, % female	100%	100%
Disease duration, mean ± SD years	6.2±2.7	N/A
% UWSF (≤1.5 ml/15 min)	60%	0%
Mean UWSF (ml/15 min)	1.67±1.2	3.9±1.7
% + Schirmer's test (≤5mm/5min)	80%	0%
Mean Schirmer (mm/5min)	5.2±7.9	25.4±8.08
% anti-SSA antibodies	100%	0%
% anti-SSB antibodies	80%	0%
Mean RF	200±80	N/A

Recruitment of pSS subjects was based on strict American European Consensus Group (AECG) criteria. Non-smoking adult pSS female subjects were screened for oral and ocular dryness, positive labial gland biopsy and positive serological tests for SSA and SSB antibodies. Age-matched, non-smoking adult normal control (NC) females were screened for good general and oral health. *UWSF=Unstimulated whole saliva flow; RF=Rheumatoid factor; N/A= Not applicable.*

Supplementary Table 3. The materials and reagents required for the sample preparation.

Materials and reagents	Supplier
protease inhibitor	Calbiochem
ultrapure water (H2O)	Fisher Chemical
acetonitrile	Fisher Chemical
formic acid	Fluka
phosphorylase inhibitor	Millipore
trypsin	Promega
iodoacetamide	Sigma
urea	Sigma
dithiothreitol	Sigma
TEAB	Sigma
trifluoroacetic acid	Sigma-Aldrich
BCA kit	Beyotime
